# Tumor-associated macrophages drive heterogenetic CD10^High^ cancer stem cells to implement tumor-associated neutrophils reprogramming in oral squamous cell carcinoma

**DOI:** 10.7150/ijbs.100611

**Published:** 2025-01-13

**Authors:** Yuanhe You, Zhong Du, Zhuowei Tian, Shunshun Li, Fan Yu, Meng Xiao, Yue He, Yanan Wang

**Affiliations:** 1 Department of Oral and Maxillofacial-Head and Neck Oncology, Shanghai Ninth People's Hospital, Shanghai Jiao Tong University, School of Medicine, Shanghai 200011, China.; 2 College of Stomatology, Shanghai Jiao Tong University, Shanghai 200011, China.; 3 National Center for Stomatology, National Clinical Research Center for Oral Disease, Shanghai 200011, China.; 4 Shanghai Key Laboratory of Stomatology, Shanghai 200011, China.

**Keywords:** tumor-associated macrophage, cancer stem cells, tumor-associated neutrophil, CD10, immune reprogramming

## Abstract

Tumor-associated macrophages (TAMs) in the tumor microenvironment (TME) widely participate in the malignant progression in cancer. Previously, we have demonstrated that M1-like TAMs cascaded a stem-like phenotype of oral squamous cell carcinoma (OSCC). Yet, the underlying mechanisms still need to be demonstrated for the regulation of TAMs on cancer stem cells (CSCs) in OSCC. In this study, we investigated a group of CSCs with increased expression of cluster differentiation 10 (CD10), which acted as a mediator in the interaction network between TAMs and tumor-associated neutrophils (TANs) in OSCC. The results showed a significant association between TAMs infiltrations and increased expression of CD10 among all the CSCs-related molecules in OSCC. Then, we validated that OSCC cells with high CD10 expression possessed increased CSCs characteristics. TAMs could drive the heterogenetic CD10^High^ CSCs by activating the IL6/STAT3/CD10 pathway. Furthermore, CD10^High^ CSCs could recruit and reprogram tumor-associated neutrophils (TANs) in an immunosuppressive state by secreting S100A8/A9 in OSCC. These finding indicated that CD10^High^ CSCs played great roles in signaling crosstalk between TAMs and TANs in OSCC, by which infiltrated TAMs drive CD 10^High^ CSCs to recruit and reprogram TANs in an immunosuppressive state. Herein, we managed to demonstrate that TAMs could directly regulate a heterogenetic cluster of CSCs with high CD10 expression, and CD10^High^ CSCs could recruit and reprogram TANs in OSCC. The novel crosstalk among OSCC-TAMs-CD10^High^ CSCs-TANs might bring new prospects for improving the treatment strategies for OSCC patients.

## 1. Introduction

Oral squamous cell carcinoma (OSCC), a major component of head and neck squamous cell carcinoma (HNSCC), has been considered as one of the most lethal malignant tumors worldwide 1. Till now, treatment failure for OSCC patients could result from cancer recurrence, metastasis and chemo/radiotherapy resistance 2. Cancer stem cells (CSCs) have been demonstrated to be widely participated in the treatment failure for OSCC 3-5. Unfortunately, no effective treatment has been developed based on CSCs for OSCC. Previous studies on CSCs have always focused on identifying CSC biomarkers, which appeared to be inconsistent across different studies for OSCC 6. Recently, an increasing number of studies have suggested that CSCs should be identified as dynamic clusters rather than a stable population of cancer cells 7. The heterogeneous and dynamic nature of CSCs has been identified as a primary factor contributing to the failure in treatment translation by targeting CSCs 5. The stemness of CSCs is not only determined by the intrinsic genetic heterogeneous factors of cancer cells, but also the extrinsic factors from tumor microenvironment (TME) 8, 9. Till now, numerous omics studies have failed to identify the definitive genetic driver mutations in OSCC. So, TME might play a crucial role in regulating CSCs behaviors in OSCC 10, 11. In the TME, tumor-associated macrophages (TAMs) are the most abundant immune cells. TAMs could regulate angiogenesis, immune suppression, metastasis, and treatment resistance 12, 13. In addition, several studies have demonstrated the ability of TAMs to directly modulate CSCs. In our previous study, we have reported that TAMs cascaded cancer cells into a mesenchymal/stem-like phenotype in OSCC via the IL6/STAT3/THBS1 feedback loop 14. Yet, the heterogeneous regulation of CSCs by TAMs remains to be elucidated in OSCC.

Heterogeneity of CSCs also exhibits in their biological behaviors and functions. Heterogenetic CSCs play variable roles in driving immune escape, metastasis and recurrence in the intricate network of crosstalk regulation between cancer cells and tumor-infiltrating immune cells 15, 16. Emerging evidence suggests that CSCs with distinct molecular phenotypes employ different pathways to the immune response, and resist anticancer therapy 17. Wang *et al.* found that CD276^High^ CSCs could directly suppress the infiltration of CD8^+^ T cells to facilitate immune escape 18. Jia *et al.* reported that targeting BMI1^+^ CSCs could activate cell-intrinsic immunity to allow immune checkpoint blockade, which could effectively hinder metastatic cancer cells and prevent cancer relapse 3. Undoubtedly, further studies should be done to uncover the underlying mechanism in the regulation of heterogenetic CSCs. In OSCC, studies have still focused on discover one or a panel of biomarkers to define CSCs for a long time, which greatly limit the clinical translation of CSCs related therapy for OSCC patients. What's more, recent studies have also indicated that CSCs are plastic cells, endowing with multiple molecular characteristics adapting to the varied TME. TAMs have been demonstrated to have a profound impact on the feedback regulation of CSCs in our previous study. In this study, we managed to identify a cluster of CSCs regulated by TAMs in OSCC. Subsequently, we also demonstrated the biological roles of TAMs related CSCs in OSCC.

## 2. Materials and Methods

### 2.1 Tissue microarray construction

Two experienced and certified pathologists thoroughly reviewed, diagnosed, and confirmed all the included primary OSCC samples. Sample size was determined by *MedCalc* software (version 23.02), choosing parameter: α=0.05, β=0.2, correlation coefficient=0.399 (result from TIMER algorithm). Accordingly, the minimal sample size was 47, and 48 primary OSCC samples were eventually included to detect the correlations between the expression of CSC related biomarkers and TAMs infiltration in OSCC. Tissue microarray (TMA) was constructed following a standardized protocol as previously described 19. This study was approved by the Ethics Committee of the Shanghai Ninth People's Hospital (SH9H-2024-T46-1).

### 2.2 Cell culture

The human OSCC cell lines, HN4, HN30, SCC9, Cal27, SCC25 and HN6, were cultured and used as previously described 14, 20. Cancer associated fibroblasts (CAFs) were isolated from primary HNSCC tumoral tissues, cultured in DMEM containing 10% BS and 1% PS. Normal human oral keratinocytes (HOK) were cultured in Oral Keratinocyte Medium (OKM, ScienCell) Human monocyte leukemia cell line (THP-1) was cultured in RPMI 1640 medium supplemented with 10% FBS. THP-1 Macrophages were induced from THP-1 cells as previously described 21. HL60 cells were cultured in IMDM supplemented with 20% fetal bovine serum (FBS). For HL60 cell differentiation, 0.5×10^6^ cells/mL were cultured in IMDM supplemented with 1.3% DMSO 22. After 5 days incubation, the differentiated HL60 neutrophils were harvested and used for the subsequent assays. All cells were cultured at 37°C in a humidified 5% CO_2_ atmosphere.

CD10-overexpressing plasmids, siSTAT3, shRNA targeting CD10, and related lentivirus were synthesized by Genomeditech Inc. (Beijing, China). The shRNA sequences targeting CD10 were as follows: shCD10-1: TGA CAA TGA TCG CAC TCT ATG; shCD10-2: CAA CCT ACG ATG ATG GTA TTT. The siRNA sequences targeting STAT3 were as follows: siSTAT3 (5'-3'), CGU CAU UAG CAG AAU CUC A tt, and UGA GAU UCU GCU AAU GAC G tt. OSCC cells were transfected by using the Lipofectamine® 3000 Transfection Kit (Invitrogen, USA) for 72h, and scrambled siRNA or vector plasmid was used as control. Transduction efficiency was further validated.

### 2.3 Immunohistochemical (IHC) staining and immunofluorescence (IF) staining

IHC staining was performed as previously described 21. Primary antibodies against CD10 (Cell Signaling Technology, USA), CD163 (Proteintech, USA), CD68 (Proteintech, USA), CD80 (Proteintech, USA), CD66b (Abmart, China), CD8 (Abcam, UK), pSTAT3 (Cell Signaling Technology, USA), Ki67 (Beyotime, China), and S100A8/A9 (Abcam, UK) were used. The immunoreaction score (IRS) was calculated by multiplying the percentage of positive cells by the staining intensity, as previously reported 23. For IF staining, CD10 (Cell Signaling Technology, USA), CD163 (Proteintech, USA), CD68 (Proteintech, USA), and CD80 (Proteintech, USA) were used. The nuclei were counterstained with DAPI.

### 2.4 Western Blotting (WB)

Samples were harvested by using a whole-cell lysis buffer containing a proteinase inhibitor cocktail (Pierce, USA) 24. Protein samples were electrophoresed and transferred as previously described 24. The incubated primary antibodies were used against: CD10 (Cell Signaling Technology, USA), pSTAT3 (Cell Signaling Technology, USA), STAT3 (Cell Signaling Technology, USA), p-p65 (Proteintech, USA), p65 (Cell Signaling Technology, USA), S100A8/A9 (Abcam, UK), pErk1/2 (Cell Signaling Technology, USA), Erk1/2 (Cell Signaling Technology, USA), Arg1 (Proteintech, USA), NOS2 (Proteintech, USA), PDL1 (Thermo, USA), and β-actin (Proteintech, USA).

### 2.5 Quantitative real-time polymerase chain reaction (qRT-PCR)

Total RNA was extracted and reverse-transcribed as previously described 14. cDNA was subjected to qRT-PCR using an ABI StepOne real-time PCR system (Life Technologies, USA). The relative expression levels of CD10, NOS2, CCL3, ICAM1, CXCL9, CXCL10, IFNG, ARG1, MMP9, TGFB, CCL2, CXCL1, CXCL16, VEGFA, IL8, CD274, GZMB, S100A8, S100A9, and ACTB were calculated and compared.

### 2.6 Fluorescence activated cell sorting (FACS)

HN6 cells were sorted into CD10^High^ and CD10^Low^ groups by FACS. Briefly, 1×10^7^ HN6 cells were washed and resuspended in 100 μL of staining buffer supplemented with anti-CD10-APC (Biolegend, USA). The cells were washed with staining buffer and sorted into CD10^High^ and CD10^Low^ groups using a FACS Aria II instrument (BD Biosciences). HN6-CD10^High^ and HN6-CD10^Low^ cells were harvested for further analysis.

### 2.7 CD10 activity

The activity of CD10 was determined as previously described 25. Briefly, cells were harvested and homogenized in 50 mM Tris-HCl buffer (pH 7.4). The homogenate was then centrifuged to remove the crude debris. Substrate solutions consisting of 1 mM DAGPNG (N-dansyl-Ala-Gly-d-nitro-Phe-Gly) and 10 μM enalaprile in 50 mM Tris-HCl and 10 μM phosphoramidon; (CD10 inhibitor) were prepared. The substrate solutions were incubated with each sample. The fluorescence of the supernatant was monitored at an emission wavelength of 562 nm and excitation wavelength of 342 nm. CD10 activity was calculated by the difference in fluorescence between the samples incubated with and without phosphoramidon.

### 2.8 Colony formation assay

The involved cells were suspended, counted, diluted, seeded at 1 000 cells/well in 6-well plates, and cultured for 10 days under different treatment conditions. Cellular colonies were fixed after washing and stained with 0.1% crystal violet for 30 min, after which the colonies were photographed and counted 14.

### 2.9 Microsphere formation assay

The involved cells were seeded at a density of 1 000 cells/well in 6-well low-adhesion plates (Corning, Inc., USA). Microspheres were cultured in DMEM/F12 supplemented with vitamin B27, heparin (4 mg/mL), and FGF (20 ng/mL) under different treatments. All visible spheres were imaged and counted after 10 days of continuous medium exposure 21.

### 2.10 Soft agar colony formation

Low-solubility agarose solutions (1.2% and 0.6%; Sigma, USA) were prepared using distilled water and sterilized. Three milliliters of the bottom agar layer containing 0.6% agarose in 2 × complete DMEM supplemented with 20% FBS was added to a 6-well plate and allowed to solidify for at least 30 min at room temperature. The upper agar layer was prepared by suspending 1000 cells in 3 ml of 0.3% agarose in 2 × high-glucose DMEM supplemented with 20% FBS. The upper agar layer was added to the solidified bottom agar layer and the cultures were incubated at 37°C for 15 days. The resulting colonies stained with nitrotetrazolium blue chloride were photographed and counted 26.

### 2.11 Chromatin immunoprecipitation (ChIP) and RT-PCR

ChIP assays were performed using the SimpleChIP Enzymatic Chromatin IP Kit (Cell Signaling Technology, USA). Four pairs of primers targeting the CD10 promoter region were designed and synthesized by Sangon Biotech (Shanghai, China): #1 forward, 5′- GTG AGT GTG CTG TGC AGT GAG -3'; reverse, 5′ - AAA ACT ACA AGG CTT TTG TAT TCC C -3'. An anti-STAT3 antibody (Cell Signaling Technology, USA) and IgG (Cell Signaling Technology, USA) were used.

### 2.12 RNA sequence and bioinformatics analysis

RNA-seq was performed on OSCC cells subjected to different treatments (OE Biotech Co., Ltd., Shanghai, China). As previously described, differentially expressed genes (DEGs) were identified using R package (q value≤0.05, fold change>2) 14. The filtered DEGs were filtered and further validated.

### 2.13 Human phospho-kinase array

The level of p-kinase was detected using a Proteome Profiler Human Phospho-Kinase Array Kit (R&D Systems, USA). OSCC cells were lysed after treatment with conditioned media (CM) from TAM-CM or M0-CM for 2h. Cell lysates were assayed according to the manufacturer's instructions.

### 2.14 Flow cytometry

The cells were harvested after incubation under different treatment conditions. The cells were stained with fluorochrome-conjugated antibodies. The antibodies used in this study were against CD66b, CD8, PDL1, PD1, Tim3, GZMB, and γ-IFN (Biolegend, USA). The stained cells were analyzed using a cytometer (BD FACS Calibur, USA).

### 2.15 *ELISA*

The expression of IL6, S100A8/9, Granzyme B, and γ-IFN levels in the cell supernatants were quantified using commercially available ELISA kits (Multiscience, China) according to the manufacturer's instructions.

### 2.16 Chemotaxis assay

Neutrophil chemotaxis was assessed by using transwell assays with a 3μm polycarbonate membrane. Neutrophils were seeded in the upper chamber, and CM from OSCC cells was added to the lower chamber after different treatments. After incubation at 37°C for 2h, the membrane was stained and the cells in the lower chamber were counted.

### 2.17 Xenograft

Four-week-old female BALB/c-nu mice were purchased from the Shanghai Laboratory Animal Center (Shanghai, China) and housed under SPF conditions following the procedure and approved by the Laboratory Animal Care and Use Committees of Shanghai Ninth People's Hospital, Shanghai Jiao Tong University School of Medicine. Briefly, nude mouse xenograft tumor models were established by subcutaneous injection of OSCC cells under different conditions as previously described. The tumor volumes (length × width^2^/2) were monitored and compared 24. The tumors were harvested for subsequent assays.

### 2.18 Statistical analyses

All statistical computations were performed by using SPSS software (version 20. 0; SPSS, Inc., Chicago, IL, USA) and GraphPad Prism (version 9; GraphPad Software, San Diego, CA, USA). For comparisons, Student's t-test was used for testing the statistical significance between two groups. Crosstab analyses and Chi-square tests were performed to analyze the correlations between two variables. Data were represented as the mean ± standard deviation (SD), and a *p*-value <0.05 was considered statistically significant.

## 3. Results

### 3.1 The expression of CD10 was significantly associated with TAMs infiltration in OSCC

**Figure 1:**
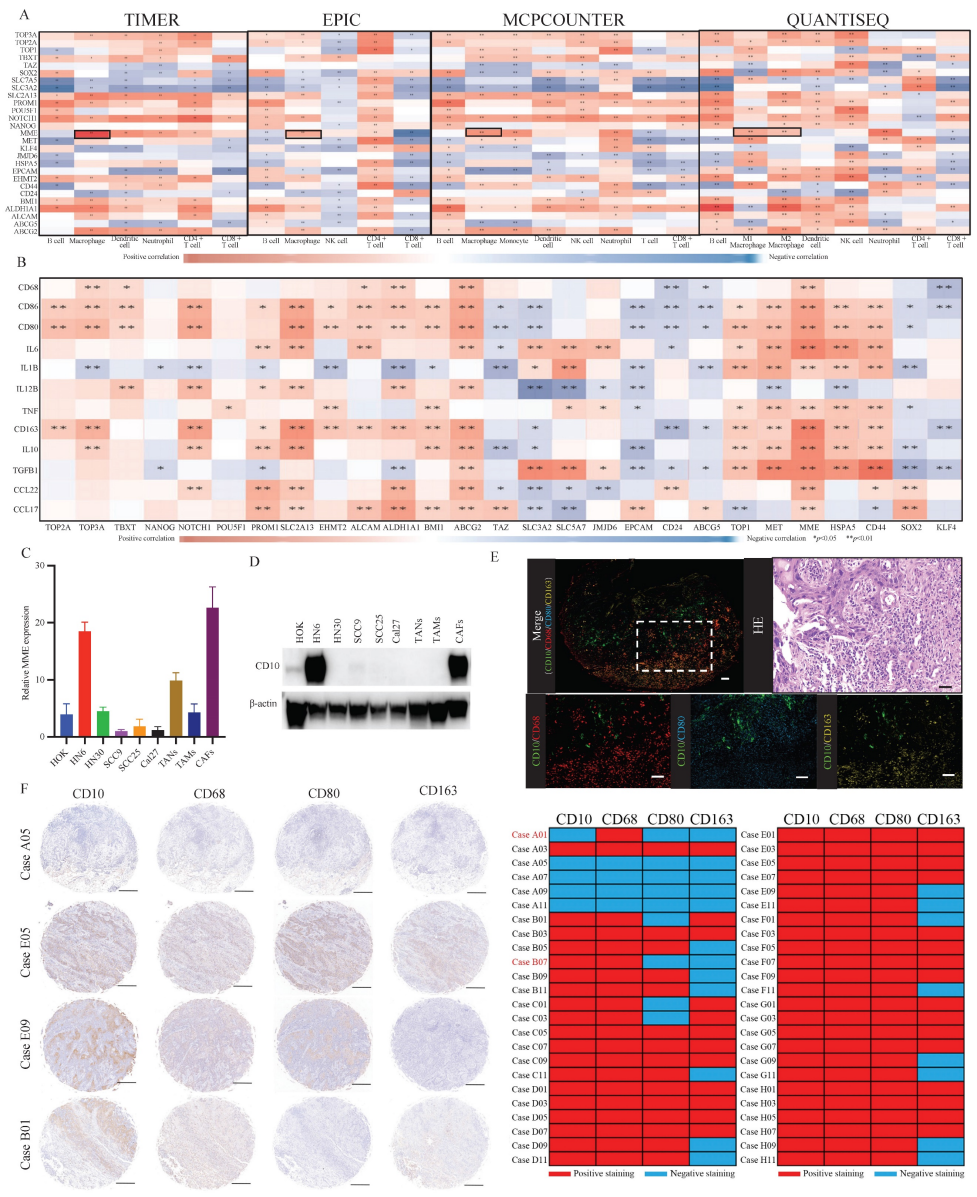
The expression of CD10 was significantly associated with TAMs infiltration in OSCC. (A) Relationships between the reported OSCC-CSC markers and immune cell infiltration via TIMER, EPIC, MCPCOUNTER, and QUANTISEQ algorithms; (B) Relationships between OSCC-CSC markers and TAMs related molecules based on the TCGA primary HNSCC cohort; (C,D) The mRNA expression level (C) and protein expression level (D) of CD10 in oral epithelial cells (HOK), OSCC cells (HN6, HN30, SCC9, SCC25 and Cal27), TANs, TAMs and CAFs; (E) Representative multiplex immunofluorescence staining results for CD10 (green), CD68 (red), or CD80 (blue) and with CD163 (yellow), scale ruler: 50μm; (F) Representative IHC images for CD10, CD68, CD80 and CD163 provided by the primary OSCC TMA (n=48); the relationship analysis was displayed, scale ruler: 500μm;*p<0.05, **p<0.01.

To assess the relationship between the expression of referenced CSCs-related molecules and the infiltration of immune cells, we performed immune analysis by using the TIMER, EPIC, MCPCOUNTER, and QUANTISEQ. Comparatively, significantly and stably positive correlations were observed between the expression of *MME* and macrophage infiltration (Fig. 1A). Besides, high *MME* expression was significantly correlated to the increased expression of TAMs-related molecules (Fig. 1B). To further understand the expression level of CD10, encoded by *MME*, in different cells of OSCC, we exerted a single-cell RNA expression analysis based on the OSCC GSE103322 dataset. Thereinto, we observed that CD10 could be highly expressed in a cluster of malignant cells (Supplementary file 1). Then, we assessed the expression level of CD10 in HOK, TANs, TAMs, CAFs and the OSCC cell lines (HN6, HN30, SCC9, SCC25, Cal27) (Fig. 1C-D), indicating that CD10 could be highly expressed in the OSCC cell line HN6. It is worth noting that the mutation rate of CD10 in head and neck squamous cell carcinoma (HNSCCC) is only 2.83% (Supplementary File 2). In situ multiple IF staining showed that the distribution of CD10 positive cells were mainly center on the OSCC cell, and tightly adjacent to the TAMs with CD68, CD80 and CD163 expression in OSCC patients (Fig. 1E). In our validated cohort, significant correlation was also observed between the expression of CD10 and the infiltration of TAMs in OSCC samples (n=48) (Fig. 1F, Supplementary File 3). The above information suggested that OSCC cells with high CD10 expression might be regulate by the infiltrated TAMs in OSCC.

### 3.2 CD10^High^ OSCC cells exhibited enhanced CSCs properties

**Figure 2:**
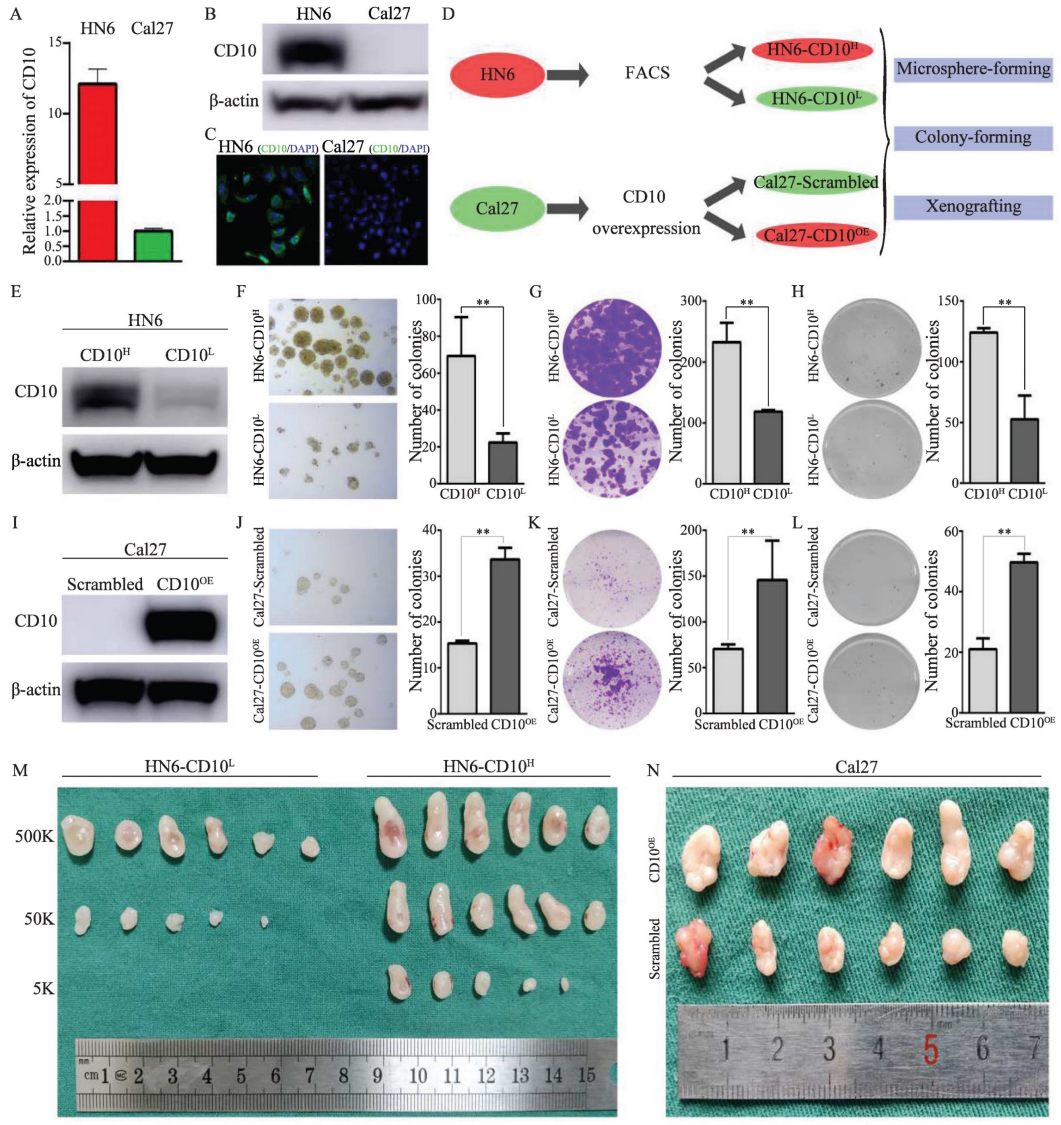
OSCC-CD10^High^ cells exhibited enhanced CSCs properties. (A) The mRNA levels of CD10 was determined in Cal27 and HN6 cells by qPCR; (B) The protein levels of CD10 was detected in Cal27 and HN6 cells by WB; (C) Immunofluorescence in situ hybridization of CD10 in Cal27 and HN6 cells; (D) Schematic diagram of the CD10-FACS and CD10-overexpressing workflow in OSCC cells; (E) HN6-CD10^High^ and HN6-CD10^Low^ cells were sorted by FACS and identified by Western blot; (F-H) The CSCs properties of the sorted HN6 cells were detected by microsphere formation (F), colony formation (G), and soft agar colony formation (H) assays; the data were presented as the means ± SDs of three independent experiments, **p<0.01; (I) Validation of CD10-overexpressing transfection efficiency in Cal27 cells; (J-L) The CSCs properties of CD10^High^ Cal27 cells were detected by microsphere formation (J), colony formation (K), and soft agar colony formation (L) assays. The data were presented as the means ± SDs of three independent experiments, **p<0.01. (M-N) *in vivo* xenografts of HN6 (M) or Cal27 (N) cells (n = 6).

CD10 has been reported as a candidate CSC biomarker. Herein, we performed a series of assays to validate the CSCs characteristics of CD10^High^ OSCC. Comparatively, HN6 cells possessed high expression of CD10, while Cal27 cells expressed relatively low level of CD10(Fig. 2A-C, supplementary File 4A). We then observed that HN6 cells had superior CSCs tumorigenic ability than Cal27 cells (supplementary File 4B). To confirm the roles of CD10 in regulating the CSCs properties of OSCC cells, we obtained CD10^High^ and CD10^Low^ subpopulations of HN6 cells by FACS and Cal27 cells by exogenous overexpression (Fig. 2D, Supplementary File 4C-D). HN6-CD10^High^ cells were shown to possess significantly increased CSCs properties (microsphere formation, colony formation and soft-agar colony formation) than the HN6-CD10^Low^ cells (Fig. 2E-H). So it was with the Cal27 cells with CD10-overexpression (Fig. 2I-L). Subsequent *in vivo* assays indicated that HN6-CD10^High^ cells exhibited significantly higher tumorigenicity (Fig. 2M, Supplementary File 4E). For the Cal27 cells with increased CD10 expression, significantly increased tumorigenicity was also observed *in vivo* (Fig. 2N, Supplementary File 4F). Based on the MACS model, we observed that increased CD10 expression greatly promoted the tumorigenicity of OSCC cells (Supplementary File 5). The above *in vitro* and *in vivo* assays indicated that CD10^High^ OSCC were a cluster of cells possessing potent CSC behaviors.

### 3.3 CD10 inhibition attenuated the CSCs properties of OSCC cells

**Figure 3:**
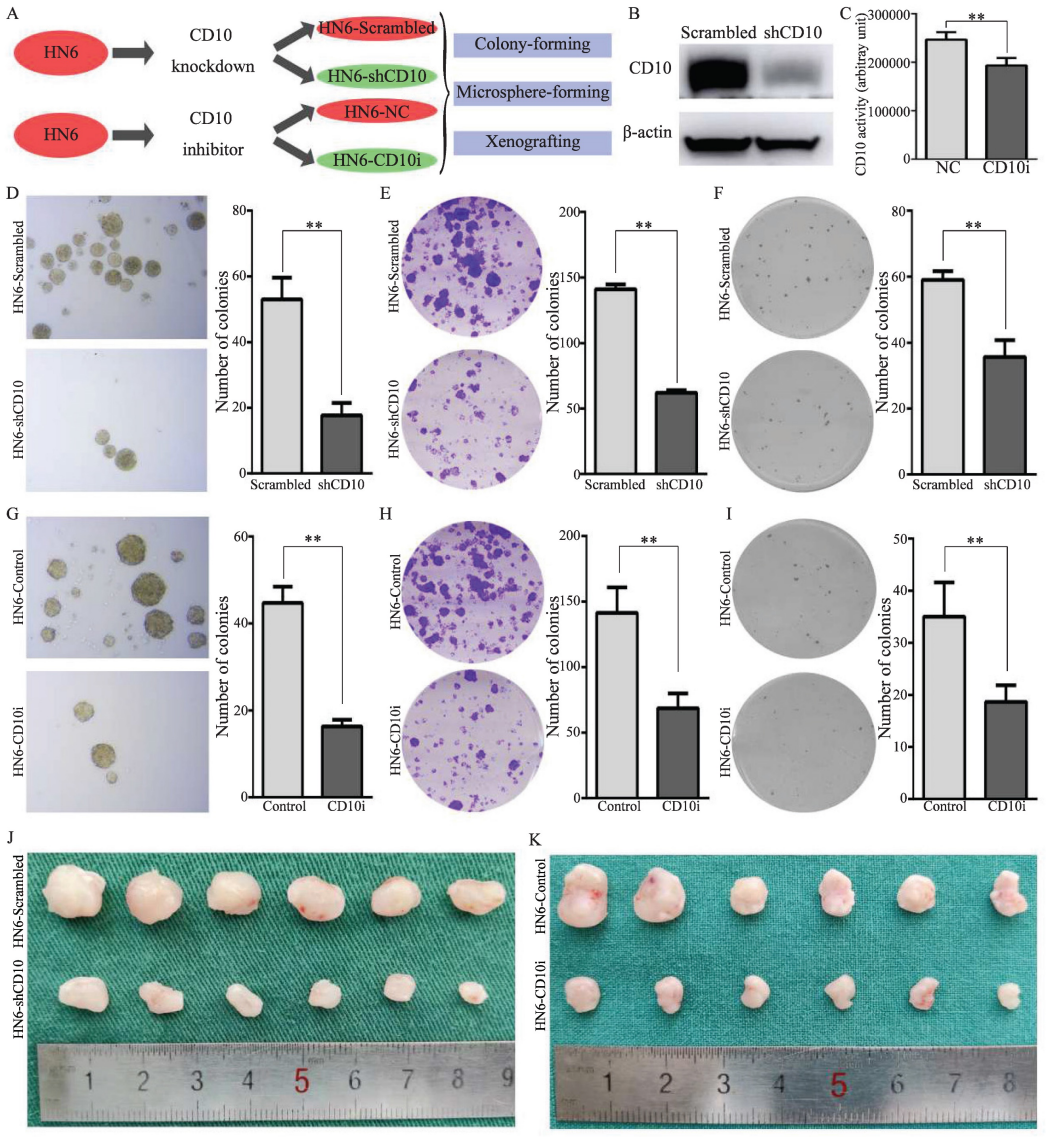
CD10 inhibition attenuated the cancer stemness and malignant behaviors of OSCC cells. (A) Schematic diagram of CD10 inhibition through genetic interference and pharmacological inhibition; (B) Transfection efficiency of shCD10 in HN6 cells detected by Western blot; (C) CD10 activity was measured in HN6 cells treated with CD10 inhibitor sacubitril, **p<0.01; (D-F) The CSCs properties of OSCC cells transfected with CD10 knockdown lentivirus was detected by microsphere formation (D), colony formation (E), and soft agar colony formation (F) assays. The data were presented as the means ± SDs of three independent experiments, **p<0.01; (G-I) The CSCs properties of OSCC cells treated with CD10 inhibitor was detected by microsphere formation (G), colony formation (H), and soft agar colony formation (I) assays. The data were presented as the means ± SDs of three independent experiments, **p<0.01; (J-K) Subcutaneous xenografts of HN6 cells transduced with shCD10 or treated with CD10 inhibitor (n = 6).

To further explore the effects of CD10 on the CSCs properties of OSCC cells, genetic and pharmacological rescue experiments were carried out. Firstly, shRNA transfection was used to decrease the expression of CD10 in HN6 cells, and CD10 inhibitors were used to inhibit the activity of CD10 in HN6 cells (Fig. 3A-C; Supplementary File 6). The rescue experiments showed that the inhibition of the expression or the activity of CD10 could significantly decrease the CSCs behaviors of HN6 cells, including microsphere formation, colony formation, soft-agar colony formation and *in vivo* xenografting (Fig. 3D-3K; Supplementary File 7-8). The above information further confirmed the roles of CD10 in regulating the CSC behaviors of OSCC cells.

### 3.4 TAMs drove the heterogenetic CD10^High^ CSCs in OSCC

**Figure 4:**
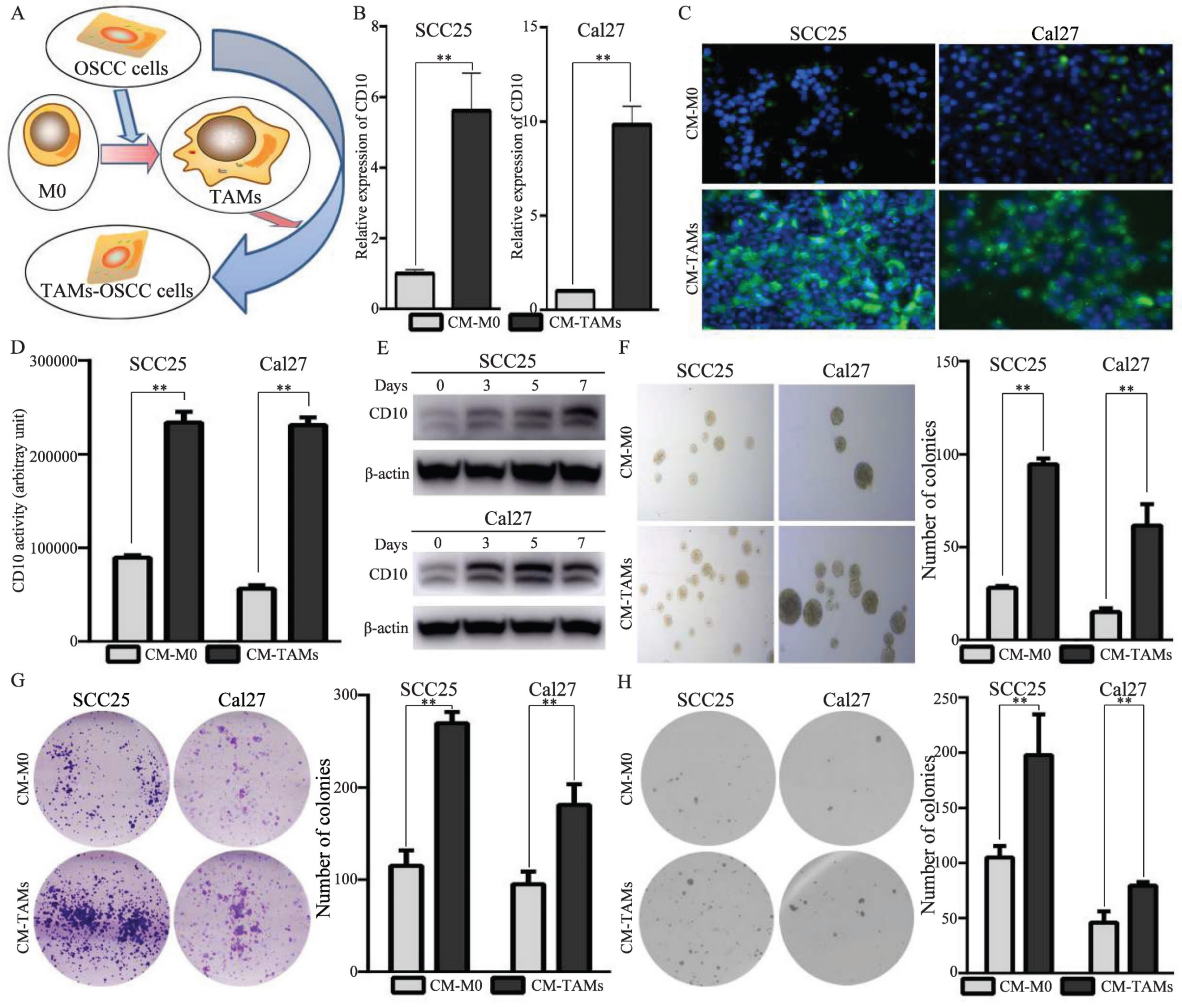
TAMs enhanced CD10 expression and CSCs behaviors in CD10^Low^ OSCC cells. (A) Illustration of the OSCC -TAMs-CSCs feedback loop; (B-E) Effects of TAMs-CM on CD10 expression in SCC25 and Cal27 cells, as detected by qPCR (B), immunofluorescence (C), CD10 activity (D), and WB (E), **p<0.01; (F-H) Effects of TAMs-CM on microsphere formation (F), colony formation (G), and soft agar colony formation (H) potential in SCC25 and Cal27 cells; the data were presented as the means ± SDs of three independent experiments, **p<0.01.

In this study, we have observed that increased expression of CD10 in cancer cells are tightly associated with the infiltration of TAMs in OSCC. Herein, we further investigated the underlying mechanisms for the regulations of CD10*^High^* CSCs by the infiltrated TAMs. When we co-cultured the OSCC cells with low CD10 expression (SCC25 and Cal27 cells) with conditioned medium from TAMs (Fig. 4A), the expression levels of CD10 were significantly increased (Fig. 4B-C). In addition, the CD10 activity and the percentages of CD10-positive cells were also significantly increased under the treatment of CM from TAMs (Fig. 4D, Supplementary File 9). We also observed that the protein levels of CD10 were induced by CM from TAMs in a time dependent manner (Fig. 4E). What's more, CM from TAMs could obviously increase the CSC behaviors of OSCC cells (Fig. 4F-H). Taken together, the above information indicated that TAMs could drive the CSC properties of OSCC cells by increased the expression of CD10.

### 3.5 TAMs drove CD10^High^ CSCs via the IL6/STAT3 pathway in OSCC

**Figure 5:**
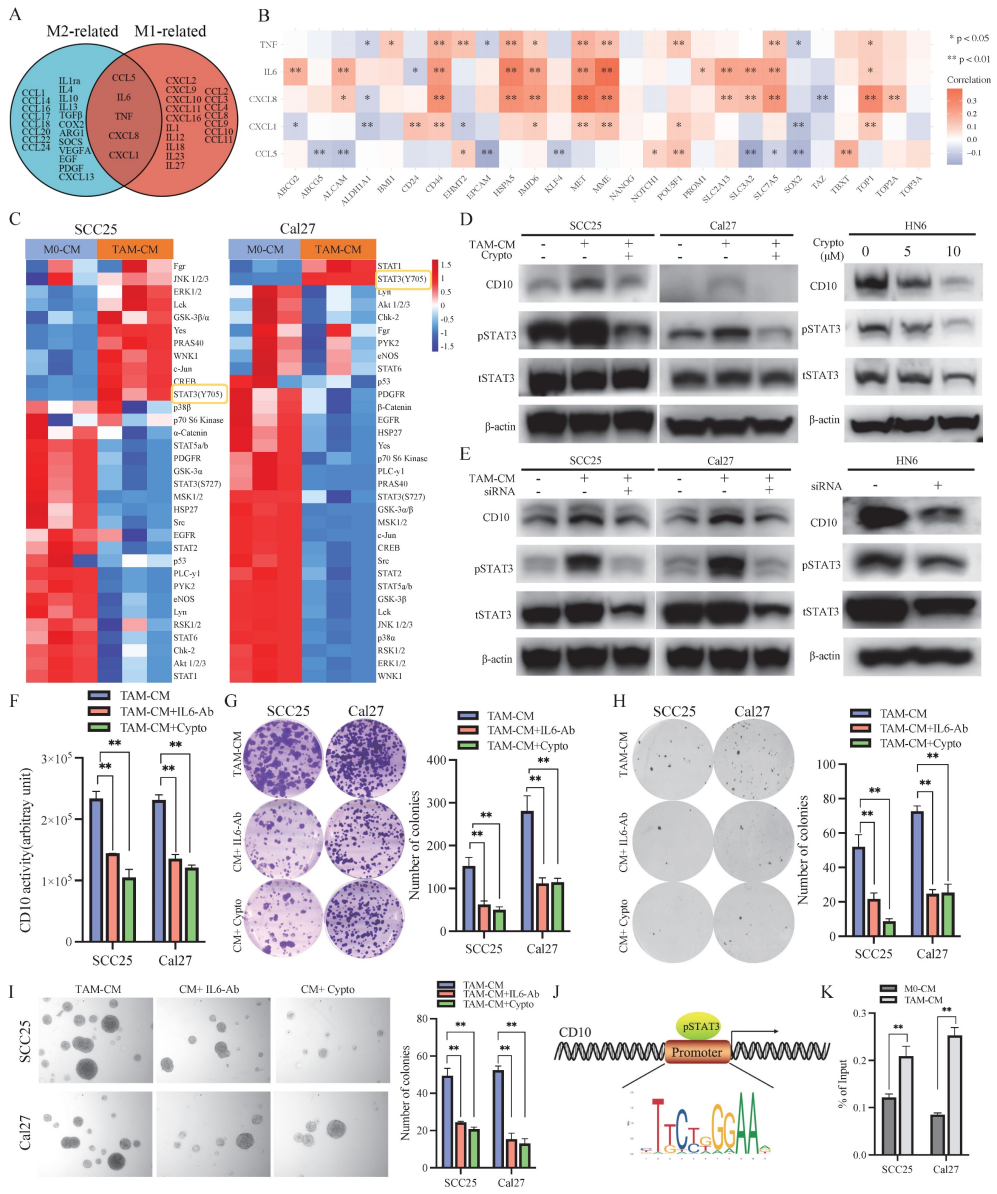
TAMs regulated the CSCs behaviors of OSCC cells via the IL6/STAT3/CD10 pathway. (A) Venn diagram depicting the cytokines shared by M1 and M2 macrophages; (B) Relationships between shared cytokines and OSCC stemness markers based on the TCGA primary HNSCC cohorts; (C) SCC25 and Cal27 cells were lysed after treatment with TAM-CM and M0-CM for 6 h, and the activation status of cellular signaling was detected by using a Human Phospho-kinase Array Kit; (D) The protein levels of CD10 and p-STAT3 were determined in SCC25 and Cal27 cells treated with TAM-CM and/or a STAT3 inhibitor and in HN6 cells treated with a STAT3 inhibitor in a dose-dependent manner by Western blot; (E) The protein levels of CD10 and p-STAT3 were determined in SCC25 and Cal27 cells treated with TAM-CM or siSTAT3 and in HN6 cells transfected with siSTAT3 by Western blot; (F) IL6-Ab and the STAT3 inhibitor reduced the effect of TAM-CM on CD10 activity; **p<0.01; (G-I) IL6-Ab and the STAT3 inhibitor reversed the CSCs behaviors induced by TAM-CM on the colony formation (G), soft agar colony formation (H), and microsphere formation (I) potentials of OSCC cells. The data were presented as the mean ± SD of three independent experiments; **p<0.01; (J) The STAT3-binding motif was predicted by JASPAR, and schematic images of the potential STAT3 binding sites in the CD10 promoter region were shown. (K) ChIP-qRCR analysis of STAT3 binding on CD10 promoter in OSCC cells treated with CM from M0-like and M1-like TAMs for 30 minutes. **p<0.01.

Subsequently, we investigated the underlying mechanism by which TAMs drove CD10^High^ CSCs in OSCC. Our previous study revealed that TAMs related molecules (M1-like TAMs and M2like TAMs) were associated with CD10 expression in OSCC (Fig. 1). Venn analysis indicated that TNF-α, IL6, CXCL8, CXCL1, and CCL5 were shared between M1-like TAMs and M2-like TAMs (Fig. 5A). Further bioinformatic analysis indicated that the expression of IL6 was strong correlated with the expression of CD10 (Fig. 5B). We found that the secretion of IL6 of TAMs was sharply increased under the education of OSCC cells (Supplementary File 10). Activation of intracellular signaling pathways was detected in OSCC cells after treatment with CM from TAMs. An increased expression of p-STAT3 (Y705) was observed in SCC25 and Cal27 cells after treatment with CM from TAMs (Fig. 5C). When blocking STAT3 signaling, the increase level of CD10 expression was significantly inhibited for the OSCC cells treated with CM-TAMs (Supplementary File 11; Fig. 5D-E). Besides, IL6-Ab or cryptotanshinone could significantly decrease the increased CD10 activity of OSCC cells treated with CM from TAMs (Fig. 5F). Subsequently, we observed that silencing IL-6 or STAT3 remarkably impaired the CSCs properties of OSCC cells induced by CM-TAMs (Fig. 5G-I). Accordingly, the binding sites of STAT3 were predicted in the CD10 promoter (Fig. 5J). Then, ChIP-qPCR was performed to confirm the binding of STAT3 to the CD10 promoter in SCC25 and Cal27 cells, and CM from TAMs could effectively promote the enrichment of pSTAT3 in the promoter region of CD10 (Fig. 5K).

**Figure 6:**
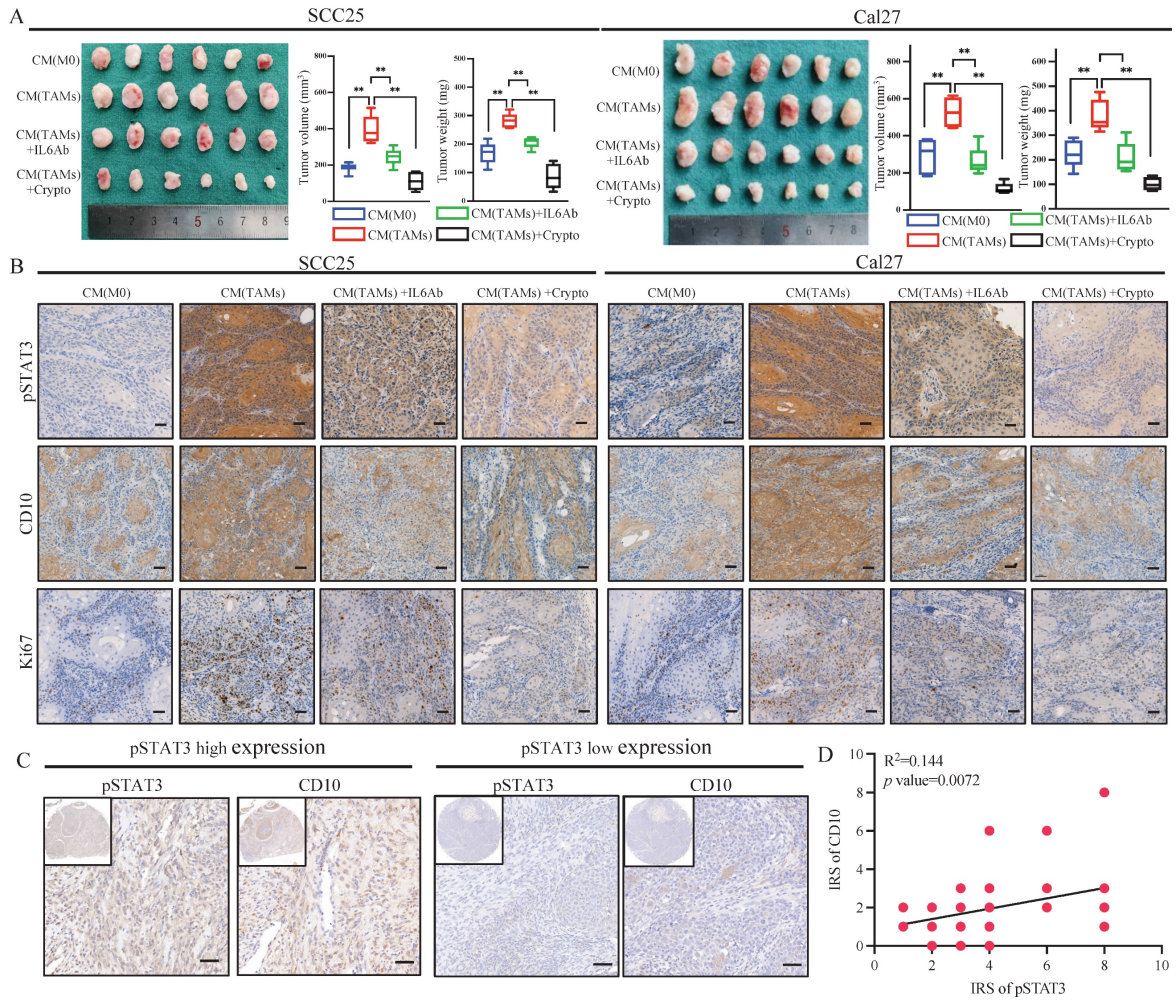
IL6-Ab and STAT3 inhibitors downregulated CD10 expression and reversed CSCs behaviors induced by TAM-CM *in vivo*. (A) SCC25 and Cal27 cells were subcutaneously injected into BALB/c-nu mice. When tumors became palpable, the mice were treated with TAM-CM (100 μL, i.t.), cryptotanshinone (50 mg/kg, i.p.), or IL6-Ab (50 μg/mL, i.t.) daily, and tumor sizes and the tumor weight were measured. **p<0.01; (B) IHC staining of pSTAT3, CD10, and Ki-67 in xenograft tumors, scale bar=50μm; (C) Representative IHC images showing the correlation between pSTAT3 activation and CD10 expression in the primary OSCC cohort; (D) Immunoreactivity scores (IRSs) were calculated for primary OSCC TMAs (n=48, 200×, *p*<0.01).

Subsequently, further *in vivo* assays indicated that CM from TAMs could increase the tumor growth of OSCC cells, and IL6-Ab or cryptotanshinone could reverse these effects (Fig. 6A). Immunohistochemically, increased expression of p-STAT3, CD10, and Ki67 was observed for the tumors treated with CM from TAMs, which could be inhibited by treatment with IL6-Ab or cryptotanshinone (Fig. 6B, Supplementary File 12). In the validated OSCC cohort, we also observed that increased expression of CD10 in the samples with increased activation of pSTAT3 (Fig. 6C-D). Hence, the above data demonstrated the efficacy of the TAMs for regulating CD10^High^ CSCs via the IL6/STAT3/CD10 pathway in OSCC.

### 3.6 CD10^High^ CSCs recruited and reprogramed tumor-associated neutrophils by secreting S100A8/A9 in OSCC

**Figure 7:**
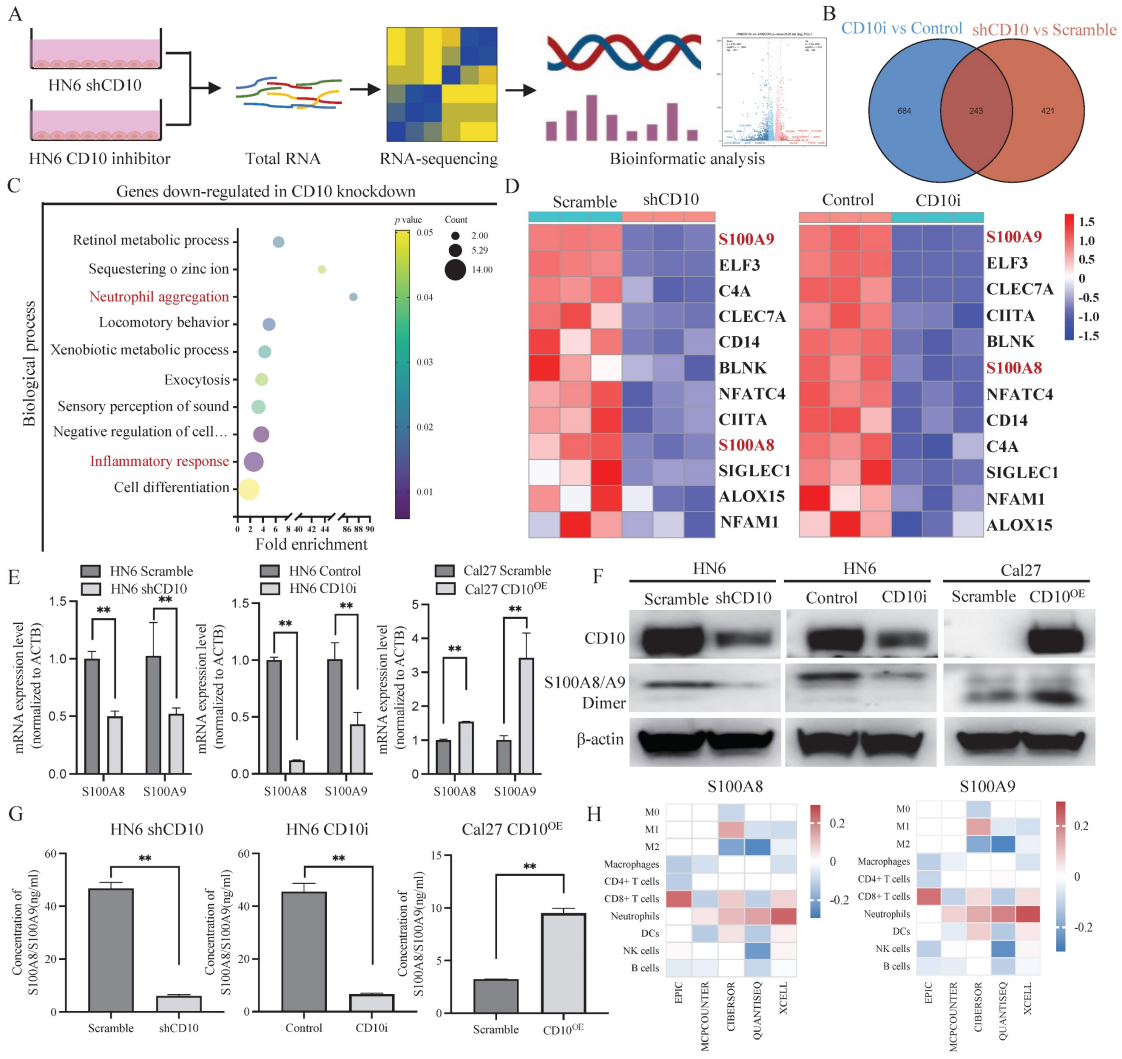
CD10^High^ CSCs secreted S100A8/A9 in OSCC. (A) Workflow of the RNA-sequencing experiment; (B) Venn diagram showing genes shared by both HN6-shCD10 and HN6-CD10i cells; (C) Functional annotation clustering of genes regulated by CD10 in HN6 cells was shown. The 10 most enriched groups were ranked based on *p*-values; (D) Heatmap showed the expression changes for genes associated with the “inflammatory response” regulated by CD10 in HN6 cells; (E-G) The expression of S100A8/A9 in HN6-shCD10, HN6-CD10i and Cal27-CD10^OE^ cells was detected by qPCR, WB and ELISA, **p<0.01; (H) S100A8/A9 expression was associated with neutrophil infiltration in the TCGA database based on five deconvolutional algorithms.

The molecular profiles of CD10^High^ CSCs after CD10 inhibition were analyzed by RNA-sequencing (Fig. 7A). A Venn diagram showed that 243 genes were shared in CD10-shRNA and CD10-inhibitor groups (Fig. 7B). Gene Ontology (GO) analysis revealed that the genes downregulated with CD10 knockdown were mainly associated with the biological processes including inflammatory responses and neutrophil aggregation (Fig. 7C). Thereinto, we found that S100A8 and S100A9 had the highest enrichment scores (Fig. 7C-D). To explore the effects of CD10 on S100A8/A9, we detected the changes in the mRNA and protein expression of S100A8/A9 in CD10 inhibitor or CD10-overexpressing OSCC cells. Besides, we observed a significant positive correlation between CD10 and S100A8/A9 expressions (Fig. 7E-G). Furthermore, immune infiltration analysis from five algorithms revealed that S100A8/A9 was associated with neutrophils based on the TCGA-HNSCC cohort (Fig. 7H). More importantly, CD10 expression was also significantly correlated with neutrophil infiltration (Fig. 1A). These results indicated that CD10^High^ CSCs might regulate neutrophils aggregation via secreting S100A8/A9.

**Figure 8:**
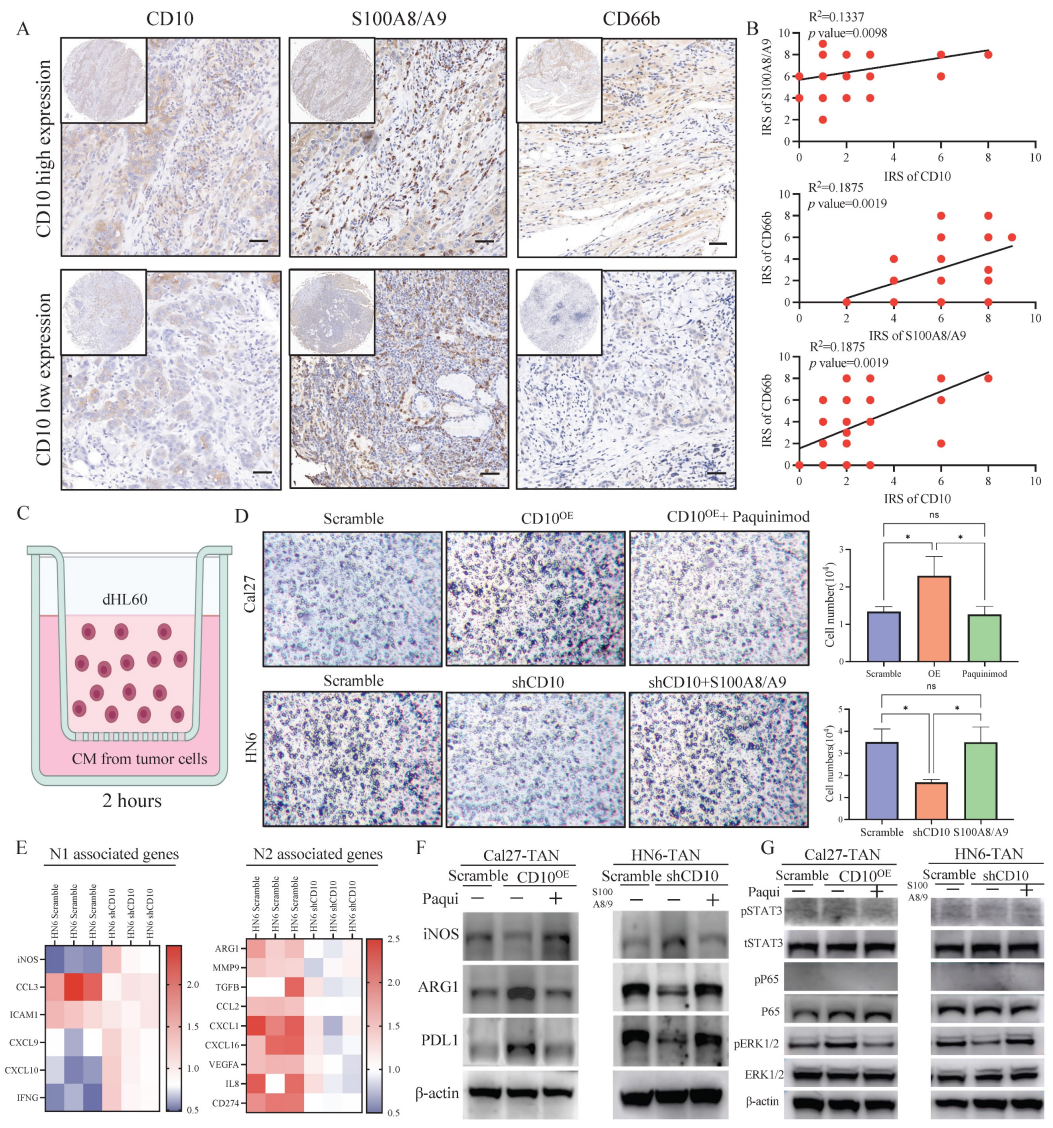
CD10^High^ CSCs recruited and reprogrammed tumor-associated neutrophils. (A) Representative IHC images of CD10, S100A8/A9, and CD66b in the primary OSCC cohort (200×), scale bar=50μm; (B) Correlations between CD10, S100A8/A9, and CD66b expression were analyzed in the OSCC cohort (n=48); (C) Diagram indicated the the *in vitro* neutrophil chemotaxis assay using differentiated HL60 neutrophils incubated with CM from OSCC cells with CD10 knockdown or overexpression; (D) Representative images of migrated dHL60 neutrophil cells educated with CM from CD10-overexpression Cal27 cells with the S100A8/A9 inhibitor Paquinimod or CD10-knockdown HN6 cells transfected with exogenous S100A8/A9, *p<0.0 5; (E) Relative expression of N1-and N2-associated genes in TANs incubated with CM from HN6-scrambled and HN6-shCD10 cells; (F) The protein levels of iNOS, ARG1, and PDL1 were detected in dHL60 neutrophil cells treated with CM from CD10-overexpression Cal27 cells with S100A8/A9 inhibitor Paquinimod or CM from CD10-knockdown HN6 cells transfected with exogenous S100A8/A9; (G) The protein levels of p-STAT3, STAT3, p-ERK, ERK, p-p65 and p65 were detected in dHL60 neutrophil cells treated with CM from CD10-overexpression Cal27 cells with S100A8/A9 inhibitor Paquinimod or CM from CD10-knockdown HN6 cells transfected with exogenous S100A8/A9.

To explore the effects of CD10 on neutrophil, we firstly detected the relationships among CD10, S100A8/A9 and CD66b in OSCC. IHC staining analysis showed CD10, S100A8/A9, and CD66b in the primary OSCC cohort (n = 48), and significantly positive relationships were observed between CD10, S100A8/A9, and CD66b (Fig. 8A-B). To further investigate the underlying regulatory effects of CD10^High^ CSCs on neutrophils infiltration, we performed an *in vitro* chemotaxis assay using differentiated HL60 (dHL60) cells (Fig. 8C). HL60 is a commonly used substitute cell line model to study neutrophil phenotypic functions that can differentiate into neutrophil-like cells after DMSO treatment (Supplementary File 13). We found that inhibiting S100A8/A9 remarkably impaired the migration abilities of dHL60 cells induced by CM from Cal27-CD10^OE^ cells. Exogeneous S100A8/A9 expression could restore the migration abilities of dHL60 cells reduced by CM from HN6 cells with CD10 knockdown (Fig. 8D).

In order to further explore the functional relationship between S100A8/A9 and neutrophils, we initially examined the correlations of S100A8/A9 with N1 and N2 respectively. We found that S100A8/A9 was positively correlated with ARG1 (a representative N2 marker) but negatively correlated with iNOS (a representative N1 marker) according to TCGA database (Supplementary File 14). We found that the gene expression patterns of the N1 and N2-related genes in the CD10 knockdown OSCC cell educated dHL60 cells had changed (Fig. 8E). The expression of N1-related genes (iNOS and CCL3) was upregulated, whereas that of the N2-associated genes (ARG1 and CXCL1) was downregulated in the CD10 knockdown OSCC cell educated dHL60 cells. Furthermore, we demonstrated that CD10^High^-CSCs regulated the expression of ARG1 and PDL1 via S100A8/A9 in dHL60 (Fig. 8F). Interestingly, we found that pERK1/2 was regulated in dHL60 recruit by S100A8/A9, indicated that CD10-positive OSCC cells might recruit and reprogram TANs via the S100A8/A9/ERK pathway (Fig. 8G). These findings indicated that TAMs drive CD10^High^ OSCC cells, which might further recruit and reprogram TANs.

### 3.7 The immunosuppressive function of TANs induced by CD10^High^ CSCs

**Figure 9:**
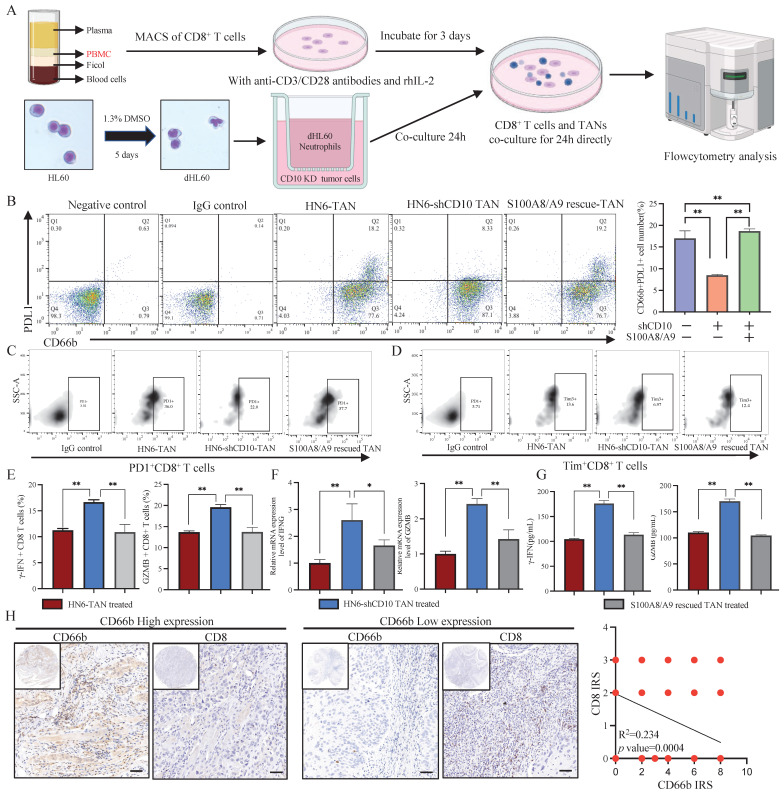
CD10^High^ CSCs induced immunosuppressive TANs. (A) Experimental workflow of CD8^+^ T cells and TANs co-culture; (B) Flow cytometry analysis for the frequency of PDL1^+^ neutrophil population in neutrophils coculture with CD10-knockdown OSCC cells transfected with exogenous S100A8/A9; The data were presented as the mean ± SD of three independent experiments, **p<0.01; (C-D) Flow cytometry analysis of the frequency of PD1^+^CD8^+^T-cell population (C) and Tim3^+^ CD8^+^T-cell population (D) in neutrophils coculture with CD10-knockdown OSCC cells transfected with exogenous S100A8/A9. The data were presented as the means ± SDs of three independent experiments. *p<0.05, **p<0.01; (E-G) The frequency of granzyme B CD8+T-cell population and γ-IFN CD8^+^ T-cell population (E), the mRNA expression levels of GZMB and IFNG (F), and intracellular granzyme B and γ-IFN levels (G), in the medium of CD8^+^T cells activated by CD3/CD28 mAbs after cocultured with CD10-knockdown OSCC cells with/without exogenous S100A8/A9; *p<0.05, **p<0.01; (H) Representative IHC images of CD66b and CD8 in OSCC samples (200×), scale bar=50μm. The correlation of the IRS score between CD66b and CD8 was analyzed in the primary OSCC cohort (n=48).

To verify the immunosuppressive function of CD10^High^ CSCs, we co-cultured CD8^+^ T cells with TANs induced by CD10 knockdown OSCC cells (Fig. 9A). In line with the previous study findings, CD10 inhibition reduced the percentage of the PDL1^+^ neutrophil population, which could be rescued by exogenous S100A8/A9 (Fig. 9B). PD1 and Tim3, two T-cell exhaustion markers, were significantly reduced in HN6-shCD10 cells treated with TANs (Fig. 9C-D, Supplementary File 15). CD8^+^ T cells eliminate malignant cells through the exocytosis of cytotoxic proteins, such as granzyme B or γ-IFN. Therefore, we used flow cytometry to detect the effect of CD10 on granzyme B or γ-IFN. We found that intracellular granzyme B and γ-IFN levels were increased for the CD8^+^ T cells cultured with HN6-shCD10 educated TANs (Fig. 9E). Furthermore, RNA expression levels and extracellular concentrations of granzyme B and γ-IFN were also higher in the HN6-shCD10-treated group than in the control group (Fig. 9F-G). Importantly, the effect of HN6-shCD10 educated TANs on CD8^+^ T cells was rescued by exogenous S100A8/A9 (Fig. 9C-G). In addition, T cell proliferation experiment indicated that CD10 educated neutrophils could impair CD8^+^ T cells proliferation (Supplementary File 16). Accordingly, TAN infiltration was negatively associated with CD8^+^ T cell infiltration in the primary OSCC cohort (Fig. 9H). Overall, these results suggested that CD10^High^ CSCs might enhance their immunosuppressive ability via secreting S100A8/A9 proteins.

## 4. Discussion

In this study, the current data suggested that infiltrated TAMs could drive heterogenetic CD10^High^ CSCs via the IL6/STAT3/CD10 pathway in OSCC. In addition, CD10^High^ CSCs induced immunosuppressive reprogramming of TANs by secreting S100A8/A9.

Cancer stem cells (CSCs), an important subpopulation of tumor cells play pivotal roles in the initiation, progression, metastasis and relapse of cancer 2, 27. Recently, the CSCs population has been characterized by diverse subgroups that exhibit distinct phenotypic and genotypic profiles 28. Although the genetic changes could drive the onset and development of CSCs, no specific genetic events have been confirmed in OSCC 29, 30. Recently, a large number of studies have reported that TME could drive malignant cells to acquire stem-like properties (dedifferentiation) 8, 16. Previously, we have reported that TAMs in TME could regulate the stem-like properties of OSCC cells 21. In this work, we have further discovered and reported the heterogenetic CD10^High^ CSCs regulated by TAMs in OSCC.

TAMs are known to be well-entrenched in inflammation and stemness associated with tumor progression in different types of cancer 30, 31. In pancreatic ductal adenocarcinoma, TAMs were reported to regulate epithelial-mesenchymal transition (EMT) and cancer stemness through the up-regulation of LOXL2^32^. In glioma, M2-like TAMs could maintain the CSC status via integrin αvβ5-Src-Stat3 signaling 33. In OSCC, we have reported that TAMs could cascade a stem-like phenotype of OSCC via a new feedback loop 14, 21. In this study, we further focused on the association between TAMs infiltration and the regulation of CSCs heterogeneity. Previously, CD10 has been reported to be expression in cancer-associated fibroblasts, CD10^+^GPR77^+^ CAFs enhance tumor growth and resistance to chemotherapy by supporting CSCs, which is sustained by continuous NF-κB activation through p65 phosphorylation and acetylation 34. Recently, CD10 was reported as a novel CSCs marker and implicated in developing cisplatin resistance of OSCC 35, 36. However, the potential mechanisms contributing to dysregulated CD10 in HNSCC remains not clear 37. In this study, we reported for the first time that infiltrated TAMs could drive heterogenetic CD10^High^ CSCs via the IL6/STAT3/CD10 pathway. Indeed, the pharmacological blockade of CD10 has been promised to impair the CSC function of OSCC cells, but the underlying modulators of CD10-positive cells has not been clearly defined. In this study, we managed to demonstrated that TAMs could drive the CSC behaviors via up-regulating CD10 expression of OSCC cells. Besides, CD10^High^ CSCs could induce immunosuppressive reprogramming of TANs. So, targeting CD10^High^ CSCs seems to be a promising strategy to adjuvant the treatment of OSCC patients.

IL6 has been shown to be a multifunctional cytokine involved in the regulation of cancer progression and involved in the malignant processes such as EMT, angiogenesis, and treatment resistance 31. JAK/STAT3 signaling has been identified as an important downstream pathway of IL6 10. Herein, we found that abundant IL6 secreted from activated TAMs induced CSCs via the IL6/STAT3/CD10 pathway in OSCC. As is known, CSCs exhibit phenotypic, functional, and transcriptomic heterogeneity 32, 33. Various subpopulations of CSCs harbor unique molecular signatures that influence prognosis and therapeutic strategies for cancer. Escaping from immune surveillance, an important stage in tumorigenesis, is the first step for tumor initiation and progression 38, 39. CSCs with different molecular phenotypes employ different pathways and mechanisms of immune evasion 40. During the occurrence and metastasis of HNSCC, CD276^High^ CSCs localized at the invasive front could directly inhibit the infiltration of CD8^+^ T cells, thereby facilitating immune escape 18. Additionally, CSCs commonly metastasize to and thrive in the cervical lymph nodes abundant in immune cells in HNSCC. Therefore, identifying effective interventions to modulate the interaction between CSCs and TILs might help to develop potential therapeutic strategy to improve the treatment of OSCC. In this regard, we observed that CD10^High^ CSCs overexpressing S100A8/A9 were associated with TANs aggregation. S100A8/A9 heterodimer, an exosomal protein, contributes to metastasis, angiogenesis, and immunosuppression in various cancers 41. In OSCC, CD10^High^ CSCs could recruit and drive immunosuppressive reprogramming of TANs through the S100A8/A9/ERK1/2 pathway. S100A8 and S100A9 are two proteins from S100 protein family, which were proved to be participated in the regulation of inflammation and cancer microenvironment 42, 43. S100A8/A9 are mainly distributed in neutrophils and monocytes, and are involved in many pathological processes 42. S100A8/A9 often exist in the form of hetero-O dimer formation associated with cancer 43. There is no doubt that the reprogramming process could promote the immune tolerance of cancer cells and maintain a favorable microenvironment for the malignant progression of OSCC. In this study, the paracrined S100A8/A9 induced an immunosuppressive status of TANs, which could effectively inhibit the cytotoxic function of CD8^+^ T cells in OSCC.

However, there were still possible limitations in this study. The involved bioinformatic analysis were performed based on TCGA-HNSCC cohort. So, it would be better to validate the results based on the OSCC cohort with larger number of OSCC samples. Besides, the *in vivo* assays were performed with OSCC cell lines by xenografting models. It seemed to be more validated to perform the* in vivo* assays with PDX models of OSCC, which would be further conducted in our subsequent study. In conclusion, we managed to demonstrate a novel model to investigate the regulation of CSC heterogeneity in cancer. The biological events have been investigated in OSCC-TAMs- CD10^High^ CSCs-TANs, which provided potential targets for improving the treatment strategies for OSCC, especially for CD10 and S100A8/A9.

## Supplementary Material

Supplementary files.

## References

[1] Siegel RL, Miller KD, Wagle NS, Jemal A (2023). Cancer statistics, 2023. CA: A Cancer Journal for Clinicians.

[2] Almangush A, Mäkitie AA, Triantafyllou A, de Bree R, Strojan P, Rinaldo A (2020). Staging and grading of oral squamous cell carcinoma: An update. Oral oncology.

[3] Jia L, Zhang W, Wang CY (2020). BMI1 Inhibition Eliminates Residual Cancer Stem Cells after PD1 Blockade and Activates Antitumor Immunity to Prevent Metastasis and Relapse. Cell Stem Cell.

[4] Prager BC, Xie Q, Bao S, Rich JN (2019). Cancer Stem Cells: The Architects of the Tumor Ecosystem. Cell Stem Cell.

[5] Mai Y, Su J, Yang C, Xia C, Fu L (2023). The strategies to cure cancer patients by eradicating cancer stem-like cells. Molecular cancer.

[6] Liu Y, Wang H (2023). Biomarkers and targeted therapy for cancer stem cells. Trends in pharmacological sciences.

[7] Shimokawa M, Ohta Y, Nishikori S, Matano M, Takano A, Fujii M (2017). Visualization and targeting of LGR5+ human colon cancer stem cells. Nature.

[8] Bocci F, Gearhart-Serna L, Boareto M, Ribeiro M, Ben-Jacob E, Devi GR (2019). Toward understanding cancer stem cell heterogeneity in the tumor microenvironment. Proc Natl Acad Sci U S A.

[9] Liguori M, Digifico E, Vacchini A, Avigni R, Colombo FS, Borroni EM (2021). The soluble glycoprotein NMB (GPNMB) produced by macrophages induces cancer stemness and metastasis via CD44 and IL-33. Cell Mol Immunol.

[10] Zhu M, Li S, Cao X, Rashid K, Liu T, Zhu M (2023). The STAT family: Key transcription factors mediating crosstalk between cancer stem cells and tumor immune microenvironment. Seminars in Cancer Biology.

[11] Wu B, Shi X, Jiang M, Liu H (2023). Cross-talk between cancer stem cells and immune cells: potential therapeutic targets in the tumor immune microenvironment. Mol Cancer.

[12] Shang S, Yang C, Chen F, Xiang RS, Zhang H, Dai SY (2023). ID1 expressing macrophages support cancer cell stemness and limit CD8+ T cell infiltration in colorectal cancer. Nature communication.

[13] Nasir I, McGuinness C, Poh AR, Ernst M, Darcy PK, Britt KL (2023). Tumor macrophage functional heterogeneity can inform the development of novel cancer therapies. Trends in immunology.

[14] You Y, Tian Z, Du Z, Wu K, Xu G, Dai M (2022). M1-like tumor-associated macrophages cascade a mesenchymal/stem-like phenotype of oral squamous cell carcinoma via the IL6/Stat3/THBS1 feedback loop. J Exp Clin Cancer Res.

[15] Pisco AO, Huang S (2015). Non-genetic cancer cell plasticity and therapy-induced stemness in tumour relapse: 'What does not kill me strengthens me'. British journal of cancer.

[16] Bayik D, Lathia JD (2021). Cancer stem cell-immune cell crosstalk in tumour progression. Nat Rev Cancer.

[17] Cazet AS, Hui MN, Elsworth BL, Wu SZ, Roden D, Chan CL (2018). Targeting stromal remodeling and cancer stem cell plasticity overcomes chemoresistance in triple negative breast cancer. Nature communication.

[18] Wang C, Li Y, Jia L, Kim JK, Li J, Deng P (2021). CD276 expression enables squamous cell carcinoma stem cells to evade immune surveillance. Cell Stem Cell.

[19] Moll HP, Pranz K, Musteanu M, Grabner B, Hruschka N, Mohrherr J (2018). Afatinib restrains K-RAS-driven lung tumorigenesis. Science Translational Medicine.

[20] Ju H, Hu Z, Wei D, Huang J, Zhang X, Rui M (2021). A novel intronic circular RNA, circGNG7, inhibits head and neck squamous cell carcinoma progression by blocking the phosphorylation of heat shock protein 27 at Ser78 and Ser82. Cancer Communications.

[21] Xiao M, Zhang J, Chen W, Chen W (2018). M1-like tumor-associated macrophages activated by exosome-transferred THBS1 promote malignant migration in oral squamous cell carcinoma. J Exp Clin Cancer Res.

[22] Thiam HR, Vargas P, Carpi N, Crespo CL, Raab M, Terriac E (2016). Perinuclear Arp2/3-driven actin polymerization enables nuclear deformation to facilitate cell migration through complex environments. Nature communication.

[23] You Y, Du Z, Xu G, Tian Z, Xiao M, Wang Y (2023). Identification of Exosome-Related Genes Associated with Prognosis and Immune Infiltration Features in Head-Neck Squamous Cell Carcinoma. Biomolecules.

[24] Xiao M, Yan M, Zhang J, Xu Q, Qi S, Wang X (2017). Cancer stem-like cell related protein CD166 degrades through E3 ubiquitin ligase CHIP in head and neck cancer. Experimental cell research.

[25] Aydemir EA, Simsek E, Korcum AF, Fiskin K (2016). Endostatin and irradiation modifies the activity of ADAM10 and neprilysin in breast cancer cells. Mol Med Rep.

[26] Bao X, Shi J, Xie F, Liu Z, Yu J, Chen W (2018). Proteolytic Release of the p75NTR Intracellular Domain by ADAM10 Promotes Metastasis and Resistance to Anoikis. Cancer research.

[27] Navas LE, Carnero A (2021). NAD+ metabolism, stemness, the immune response, and cancer. Signal Transduct Target Ther.

[28] Navas LE, Blanco-Alcaina E, Suarez-Martinez E, Verdugo-Sivianes EM, Espinosa-Sanchez A, Sanchez-Diaz L (2023). NAD pool as an antitumor target against cancer stem cells in head and neck cancer. Journal of Experimental & Clinical Cancer Research.

[29] Ali J, Sabiha B, Jan HU, Haider SA, Khan AA, Ali SS (2017). Genetic etiology of oral cancer. Oral oncology.

[30] Farah CS (2021). Molecular landscape of head and neck cancer and implications for therapy. Annals of translational medicine.

[31] Yin Y, Yao S, Hu Y, Feng Y, Li M, Bian Z (2017). The Immune-microenvironment Confers Chemoresistance of Colorectal Cancer through Macrophage-Derived IL6. Clin Cancer Res.

[32] Zheng H, Pomyen Y, Hernandez MO, Li C, Livak F, Tang W (2018). Single-cell analysis reveals cancer stem cell heterogeneity in hepatocellular carcinoma. Hepatology.

[33] Zhang R, Tu J, Liu S (2022). Novel molecular regulators of breast cancer stem cell plasticity and heterogeneity. Seminars in Cancer Biology.

[34] Su S, Chen J, Yao H, Liu J, Yu S, Lao L (2018). CD10+GPR77+ Cancer-Associated Fibroblasts Promote Cancer Formation and Chemoresistance by Sustaining Cancer Stemness. Cell.

[35] Wang Y, Li Q, Xu L, Chen J, Pu Y, Wang L (2021). Cancer stemness of CD10-positive cells regulated by Hedgehog pathway promotes the resistance to cisplatin in oral squamous cell carcinoma. Oral Dis.

[36] Pu Y, Li Q, Wang Y, Xu L, Qiao Q, Guo Y (2021). pERK-mediated IL8 secretion can enhance the migration, invasion, and cisplatin resistance of CD10-positive oral cancer cells. BMC Cancer.

[37] Li Q, Wang Y, Xu L, Wang L, Guo Y, Guo C (2021). High level of CD10 expression is associated with poor overall survival in patients with head and neck cancer. Int J Oral Maxillofac Surg.

[38] Miao Y, Yang H, Levorse J, Yuan S, Polak L, Sribour M (2019). Adaptive Immune Resistance Emerges from Tumor-Initiating Stem Cells. Cell.

[39] Dianat-Moghadam H, Mahari A, Salahlou R, Khalili M, Azizi M, Sadeghzadeh H (2022). Immune evader cancer stem cells direct the perspective approaches to cancer immunotherapy. Stem Cell Research & Therapy.

[40] Li L, Jensen RA (2023). Understanding and Overcoming Immunosuppression Shaped by Cancer Stem Cells. Cancer research.

[41] Zhou X, Fang D, Liu H, Ou X, Zhang C, Zhao Z (2022). PMN-MDSCs accumulation induced by CXCL1 promotes CD8+ T cells exhaustion in gastric cancer. Cancer letters.

[42] Bai G, Yue S, You Y, Zhao J, Tian Z, Wang Y (2023). An integrated bioinformatics analysis of the S100 in head and neck squamous cell carcinoma. Transl Cancer Res.

[43] Gebhardt C, Németh J, Angel P, Hess J (2006). S100A8 and S100A9 in inflammation and cancer. Biochem Pharmacol.

