# Supplementary material for: Tumor-associated macrophages drive heterogenetic CD10High cancer stem cells to implement tumor-associated neutrophils reprogramming in oral squamous cell carcinoma
Source: Int J Biol Sci. 2025 Jan 13;21(3):1110–26. doi: 10.7150/ijbs.100611 (PMC11781160; doi:10.7150/ijbs.100611)

Case A01:

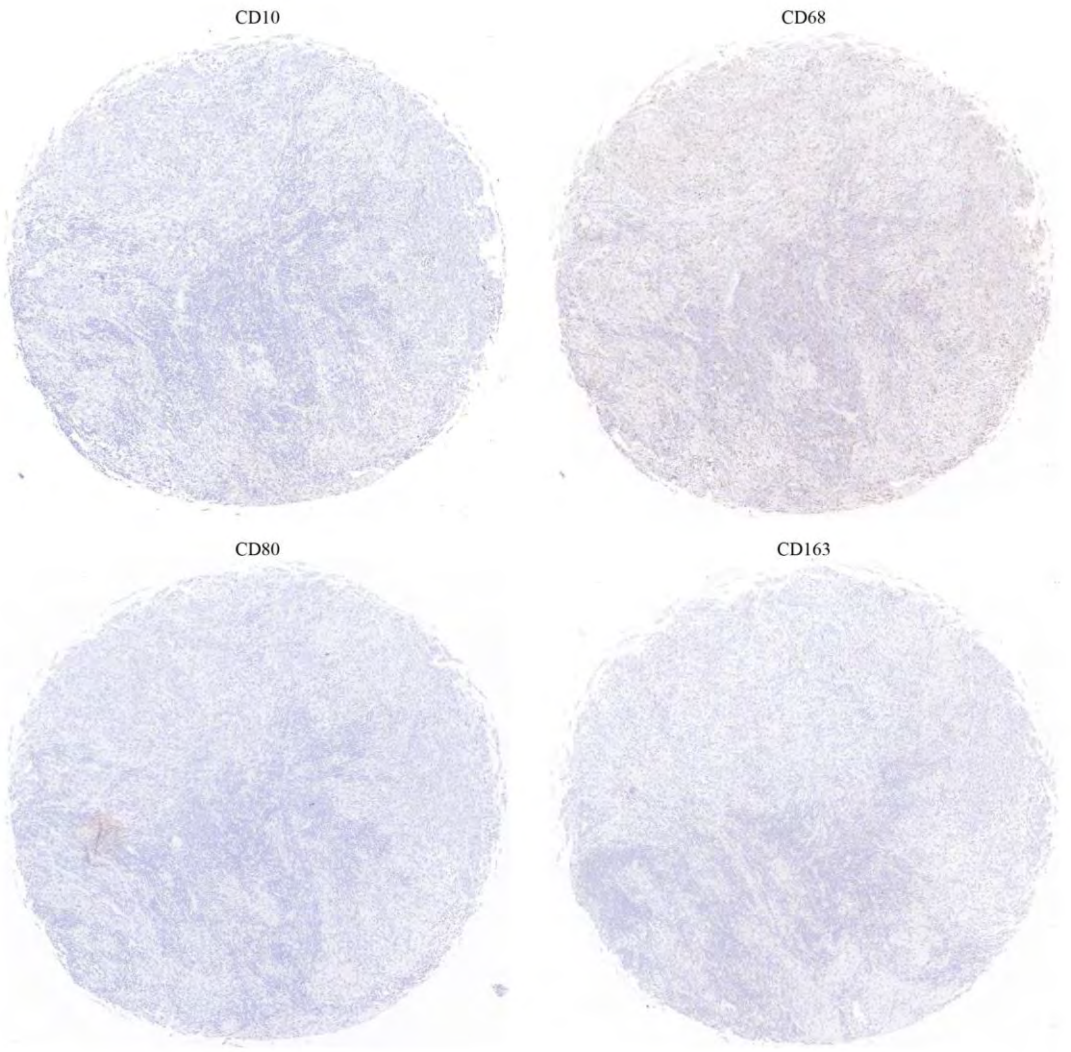

Case A03:

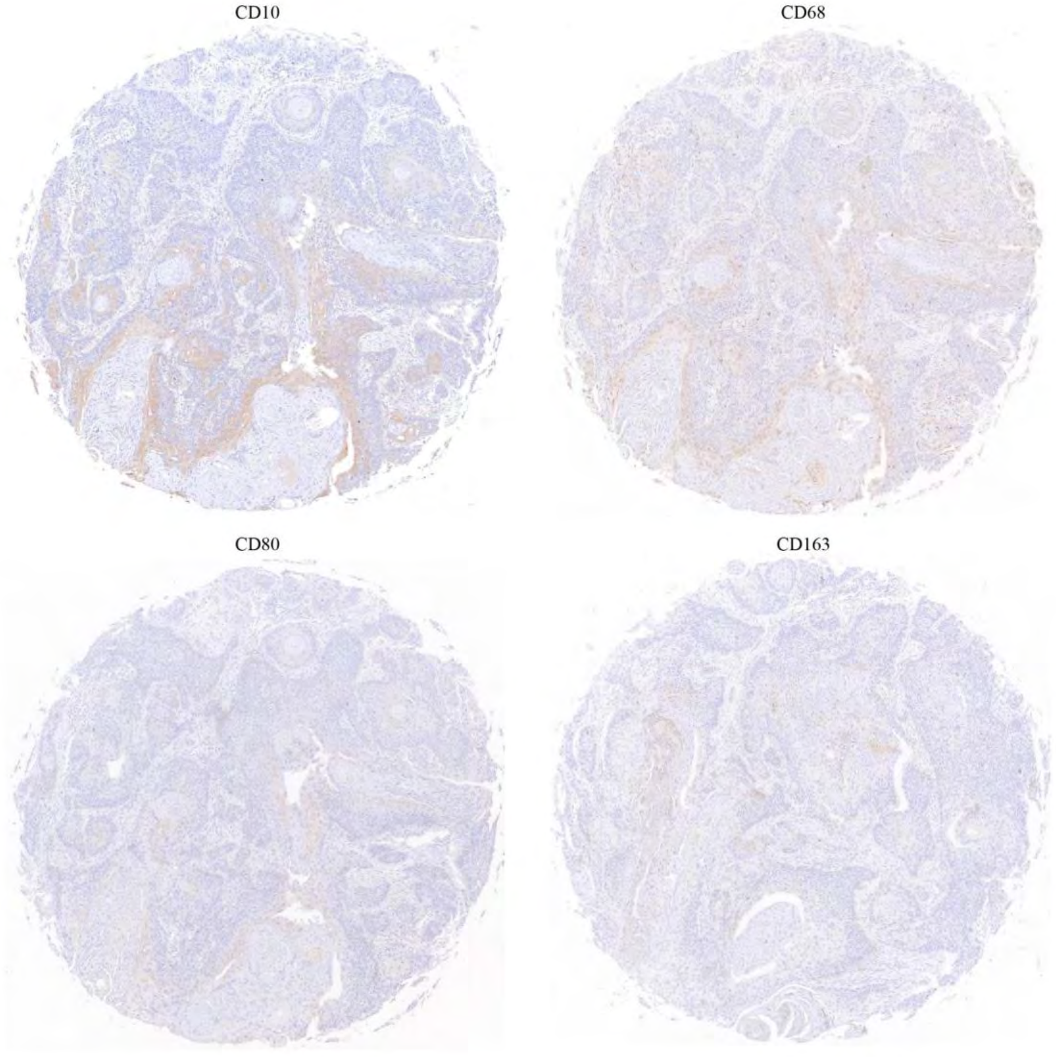

Case A05:

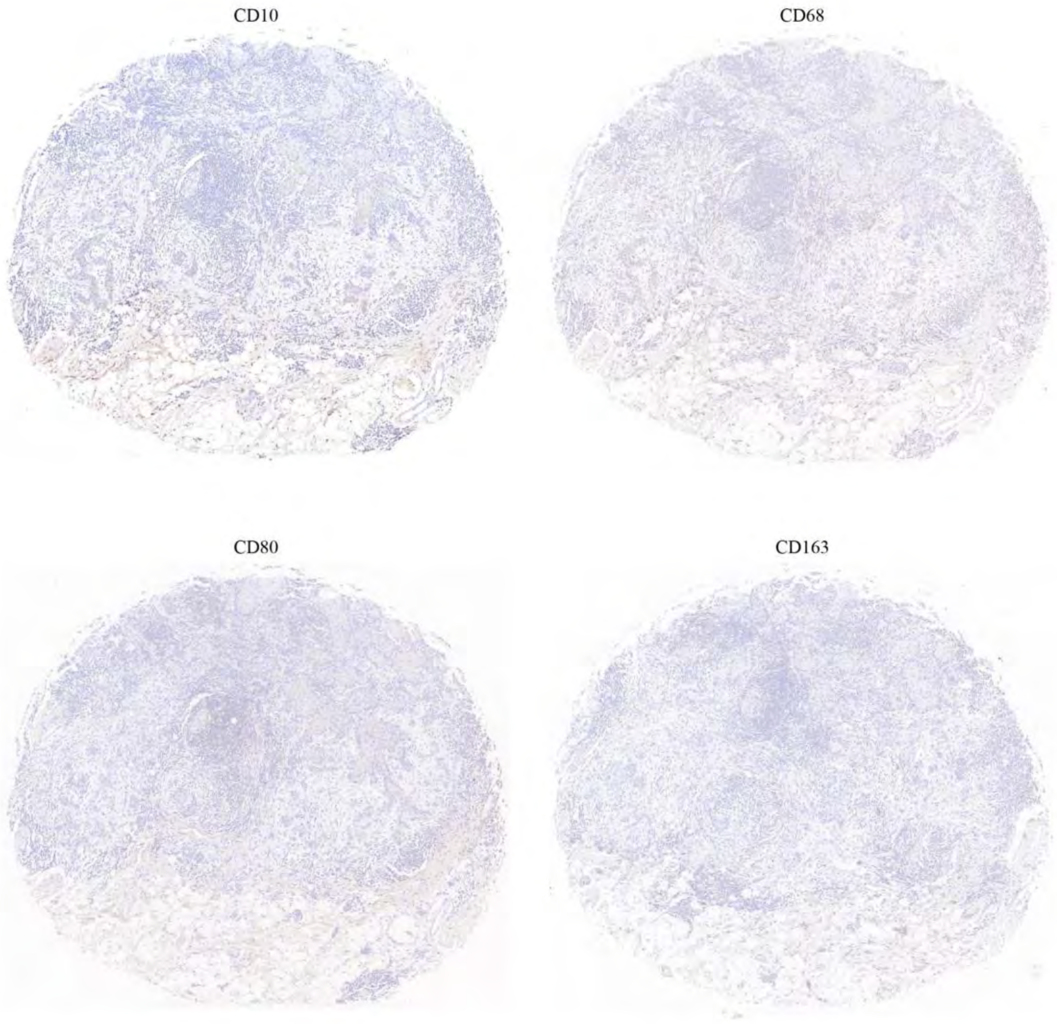

Case A07:

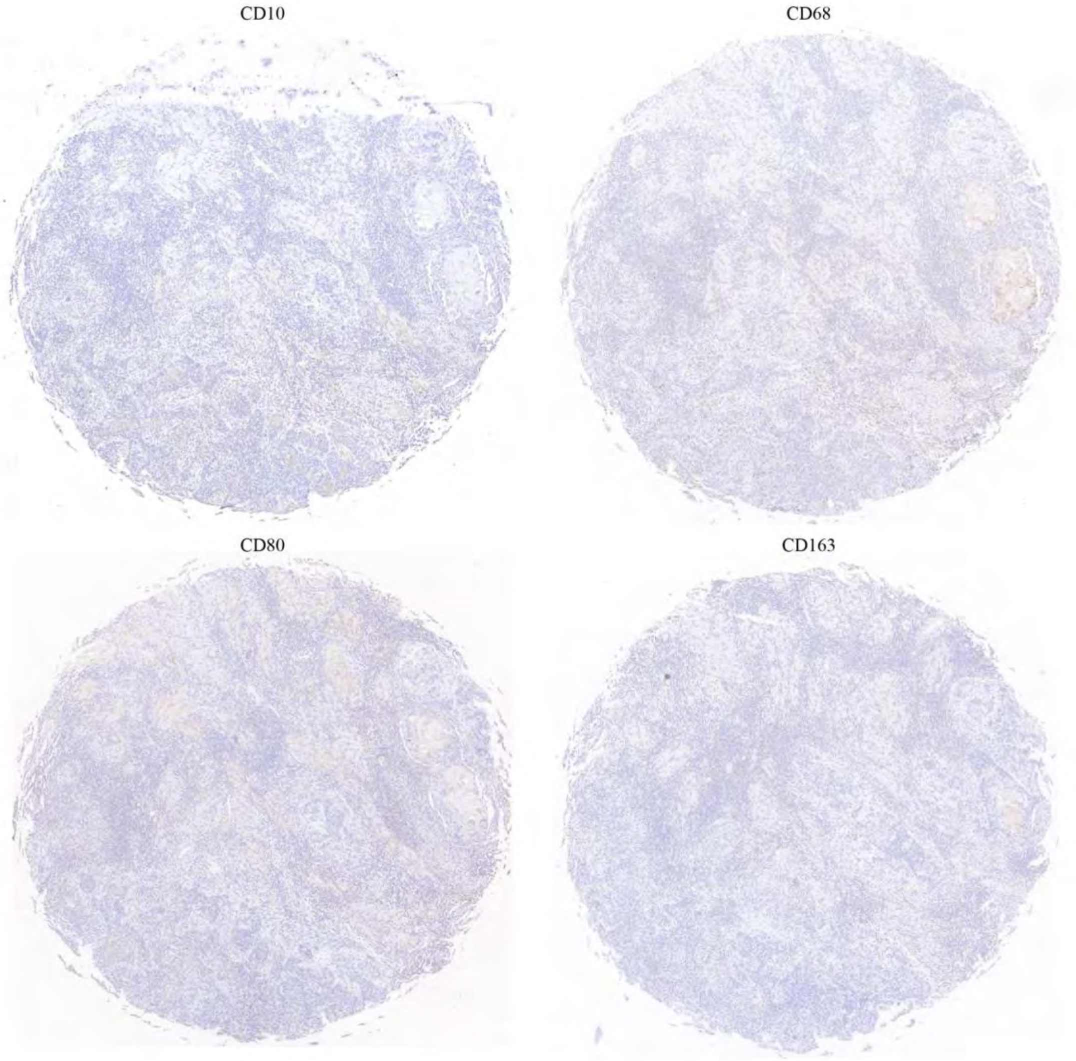

Case A09:

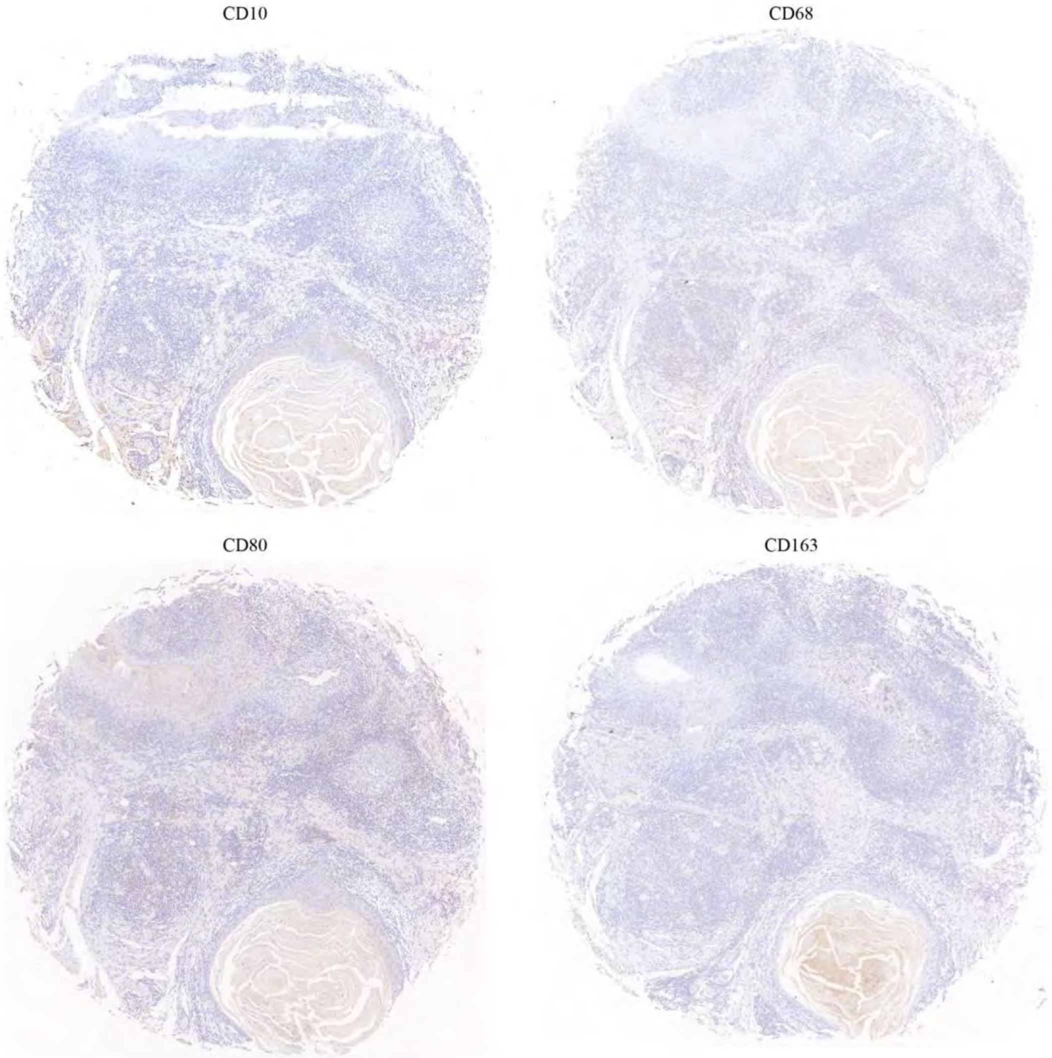

Case A11:

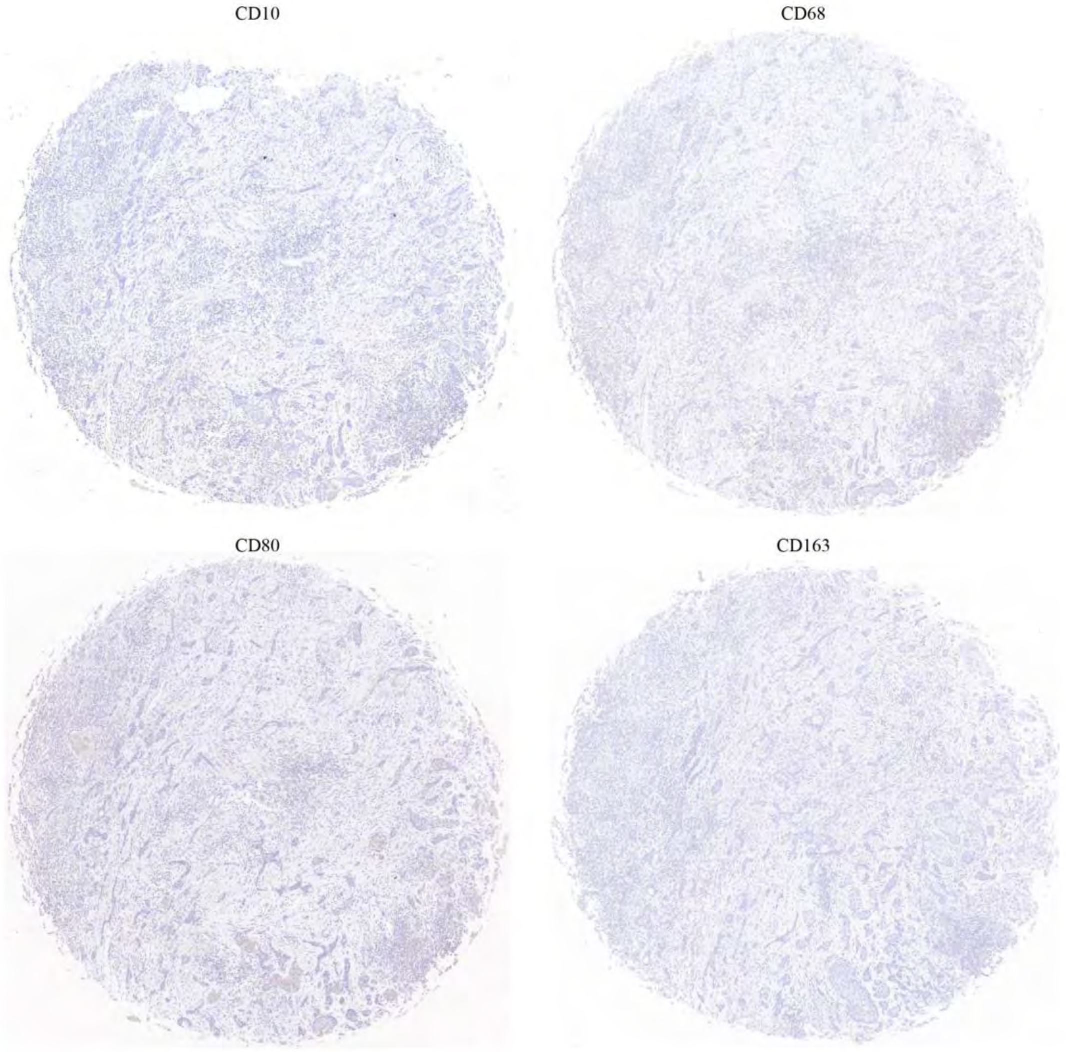

Case B01:

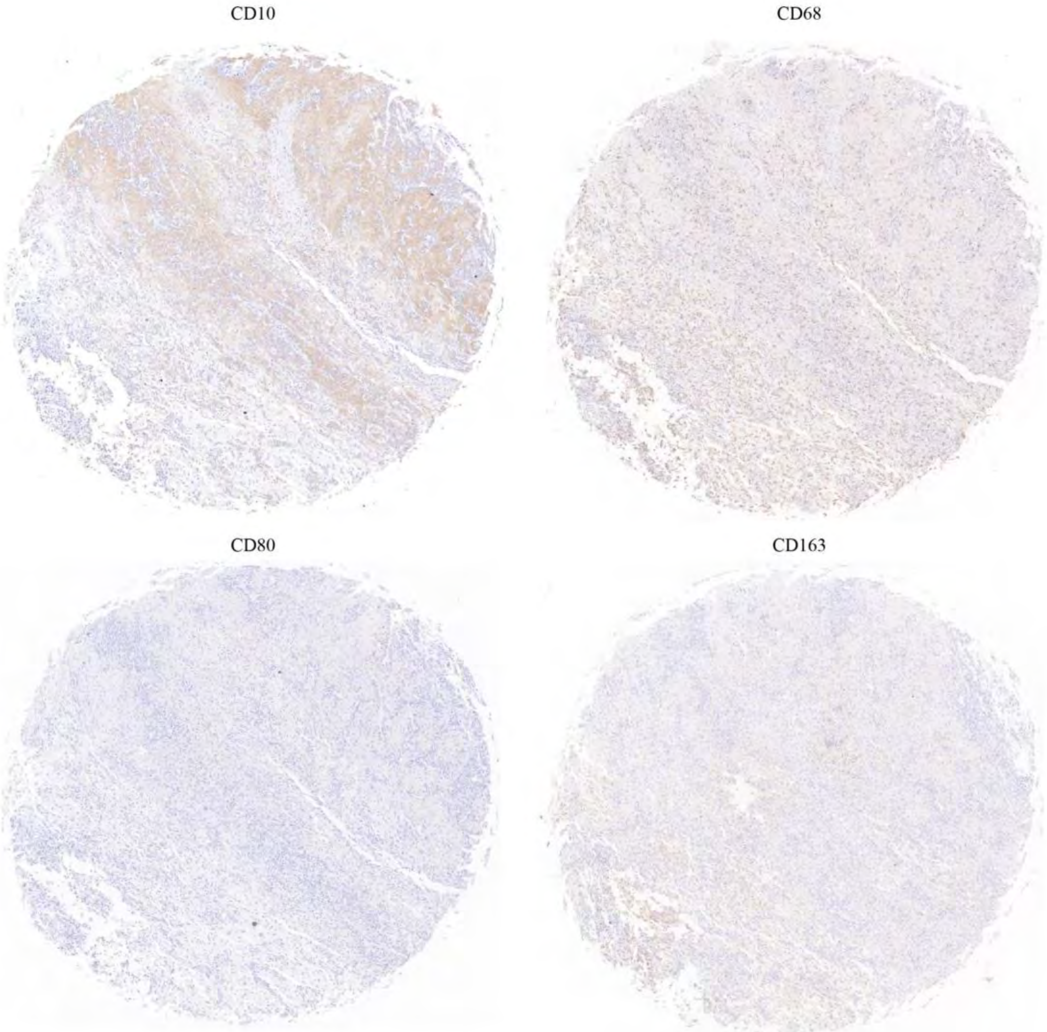

Case A03:

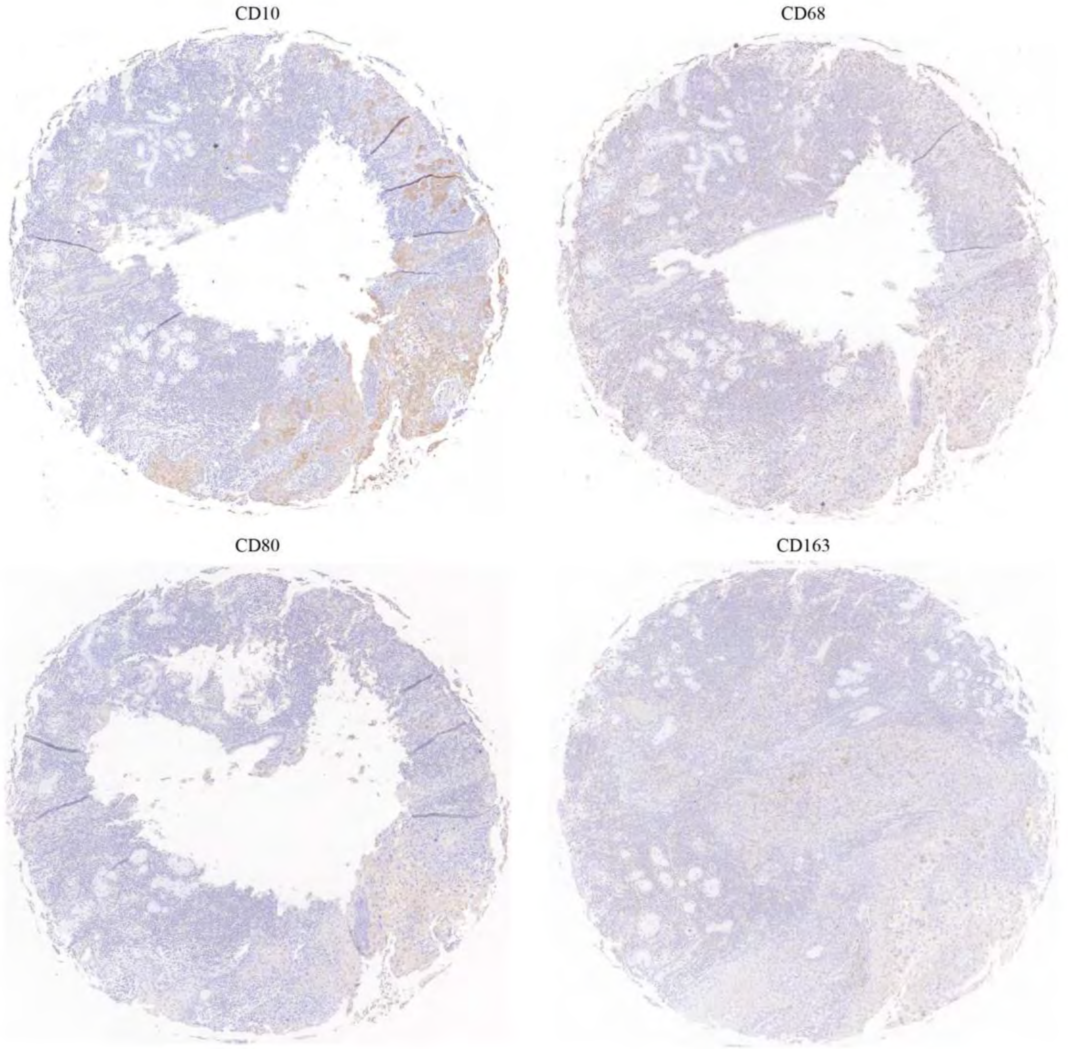

Case B05:

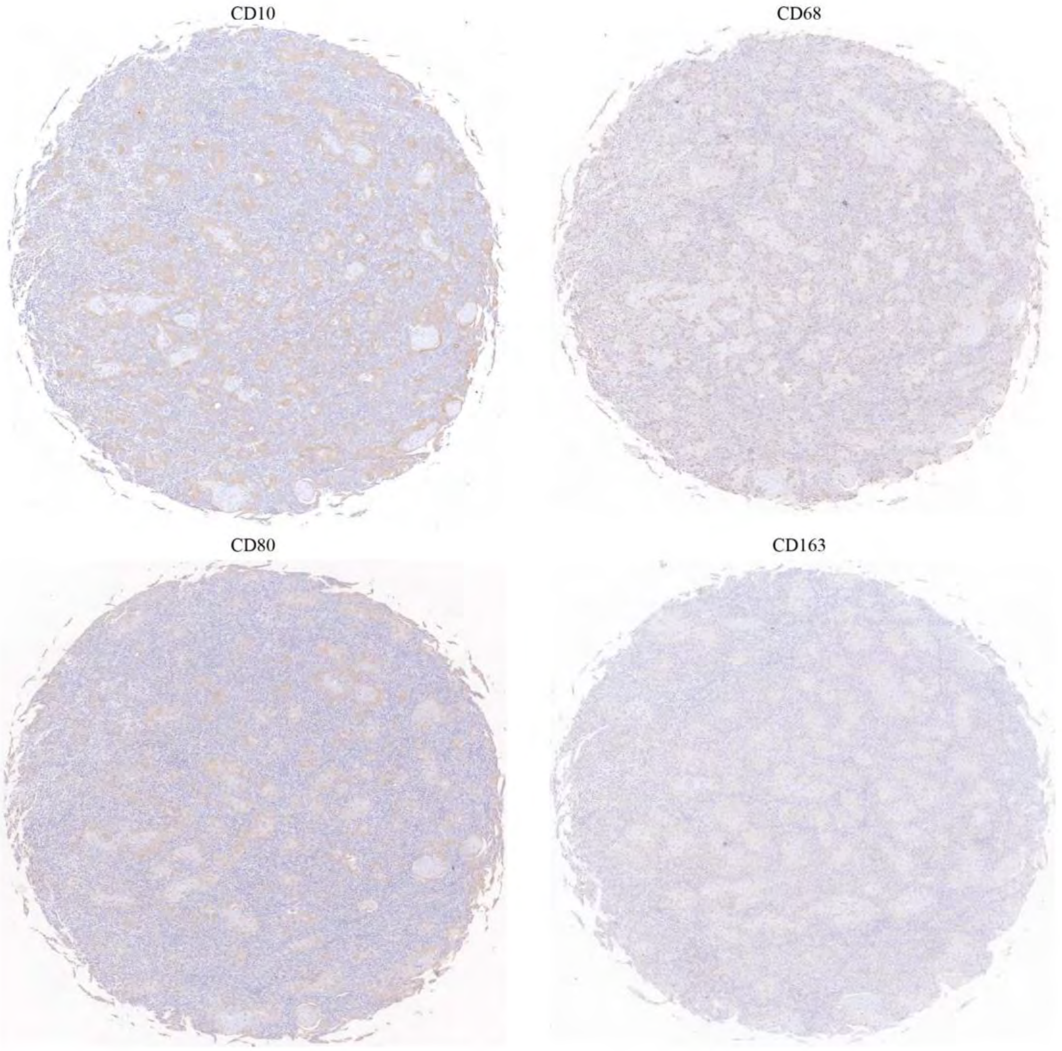

Case B07:

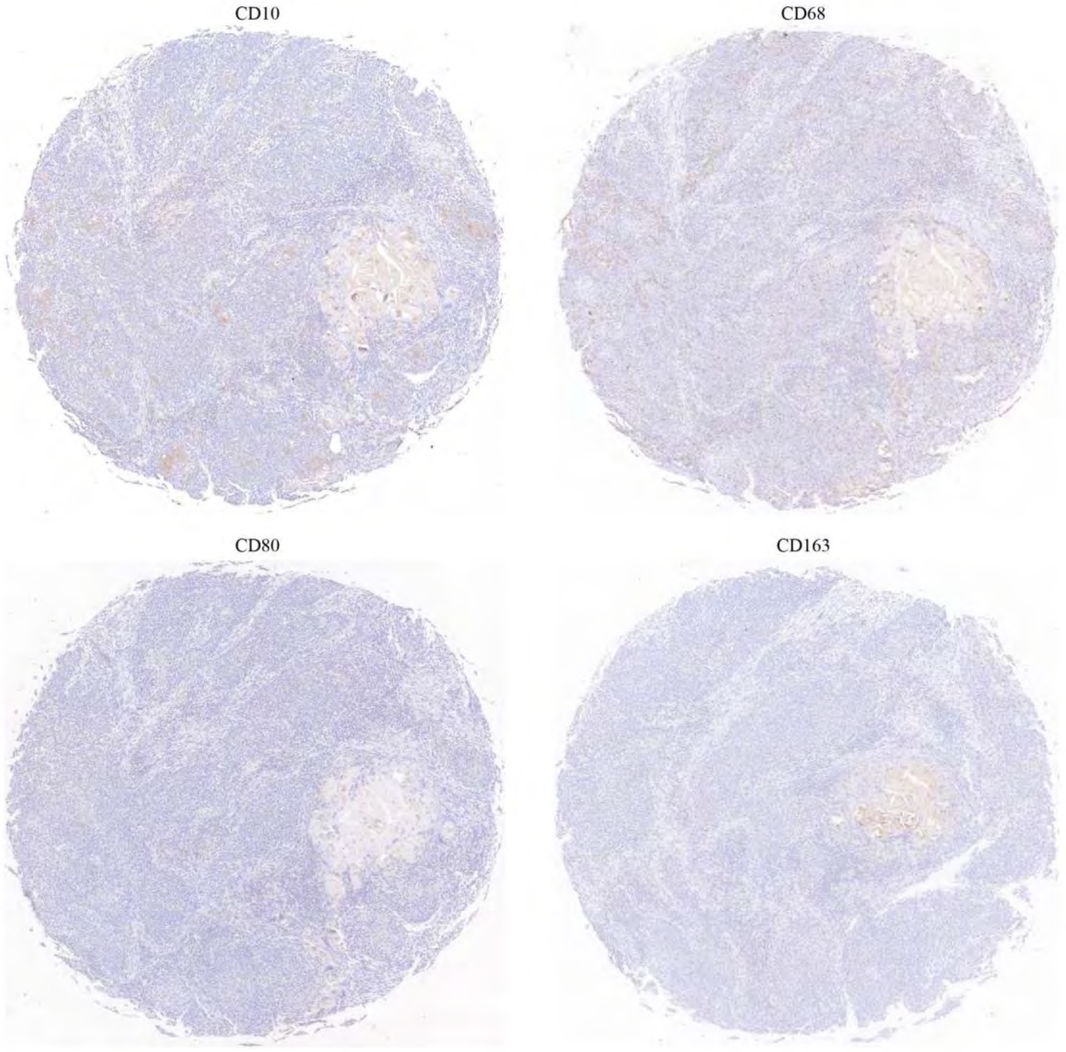

Case B09:

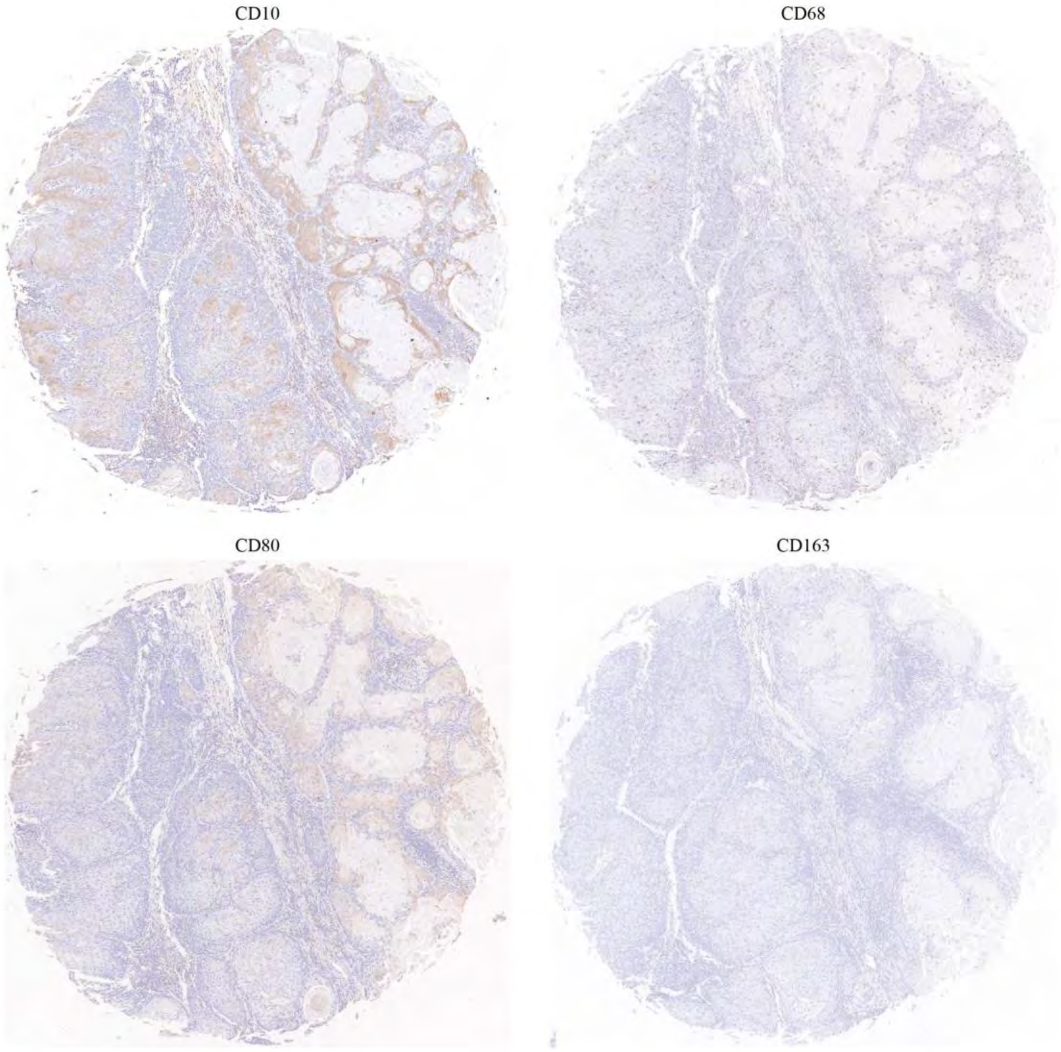

Case B11:

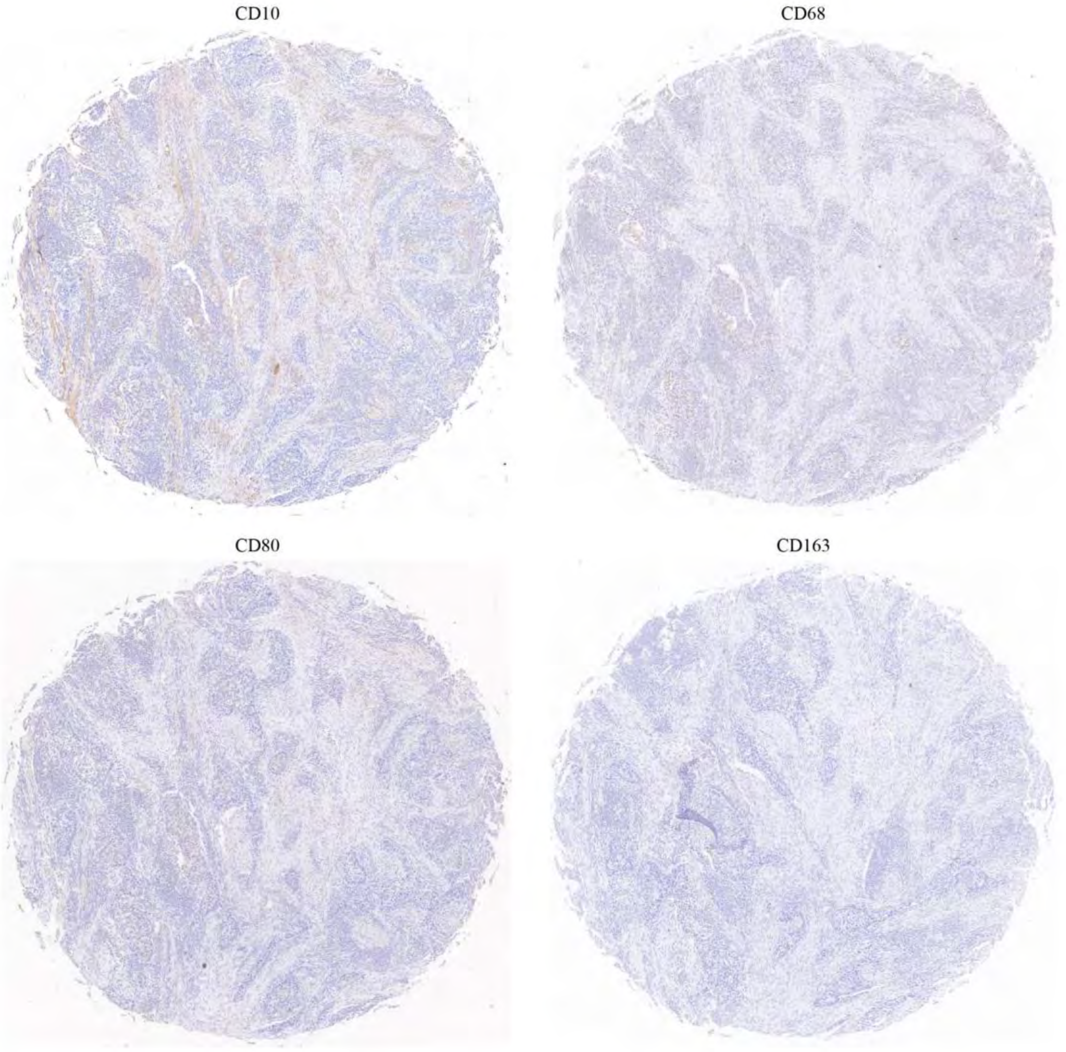

Case C01:

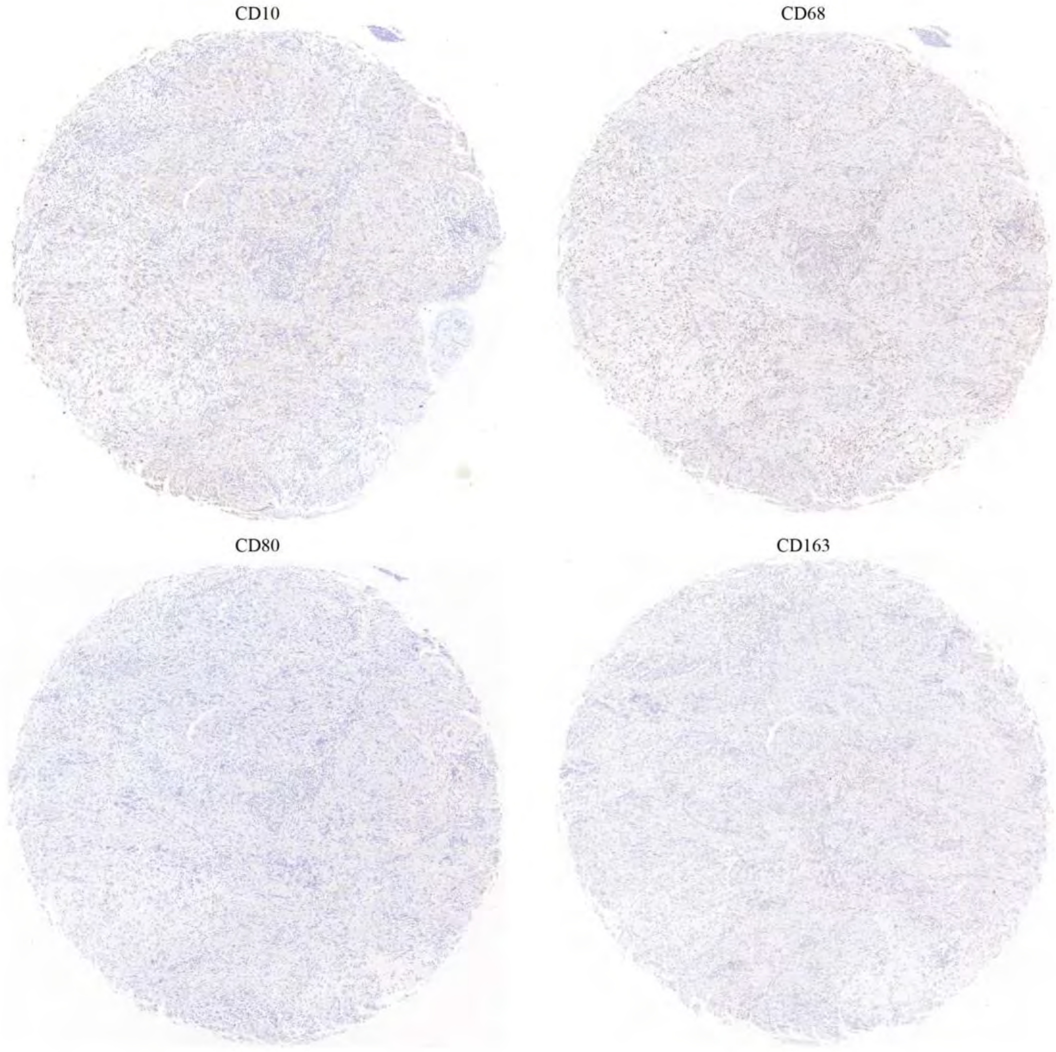

Case C03:

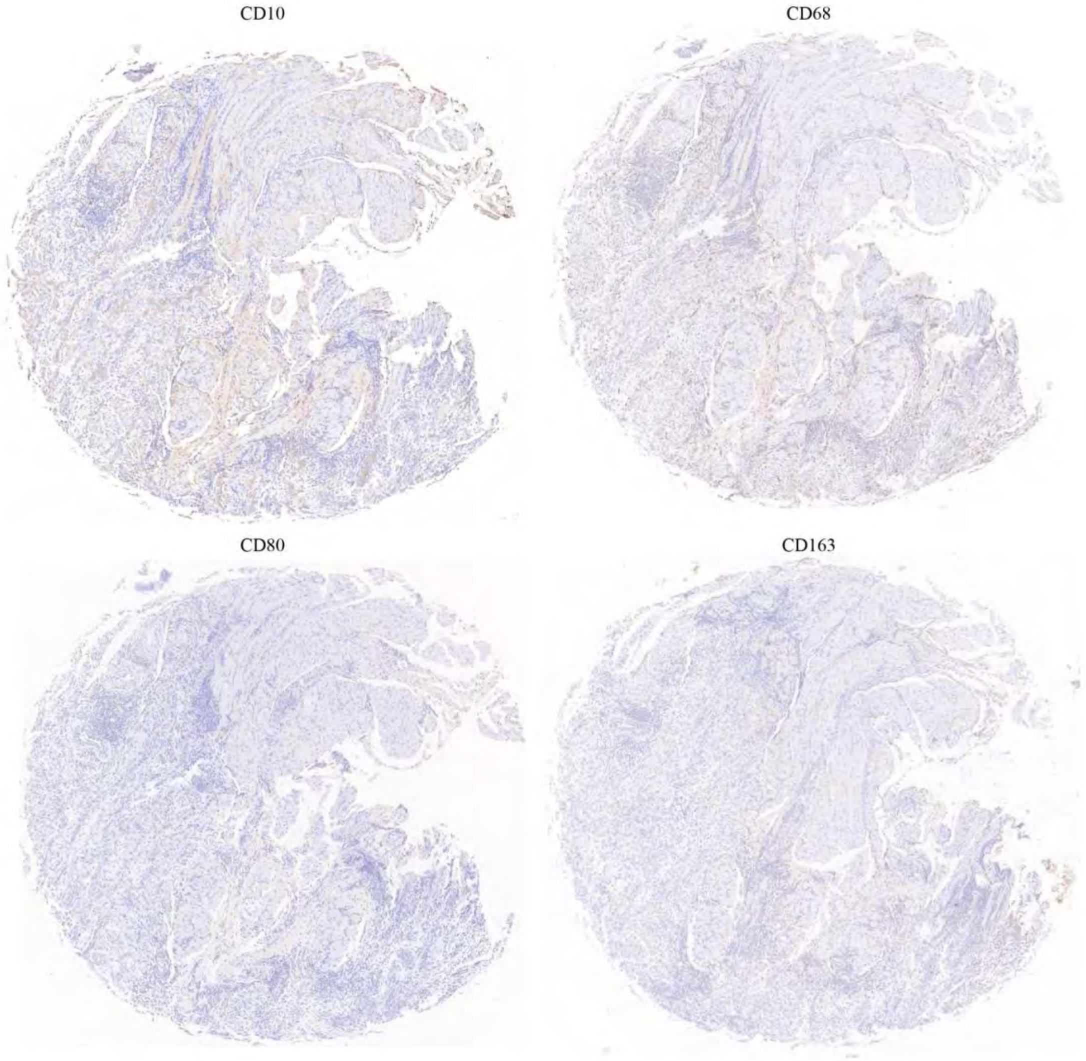

Case C05:

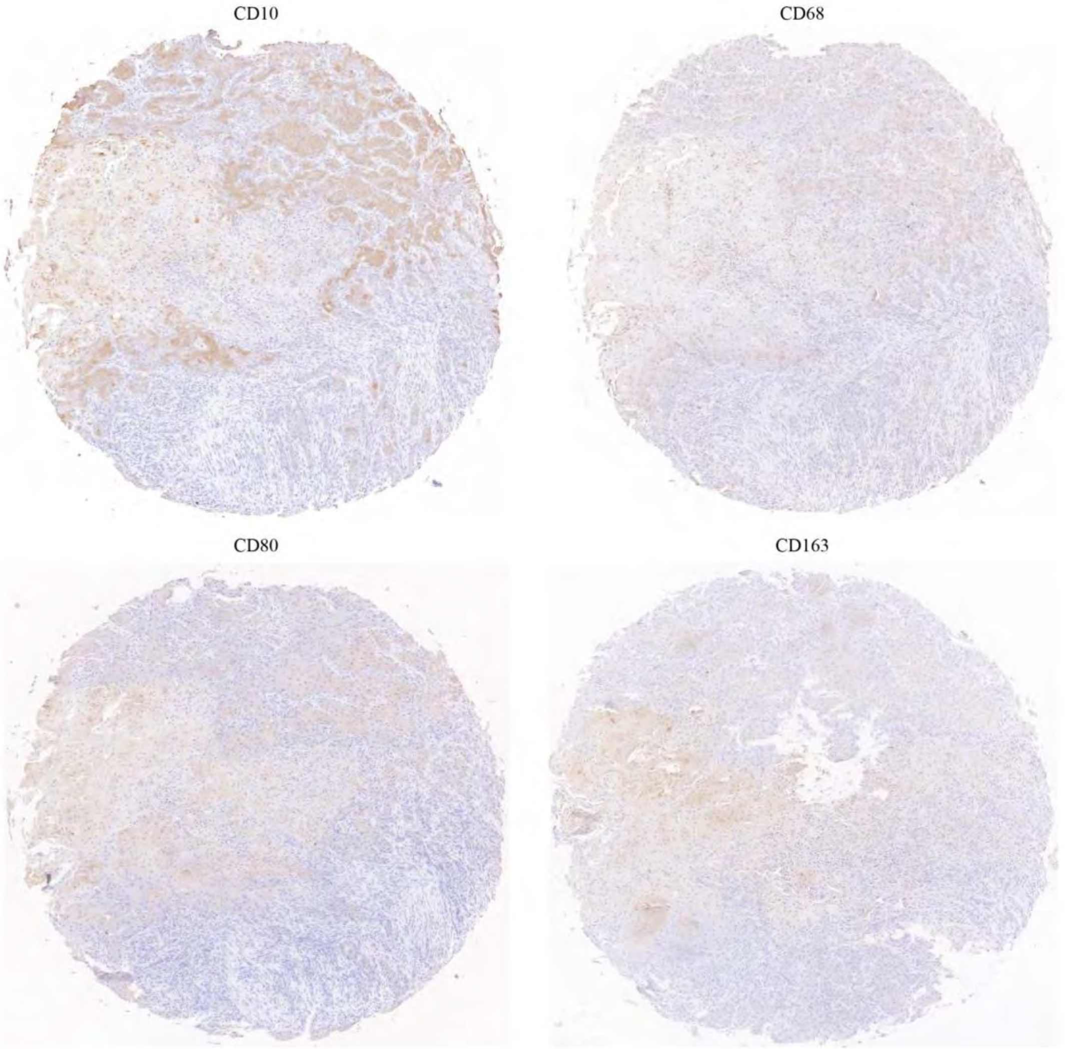

Case C07:

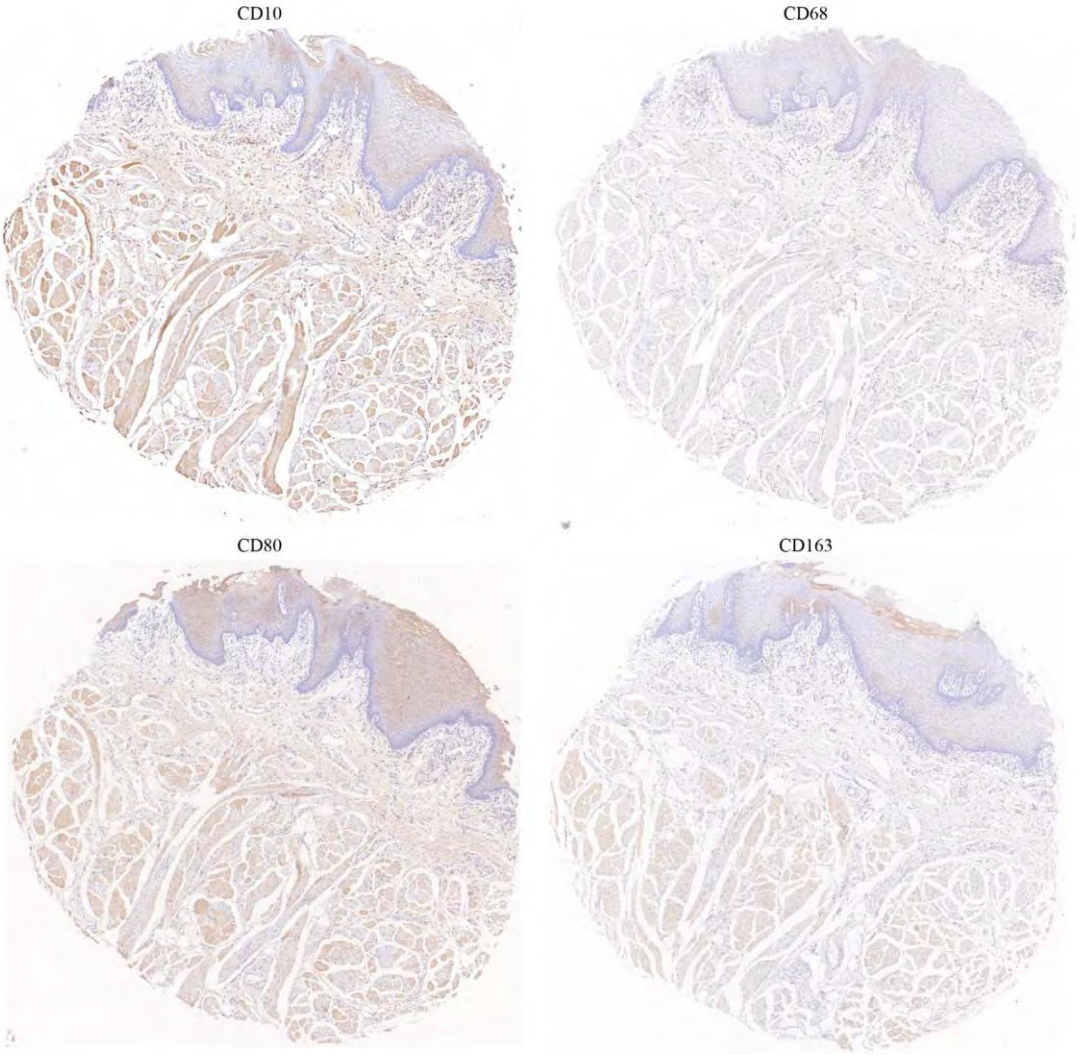

Case C09:

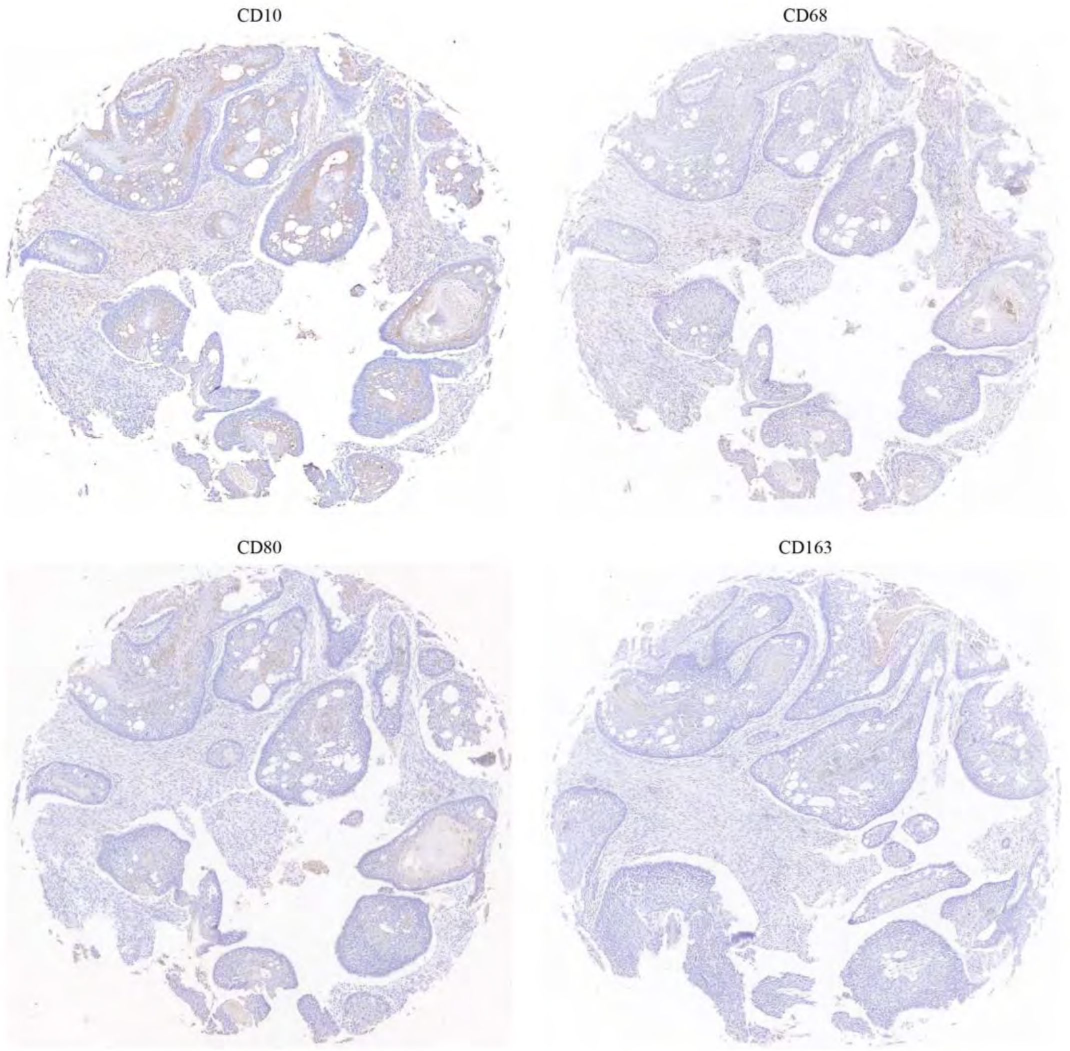

Case C11:

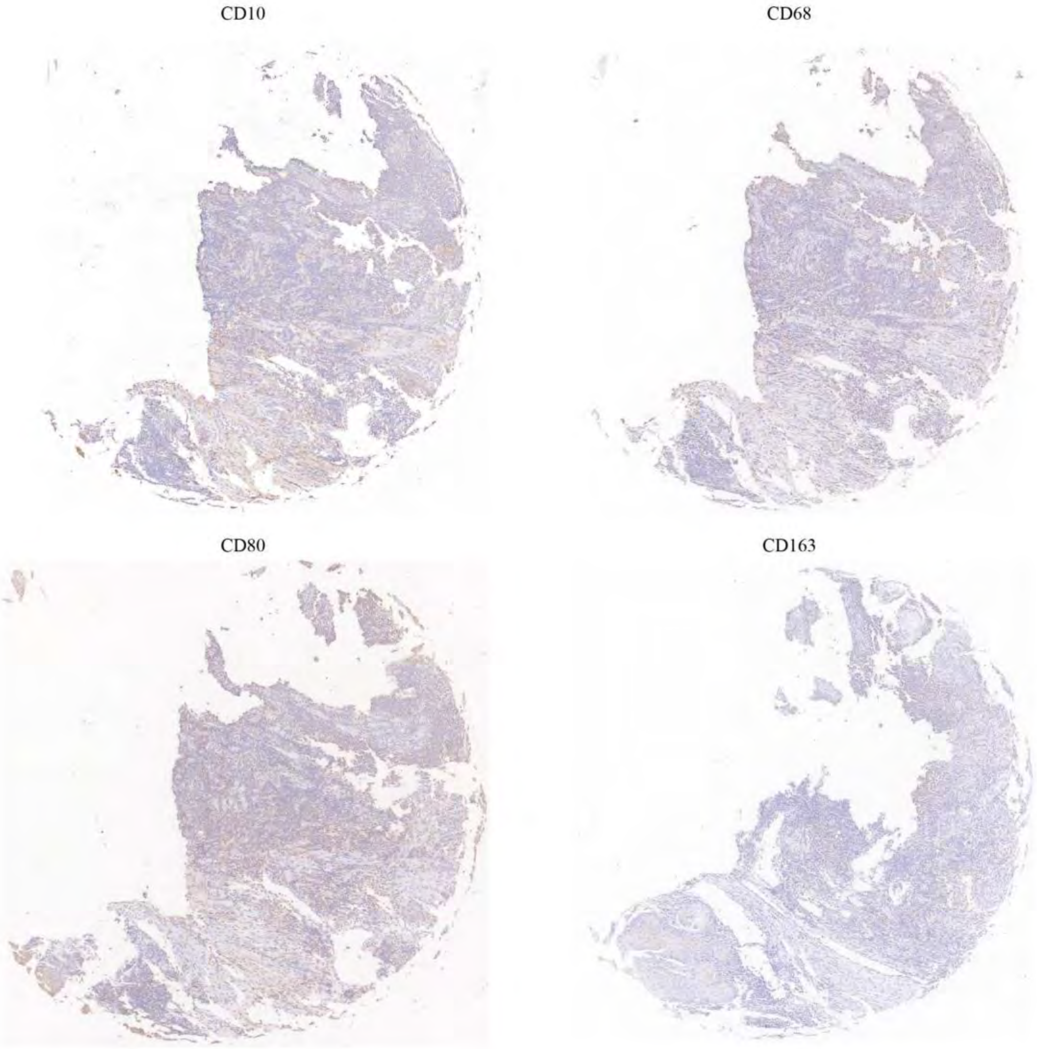

Case D01:

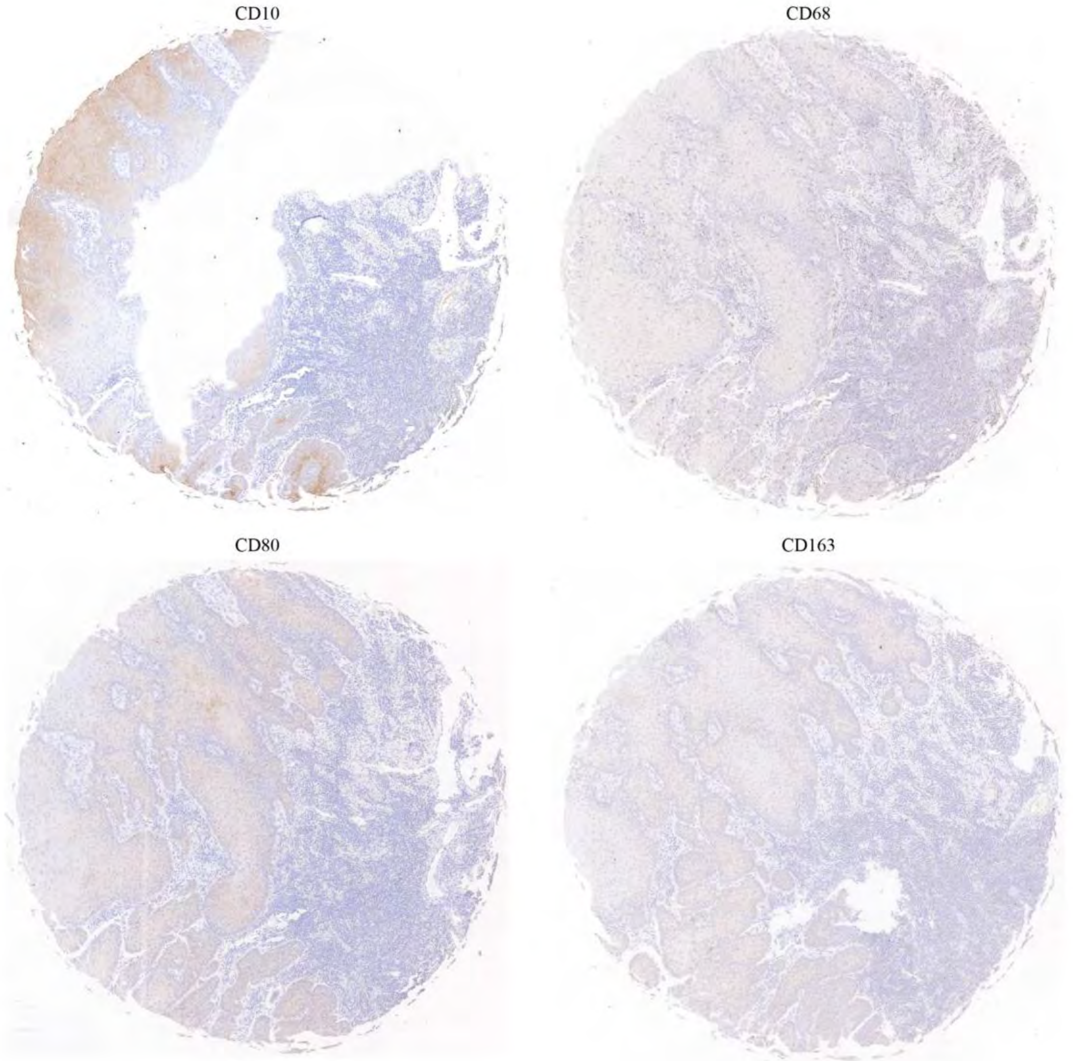

Case D03:

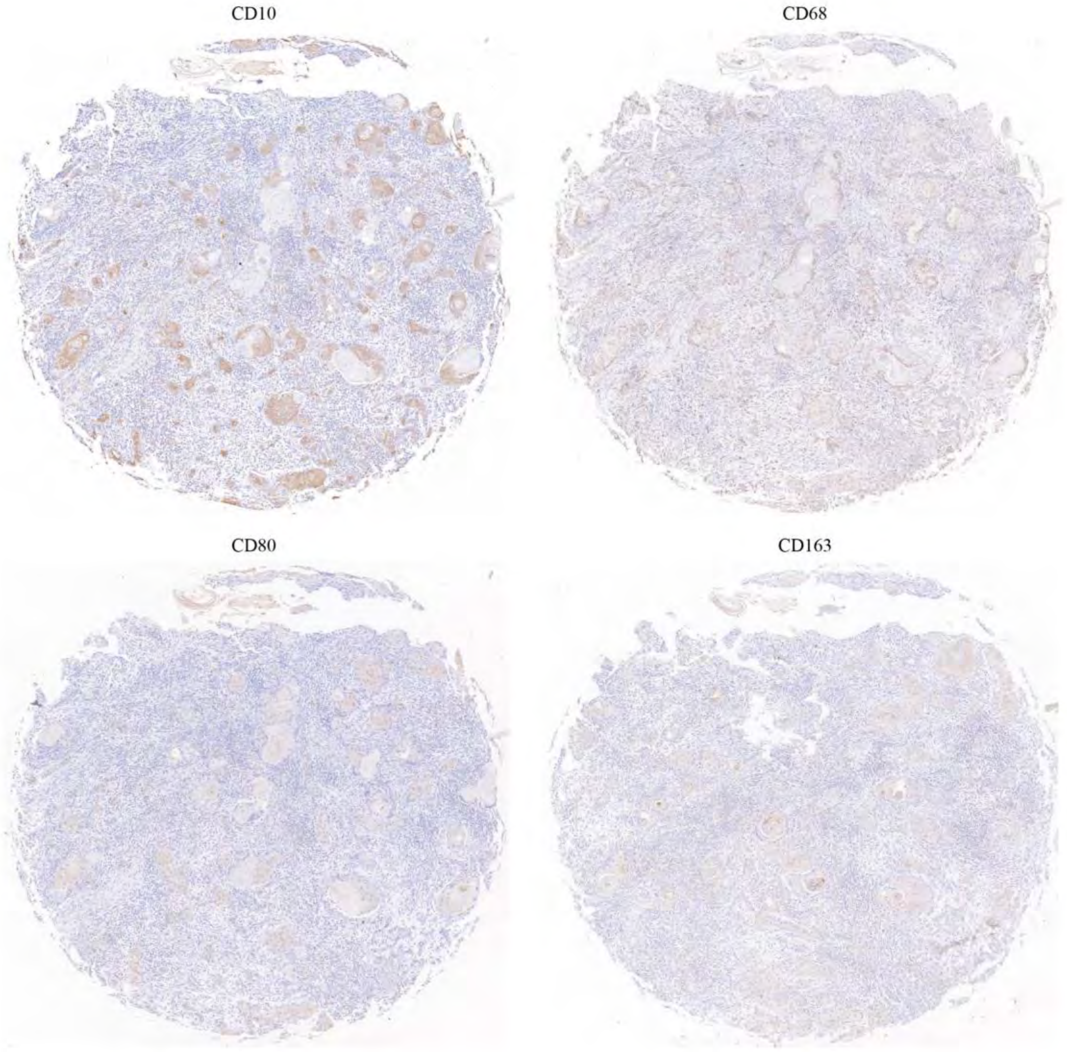

Case D05:

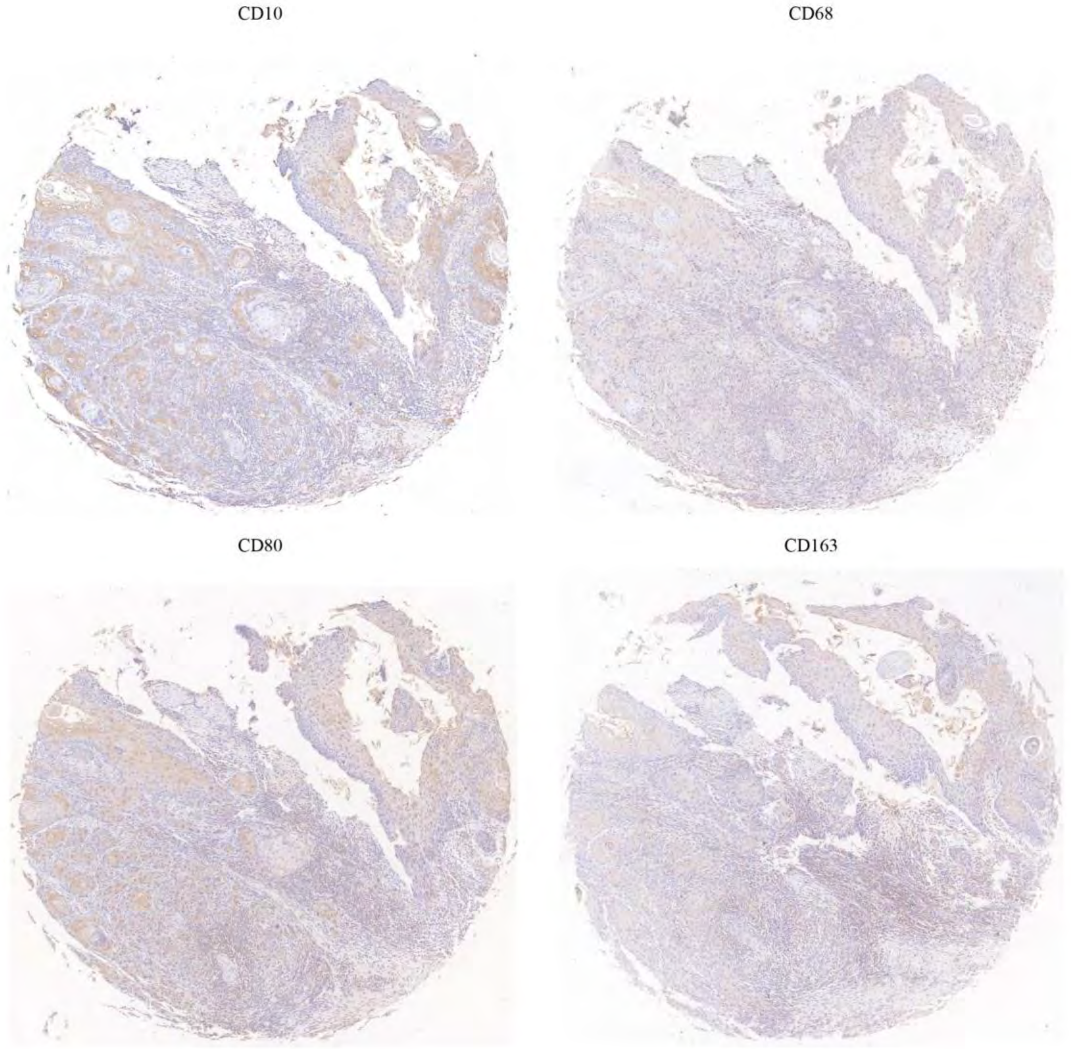

Case D07:

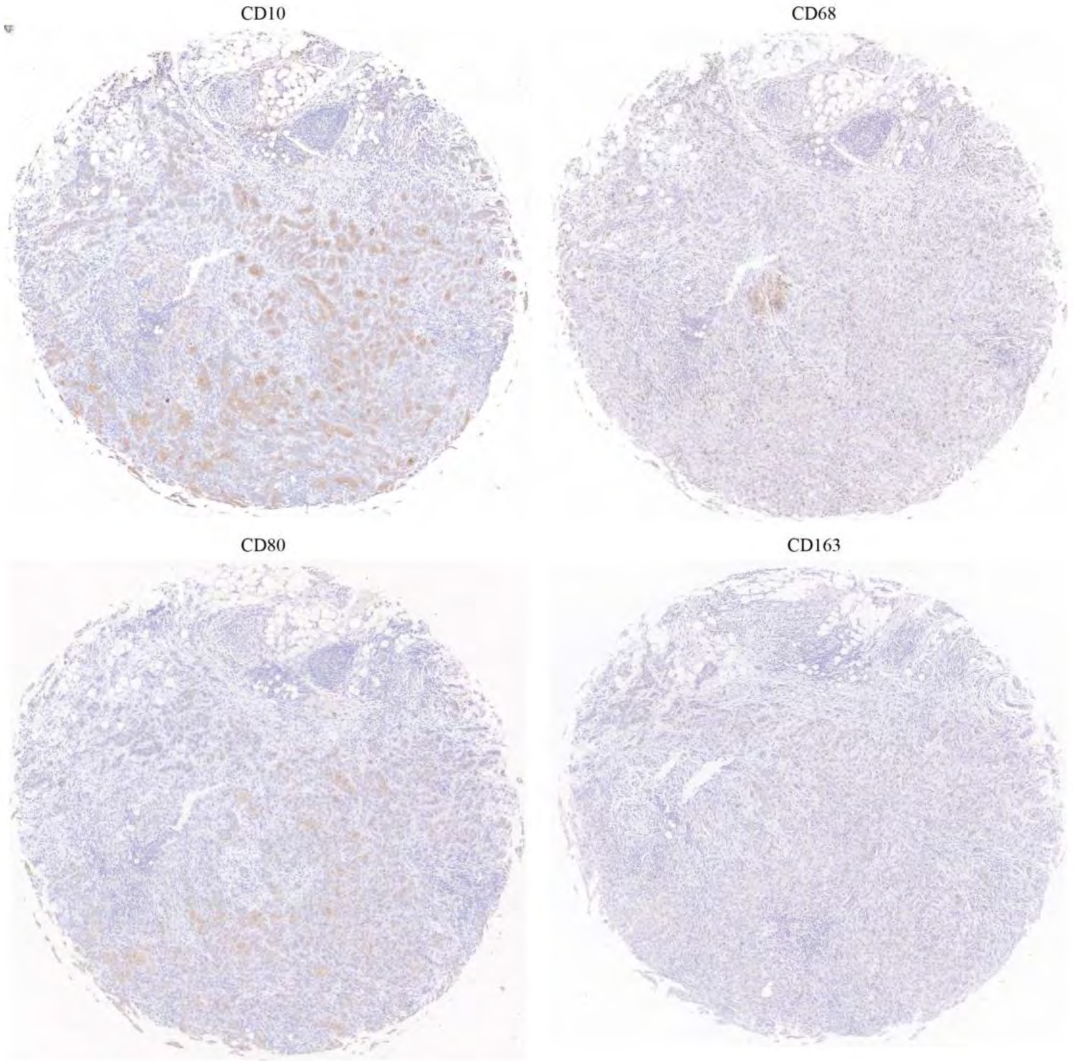

Case D09:

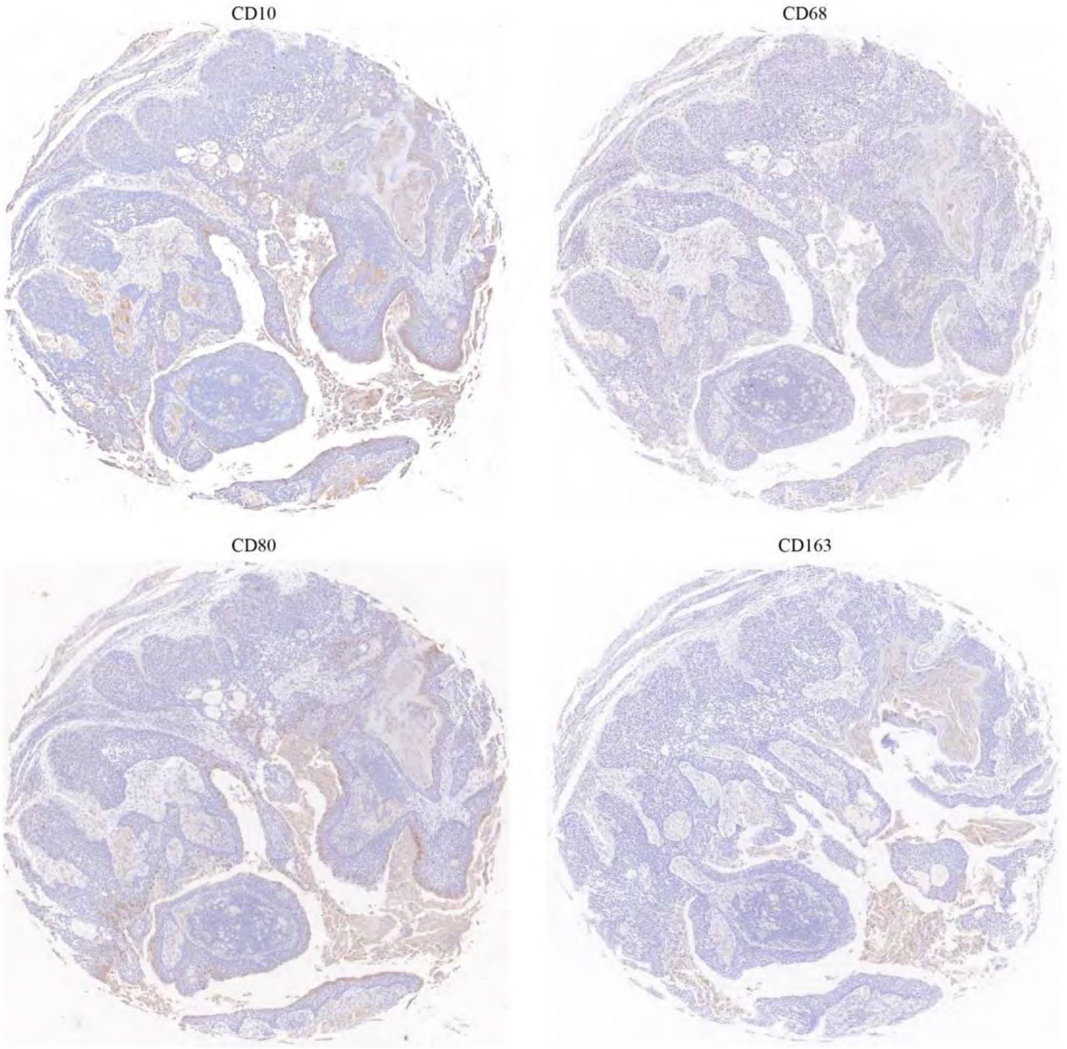

Case D11:

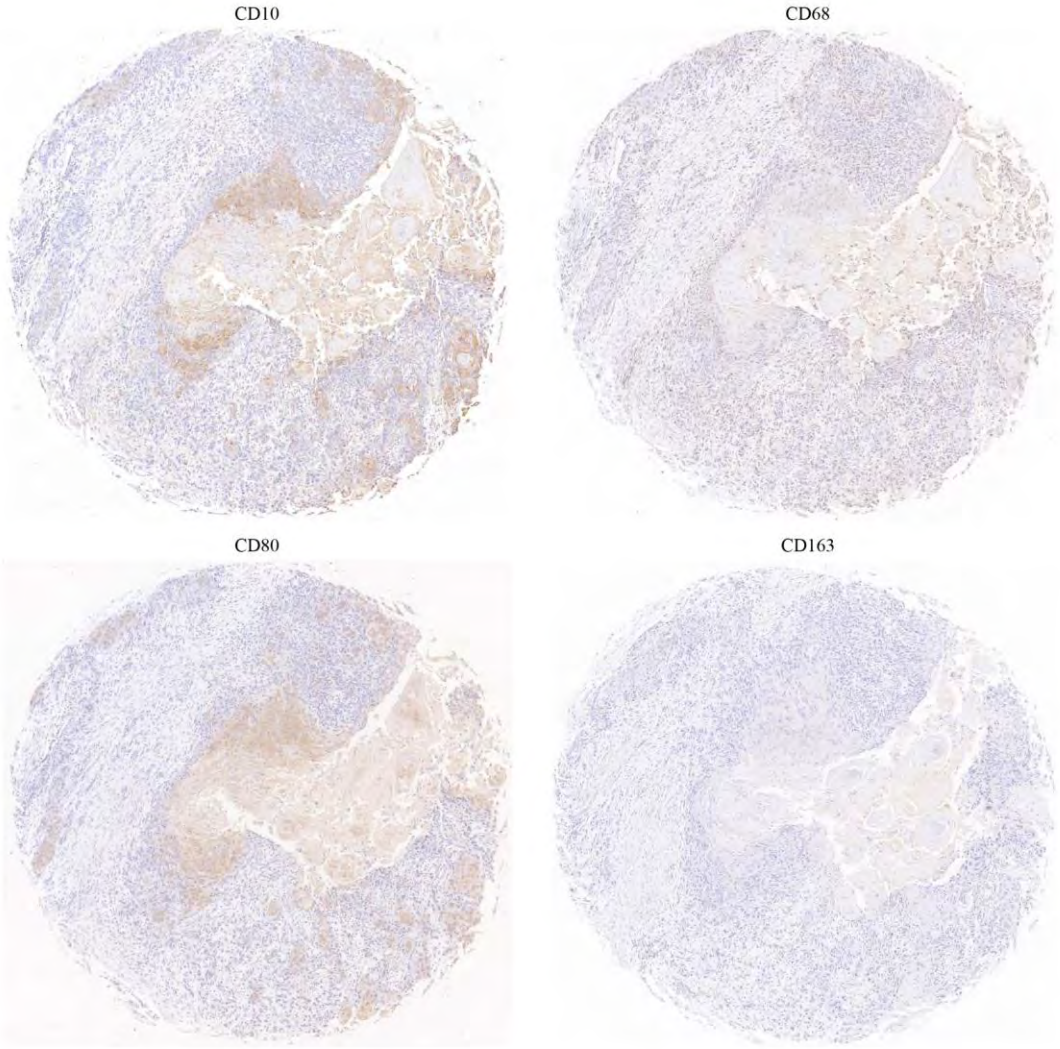

Case E01:

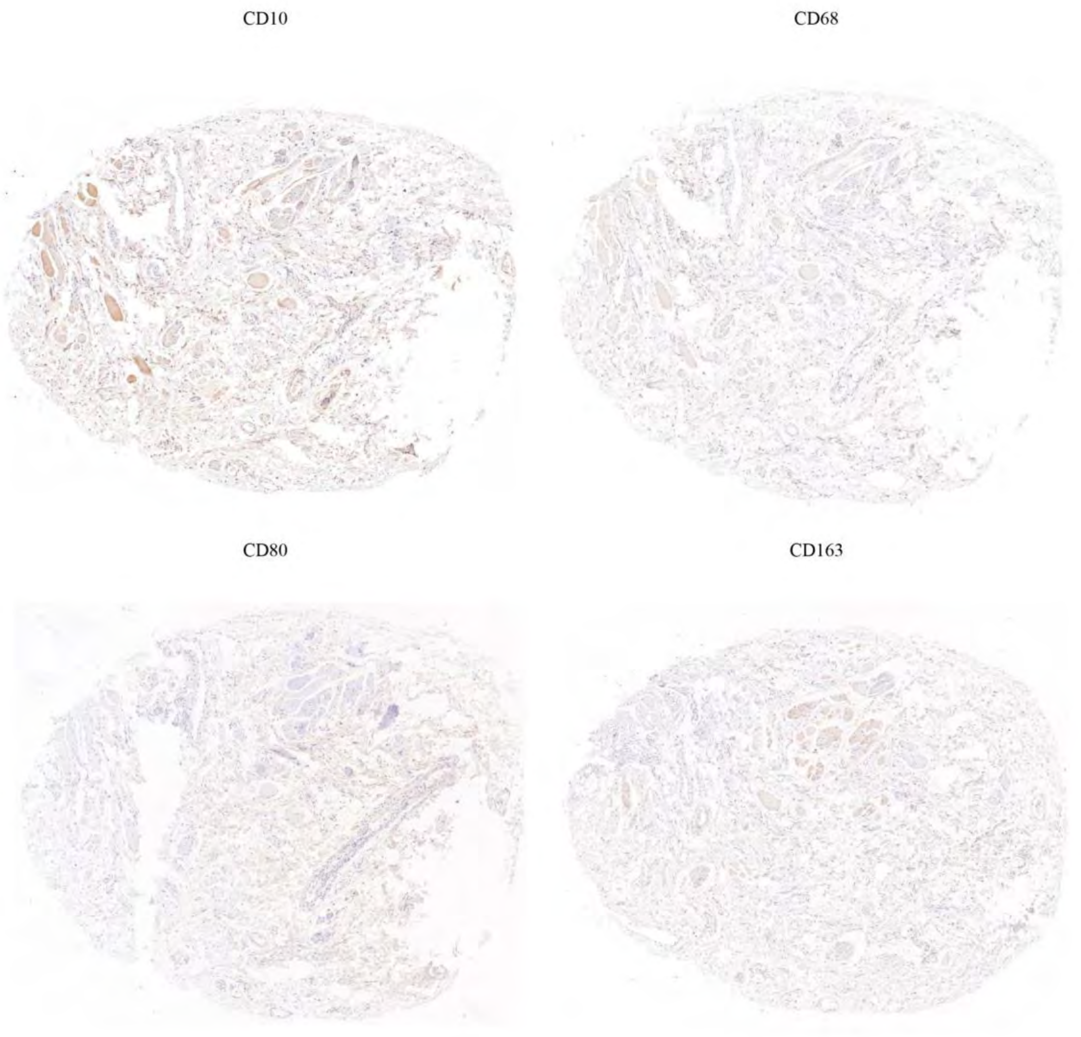

Case E03:

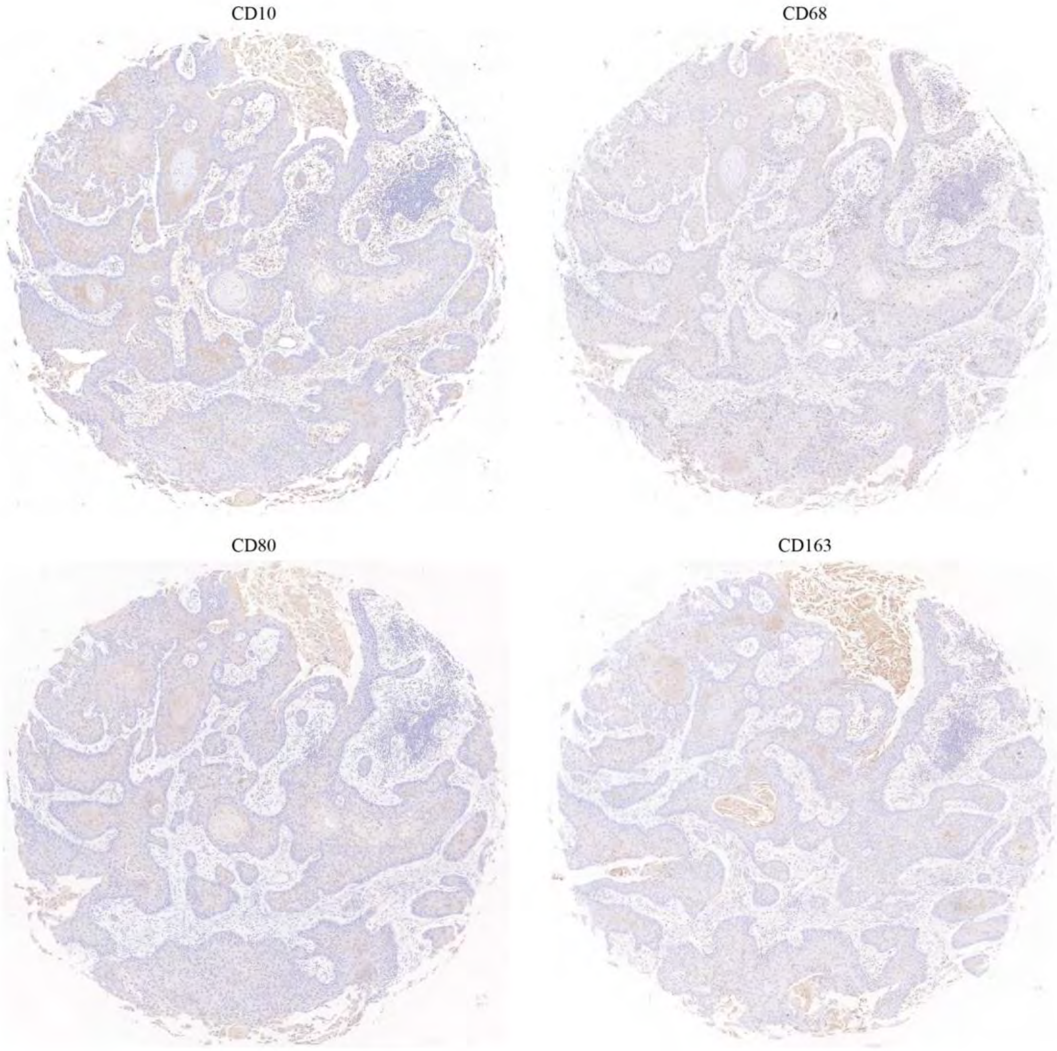

Case E05:

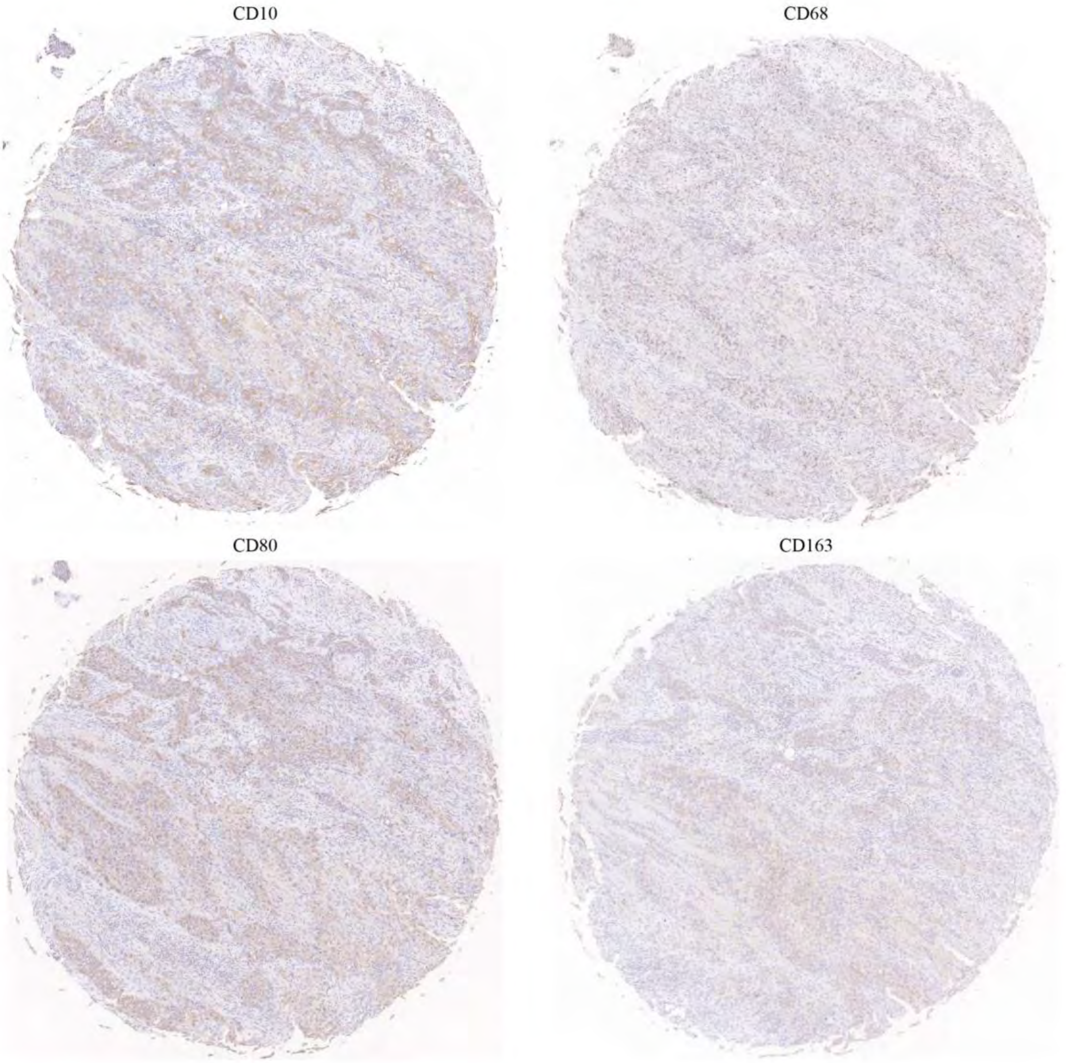

Case E07:

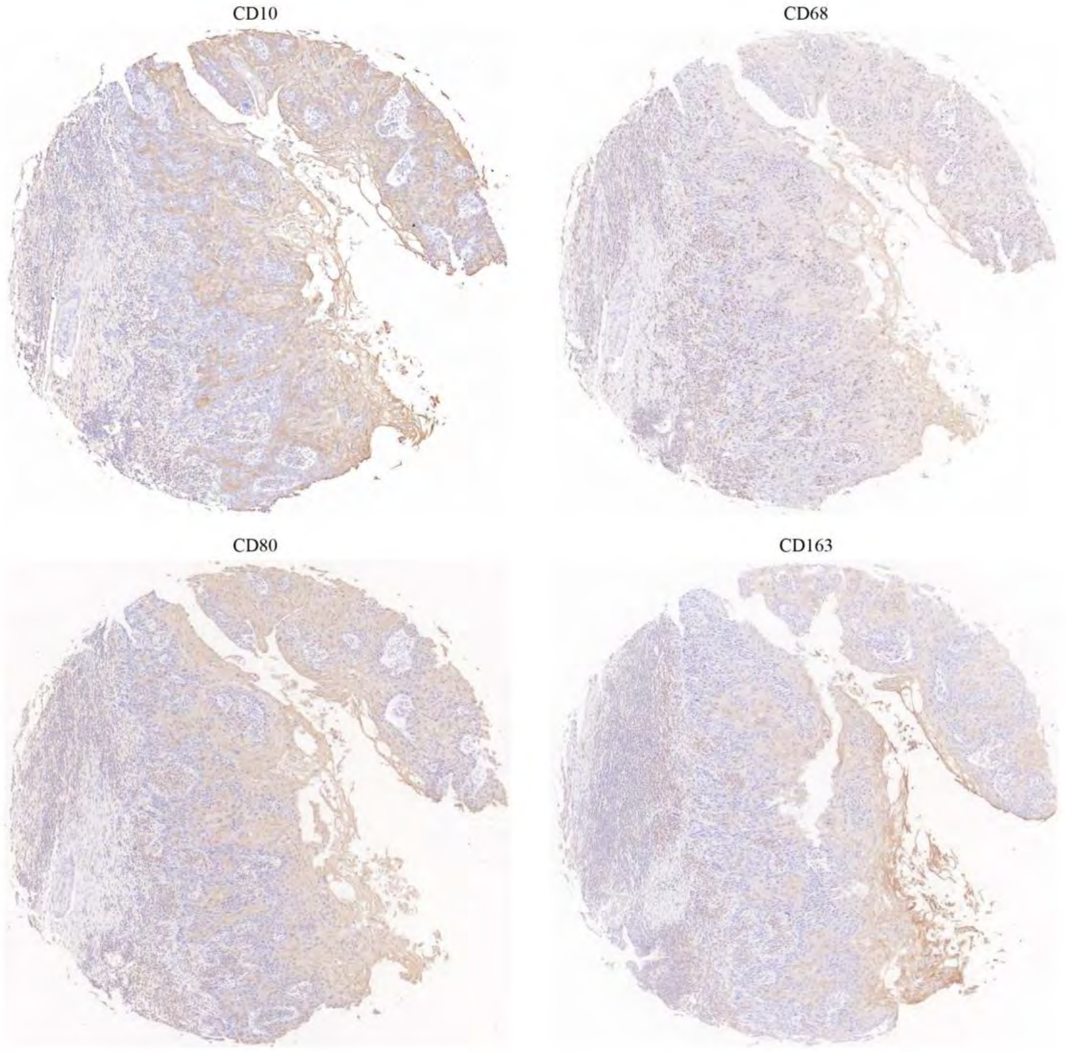

Case E09:

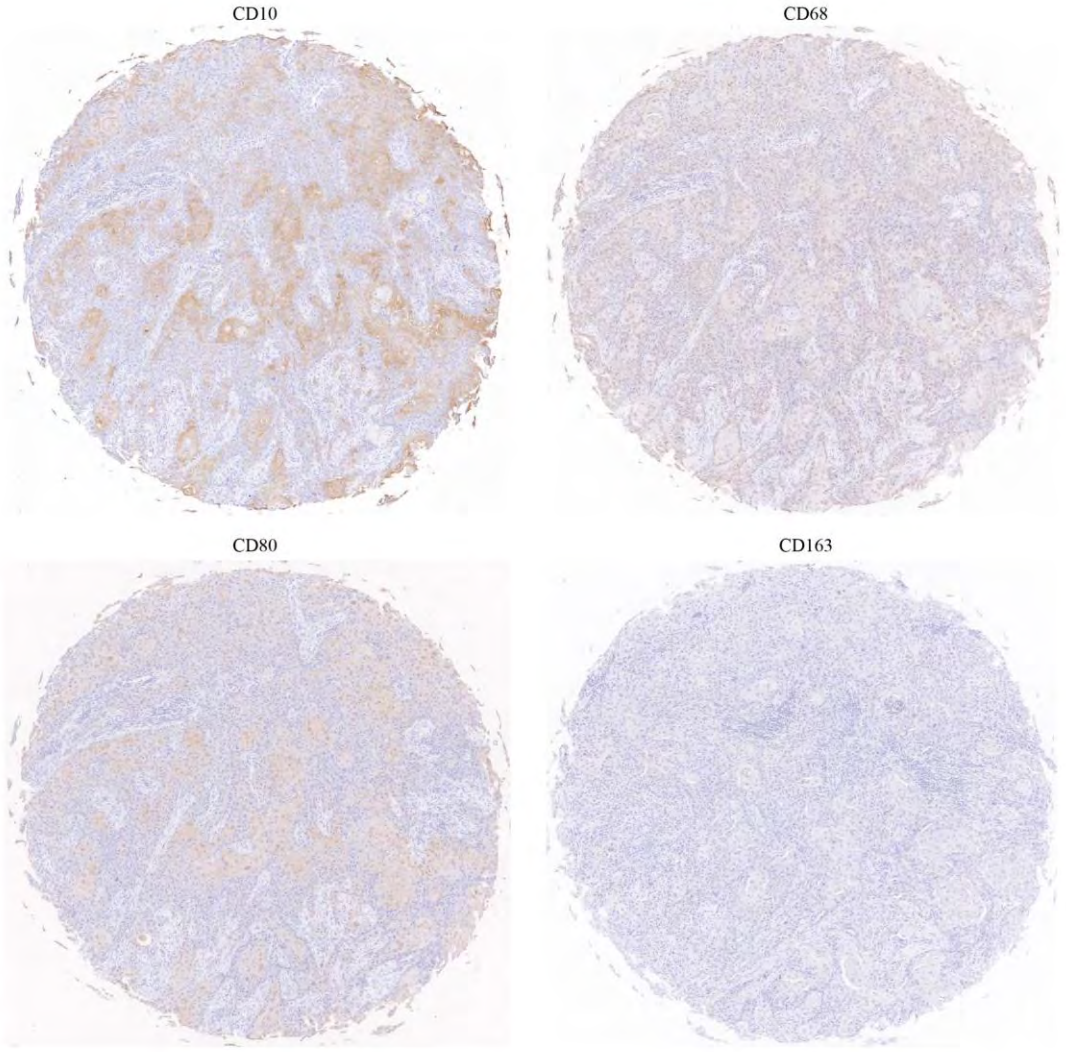

Case E11:

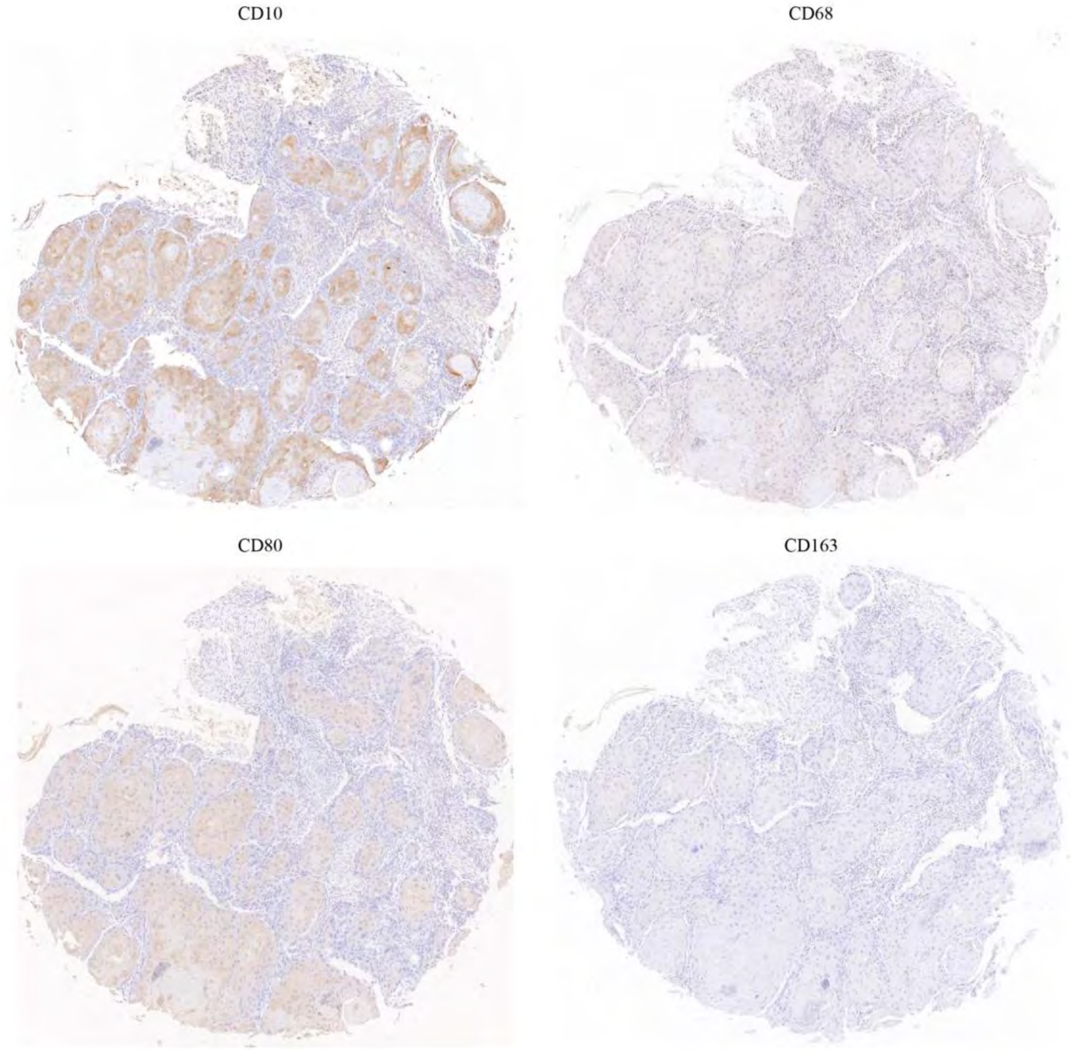

Case F01:

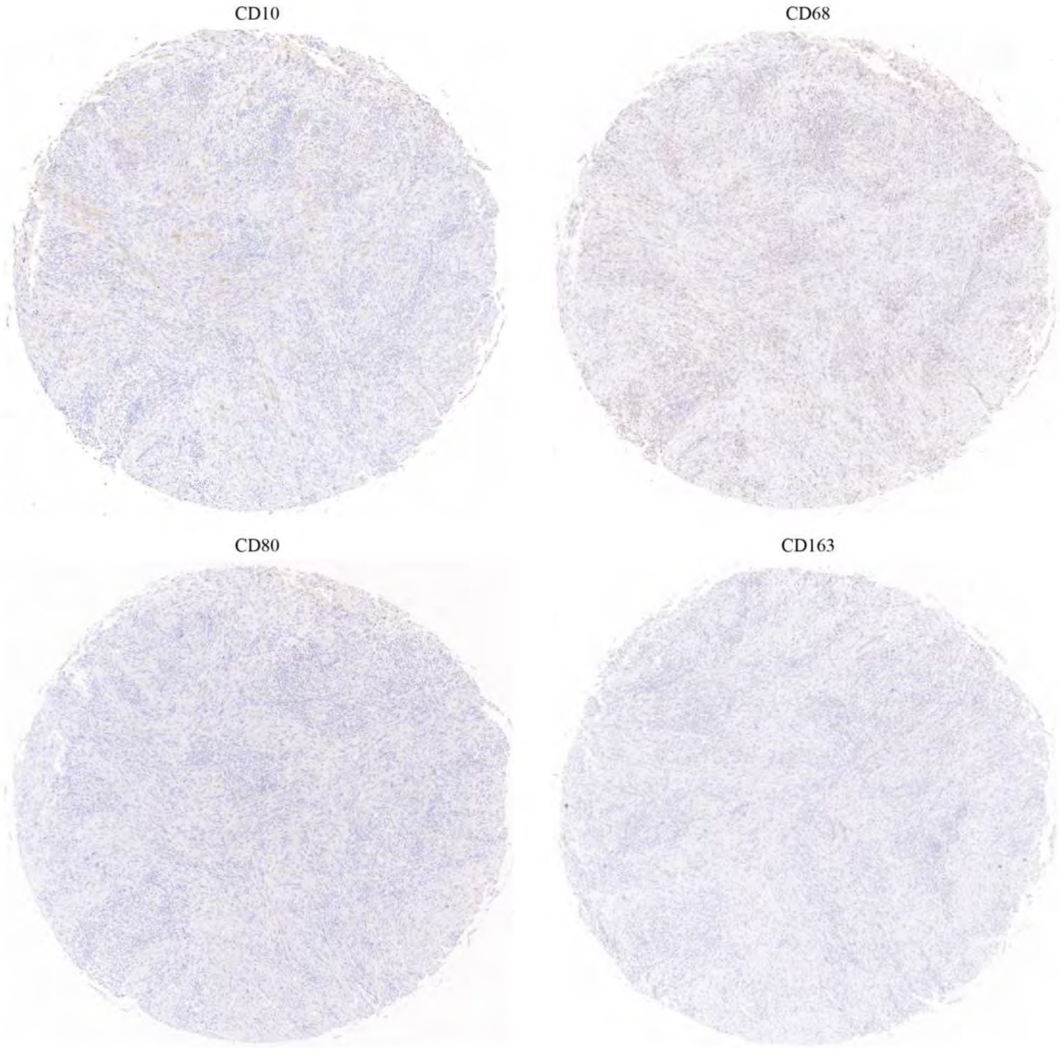

Case F03:

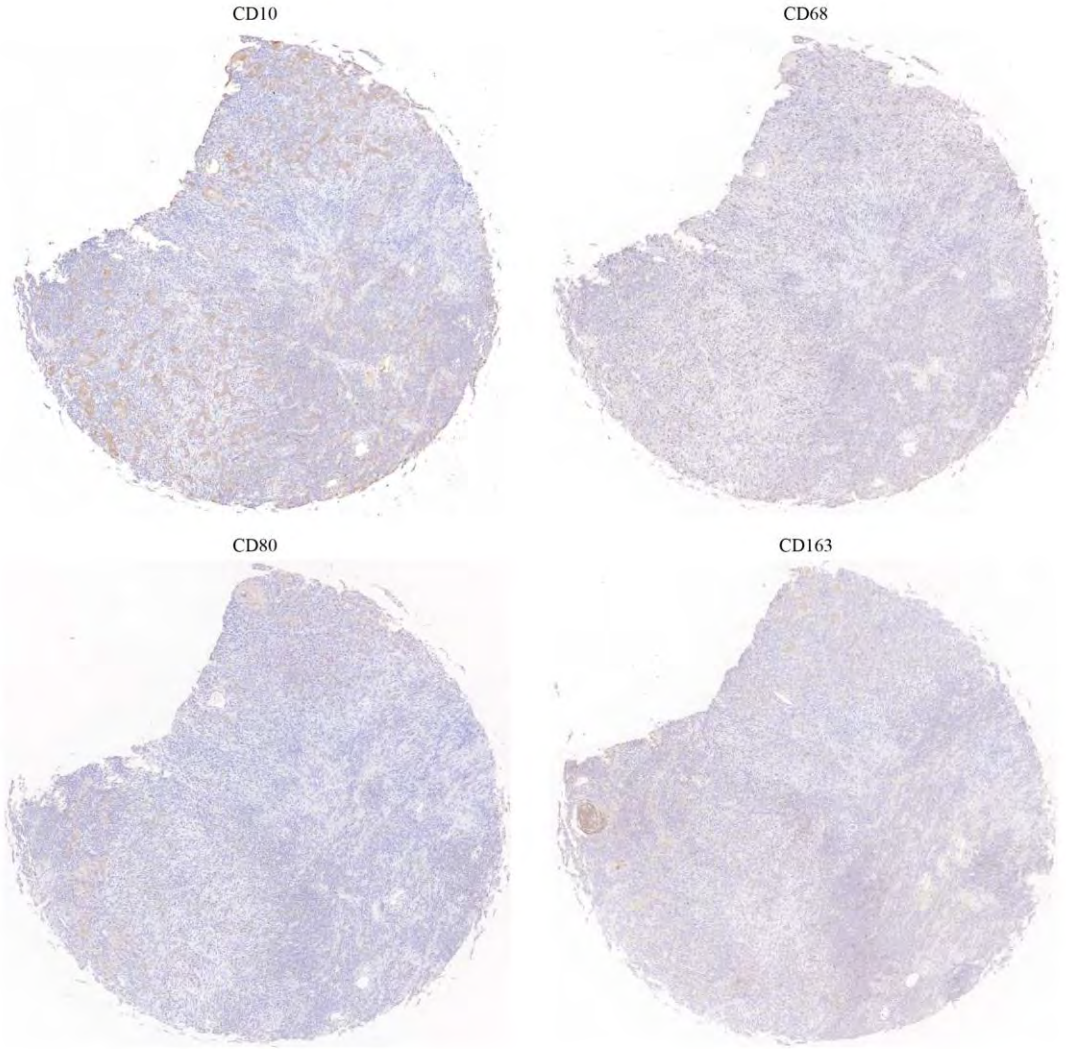

Case F05:

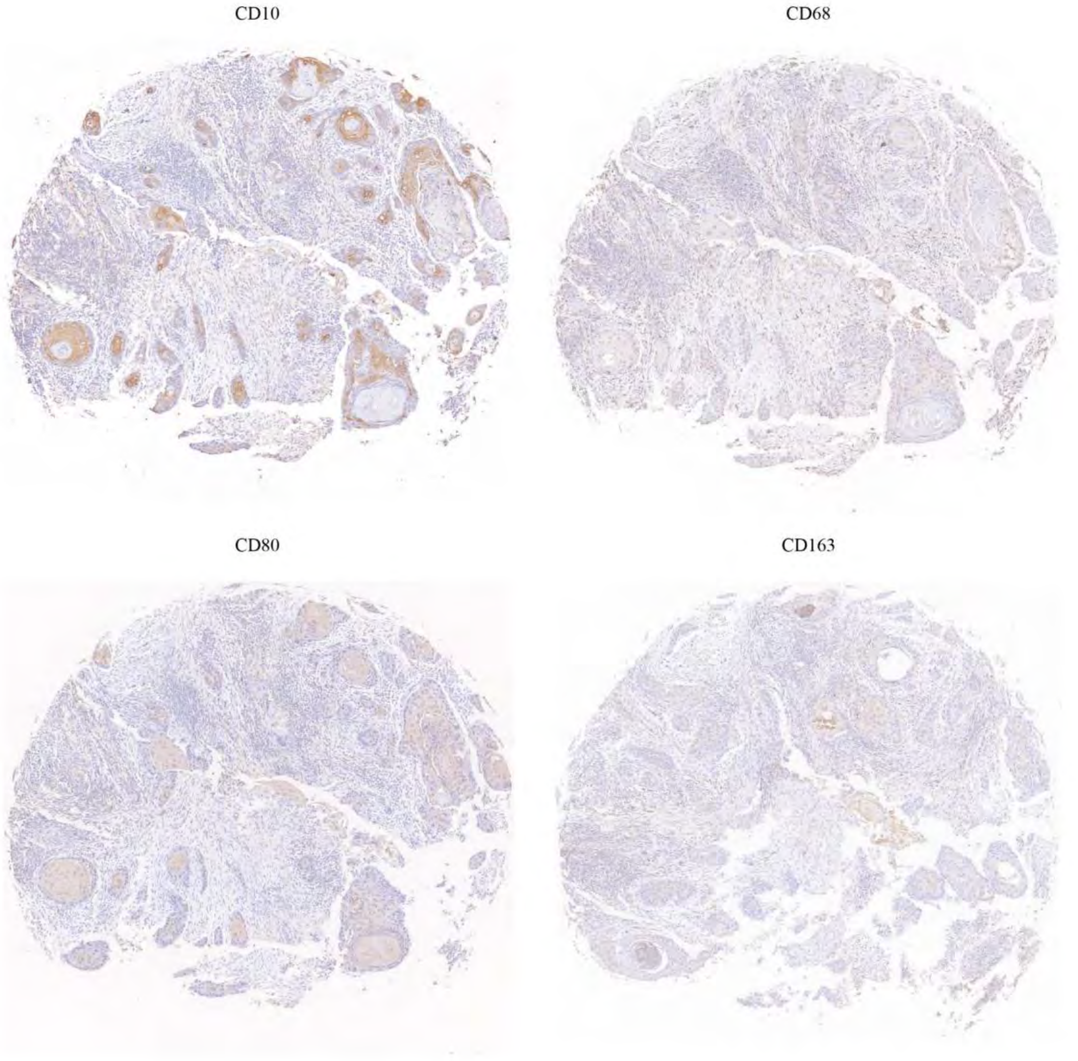

Case F07:

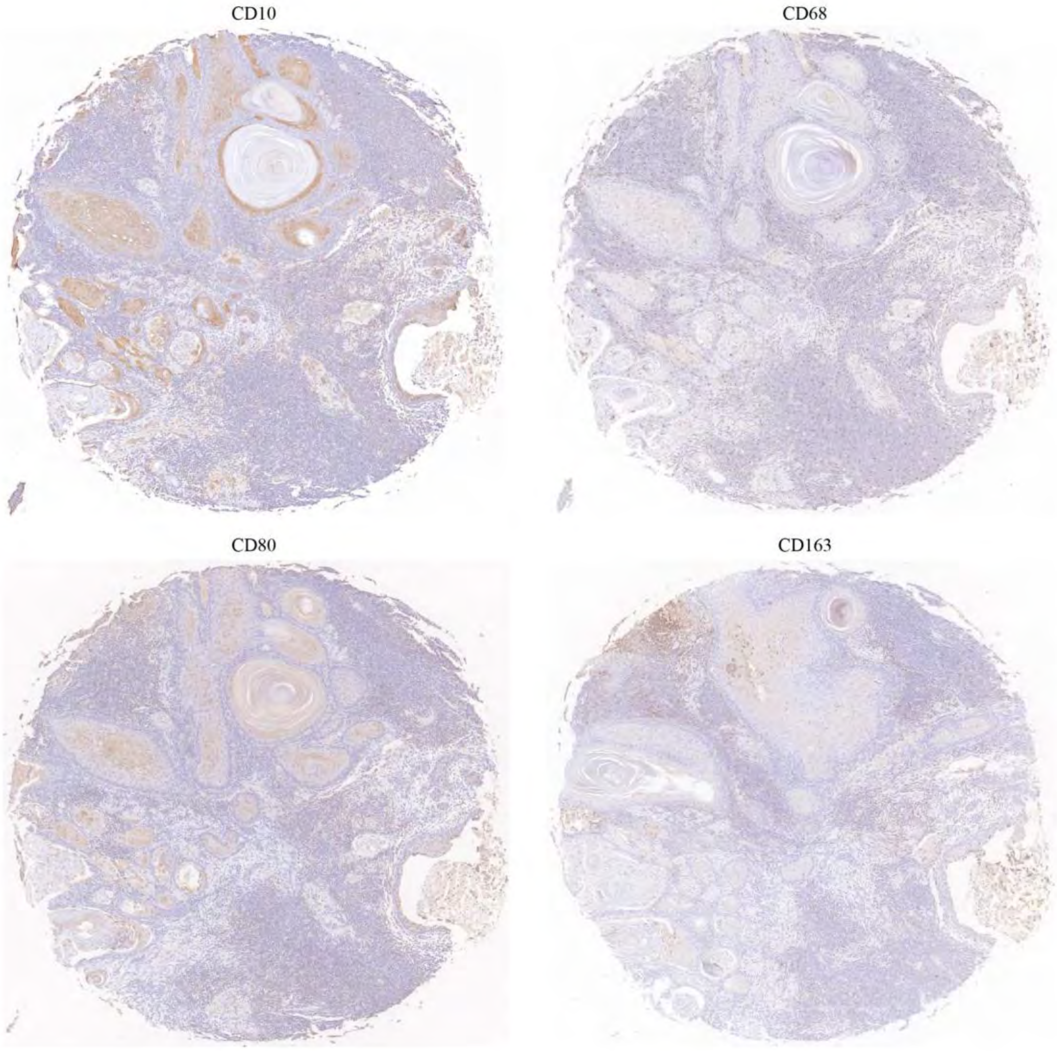

Case F09:

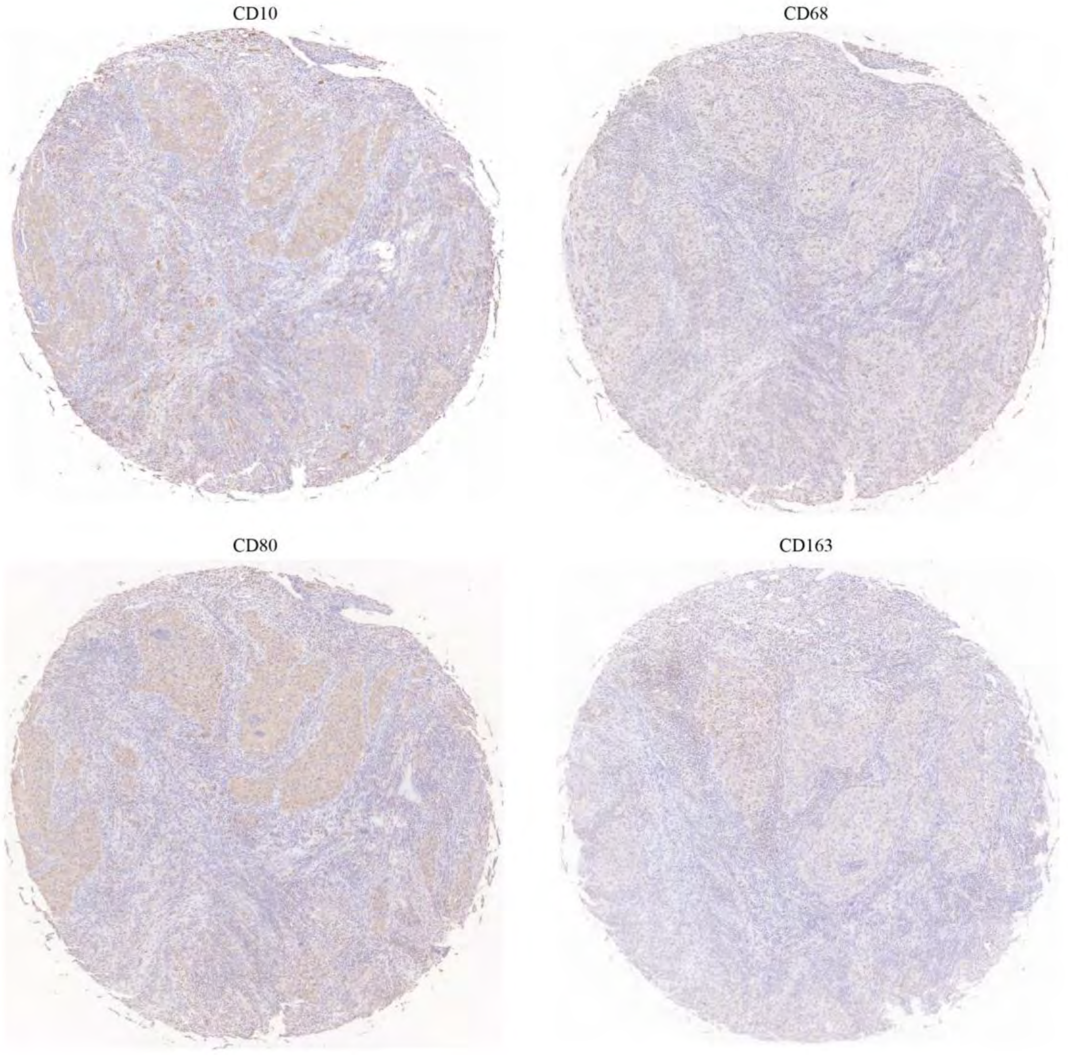

Case F11:

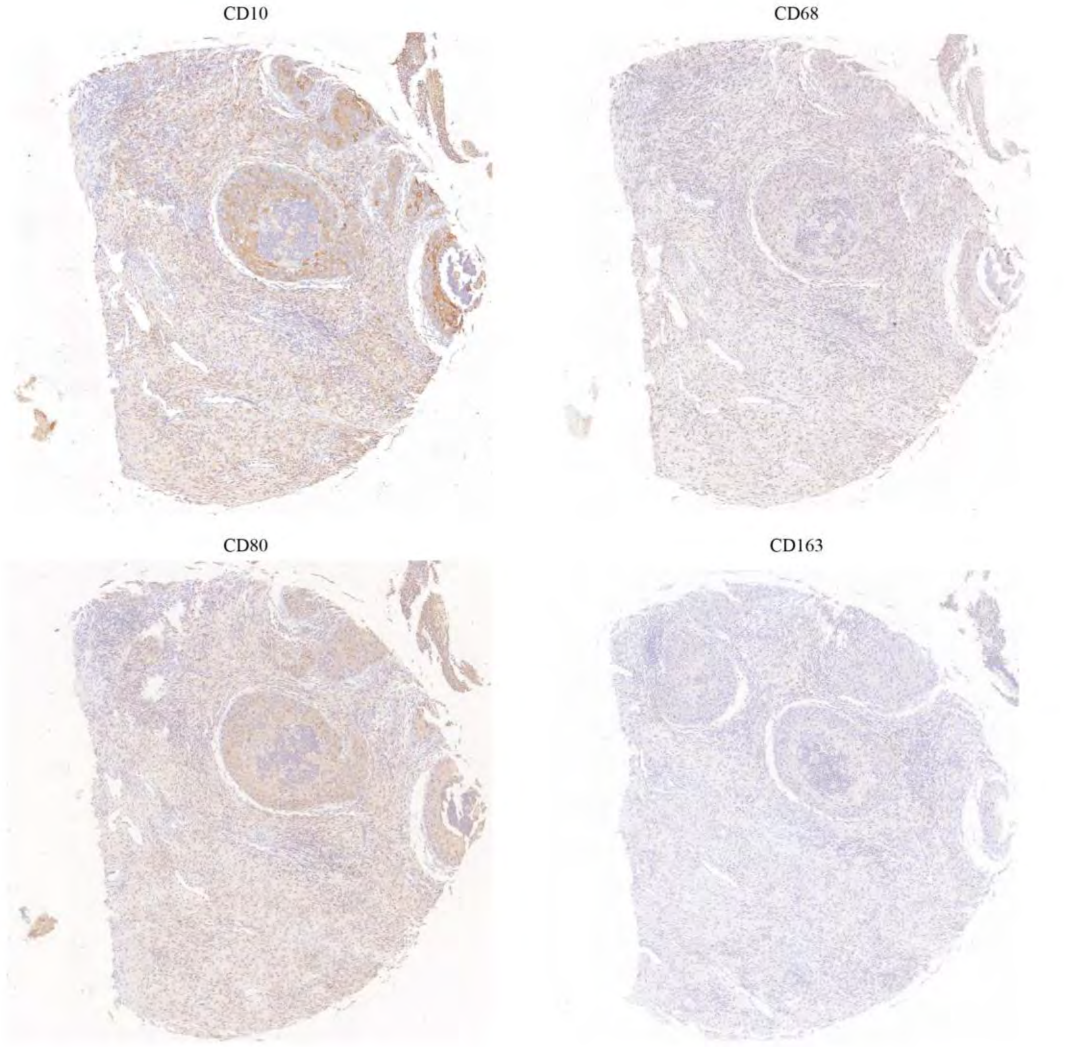

Case G01:

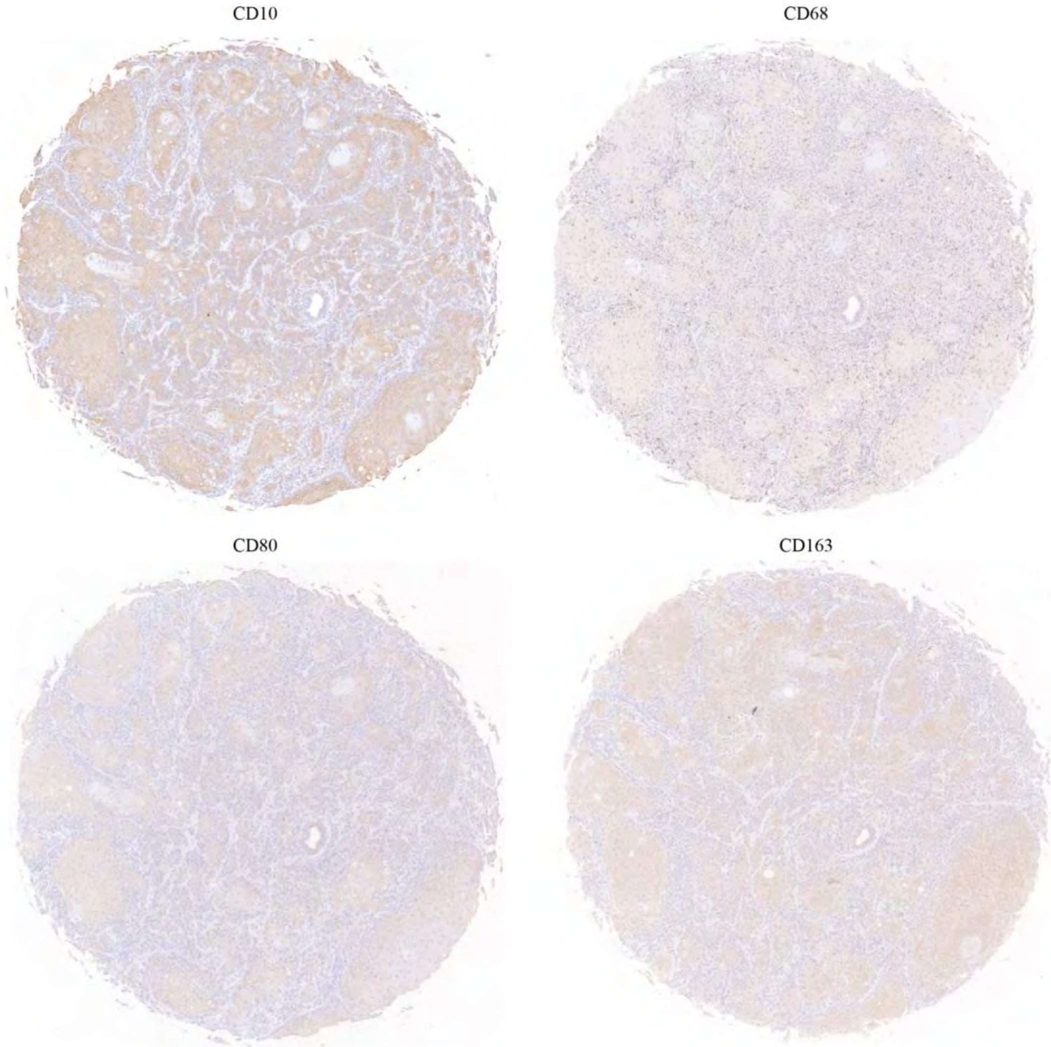

Case G03:

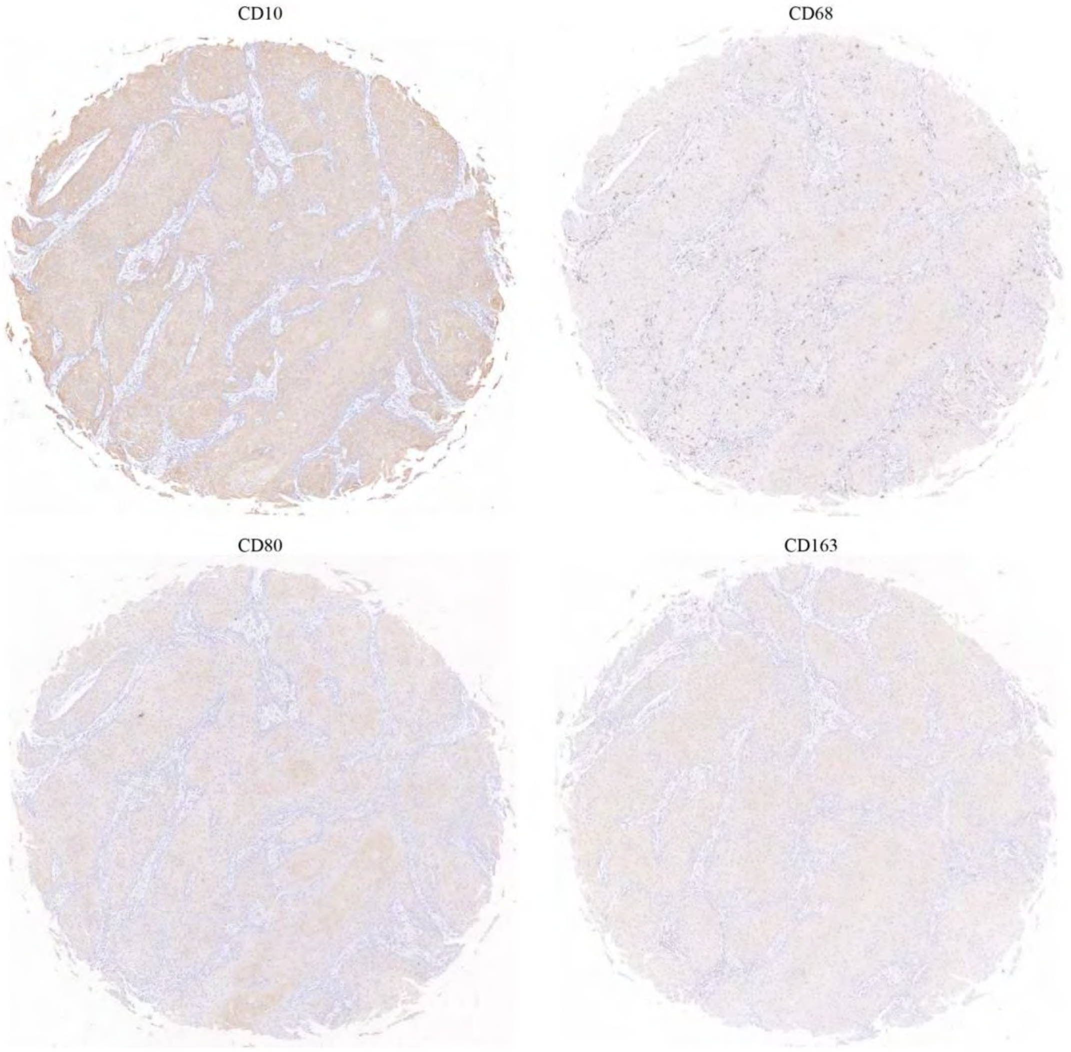

Case G05:

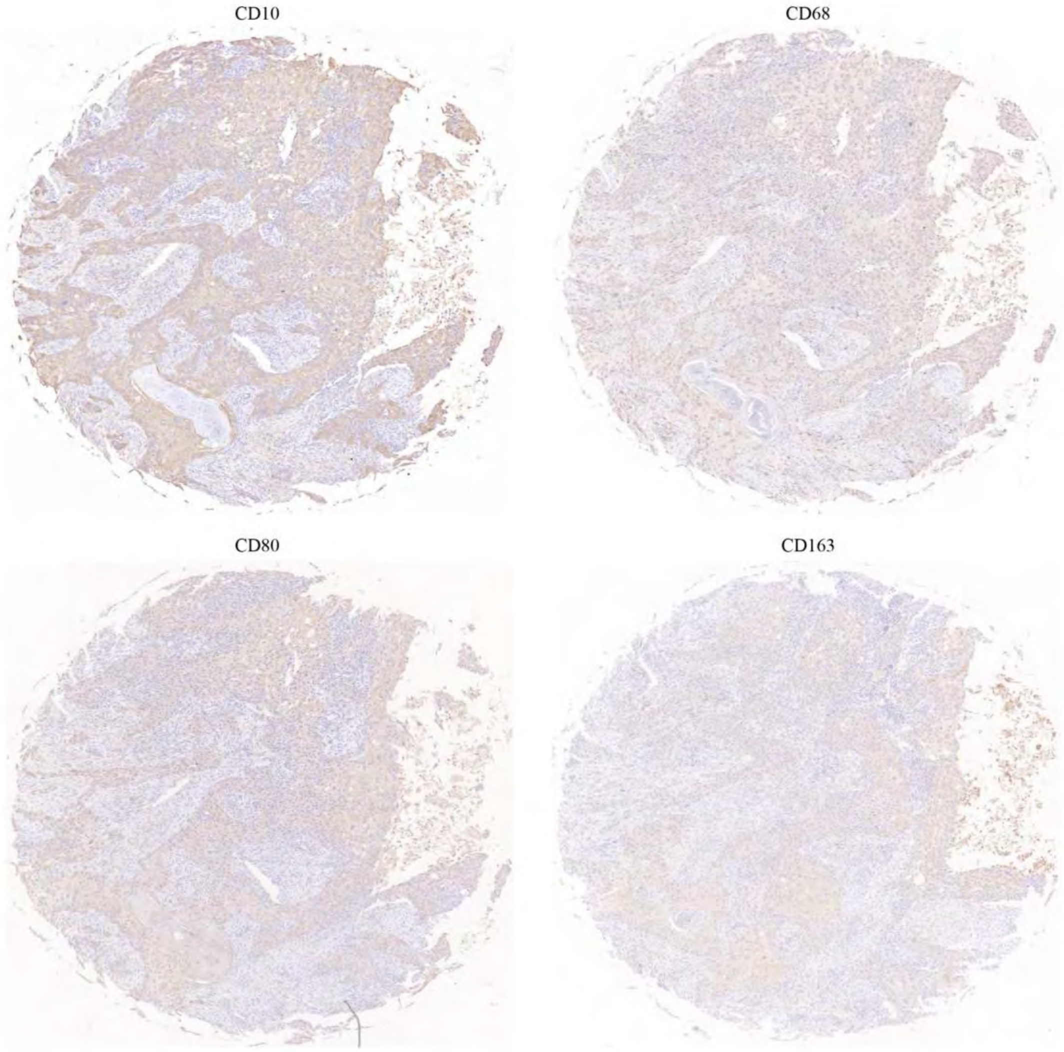

Case G07:

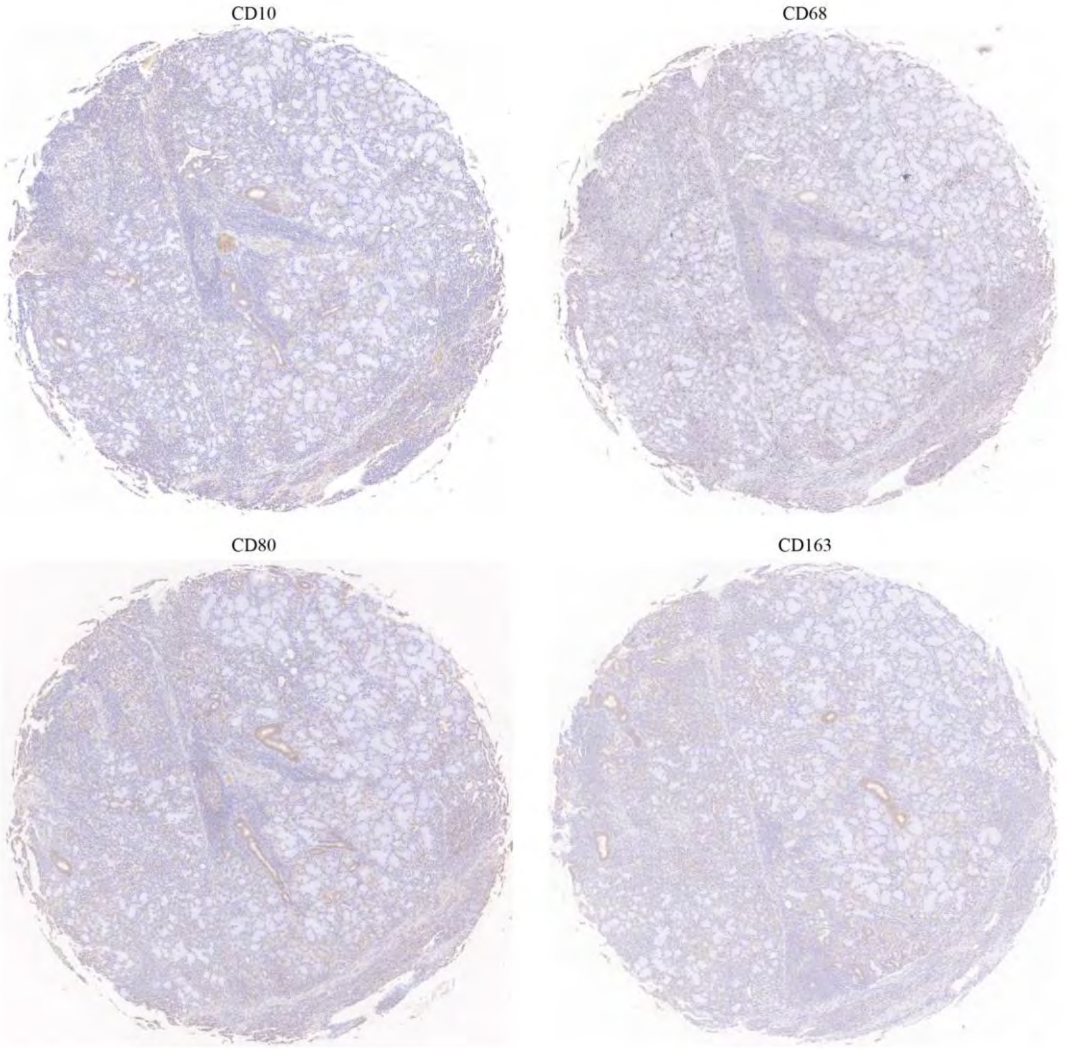

Case G09:

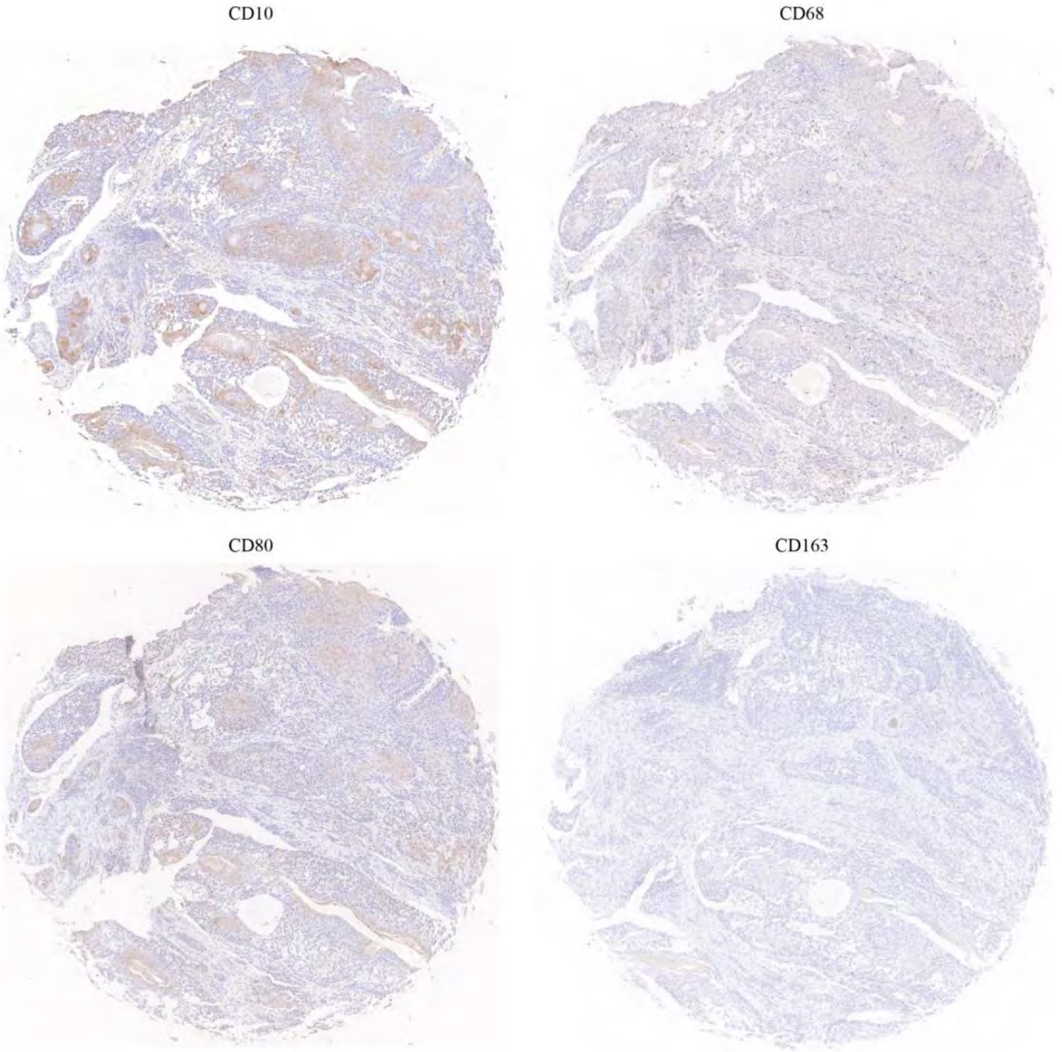

Case G11:

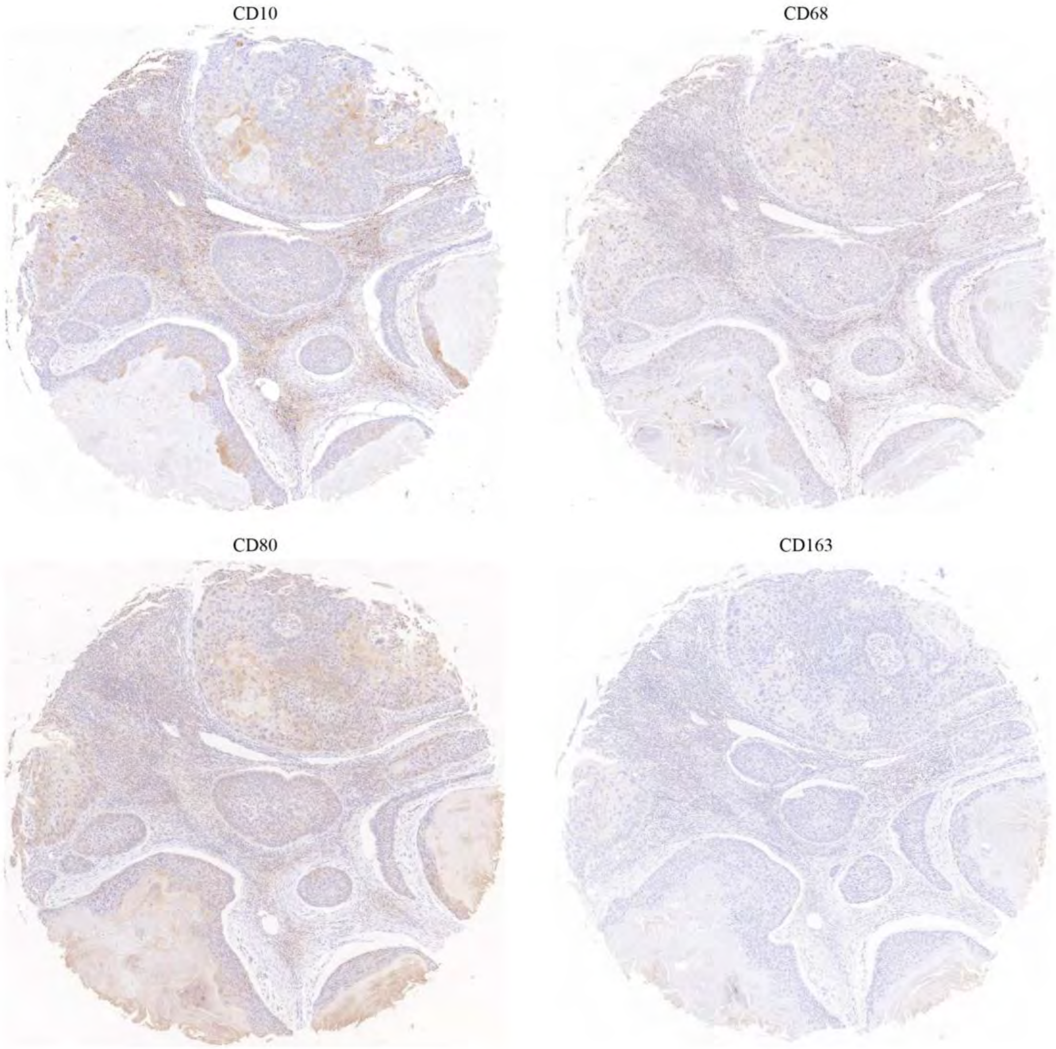

Case H01:

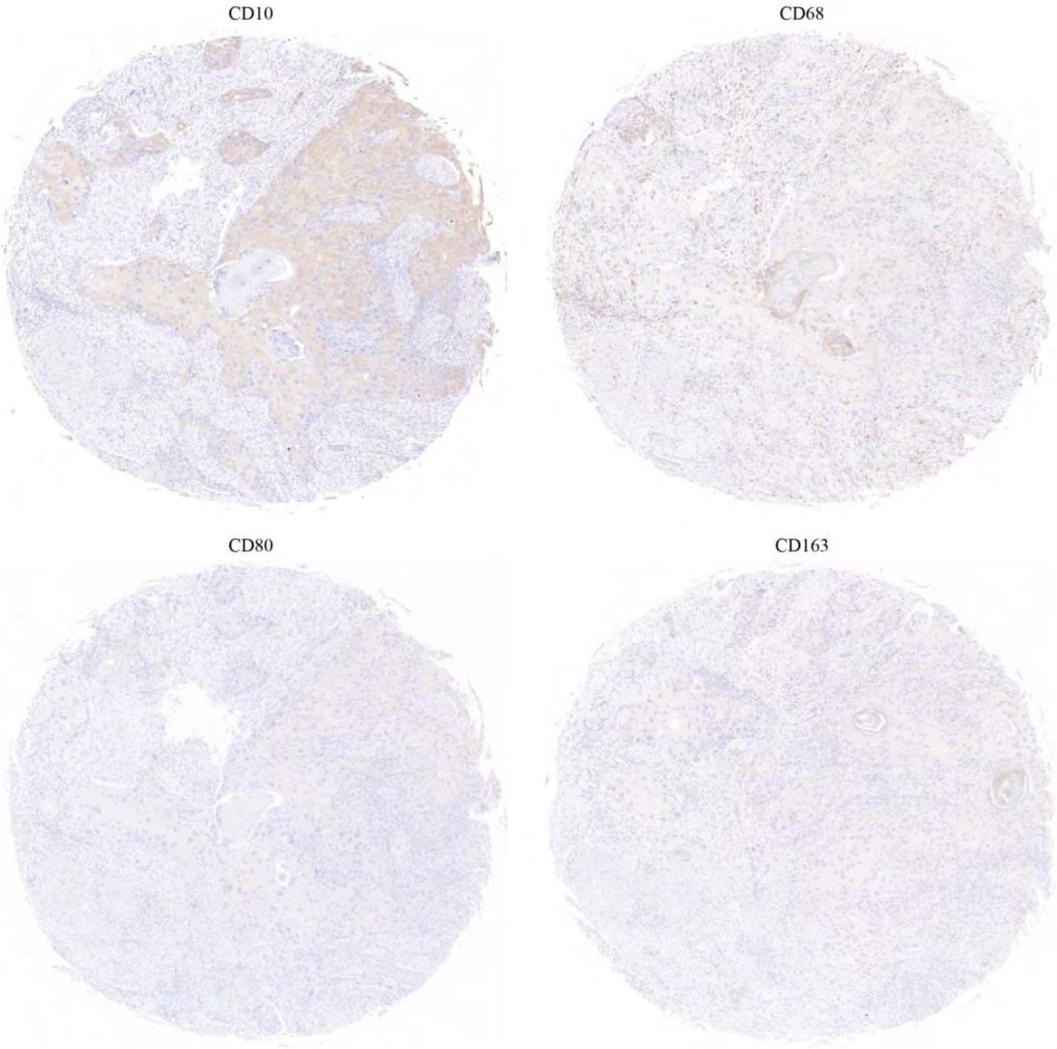

Case H03:

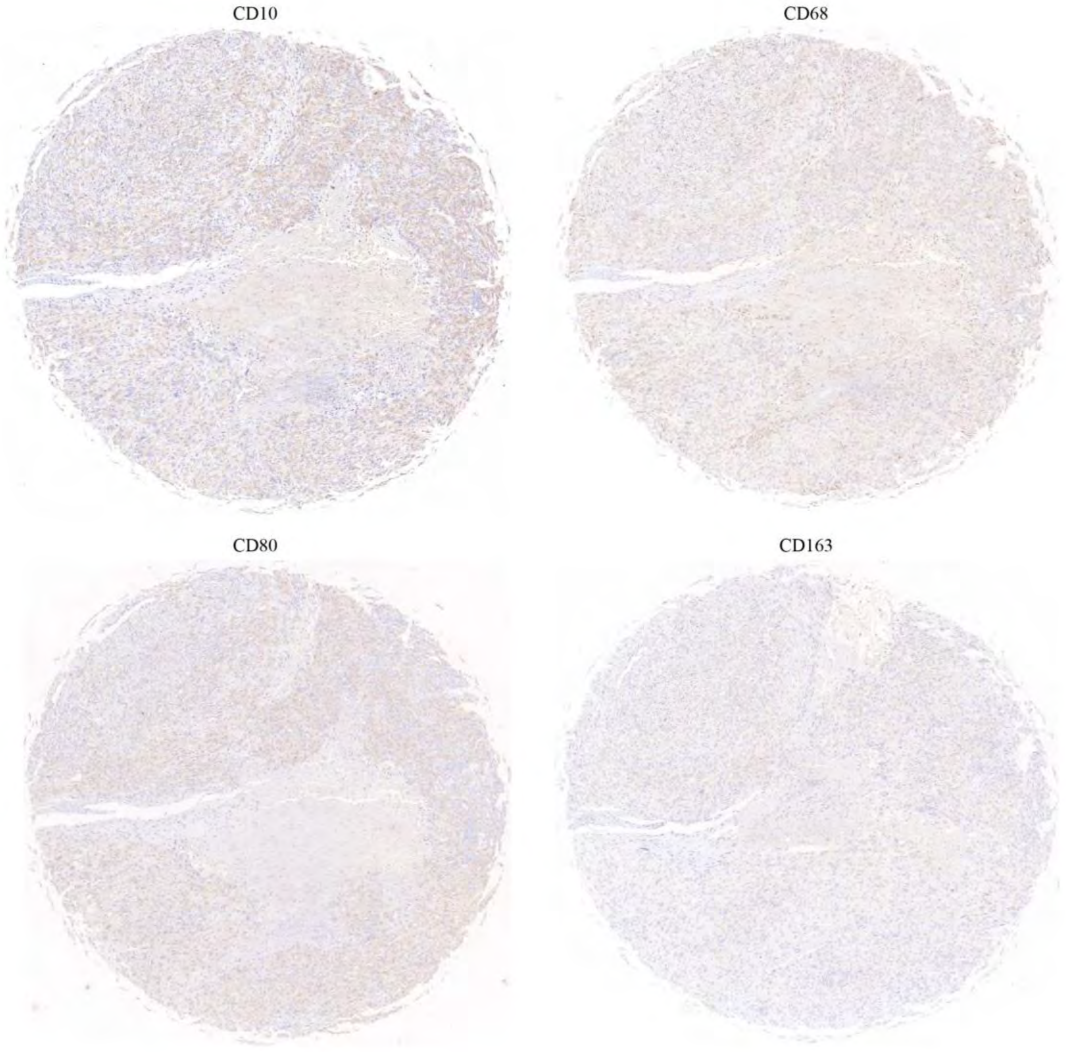

Case H05:

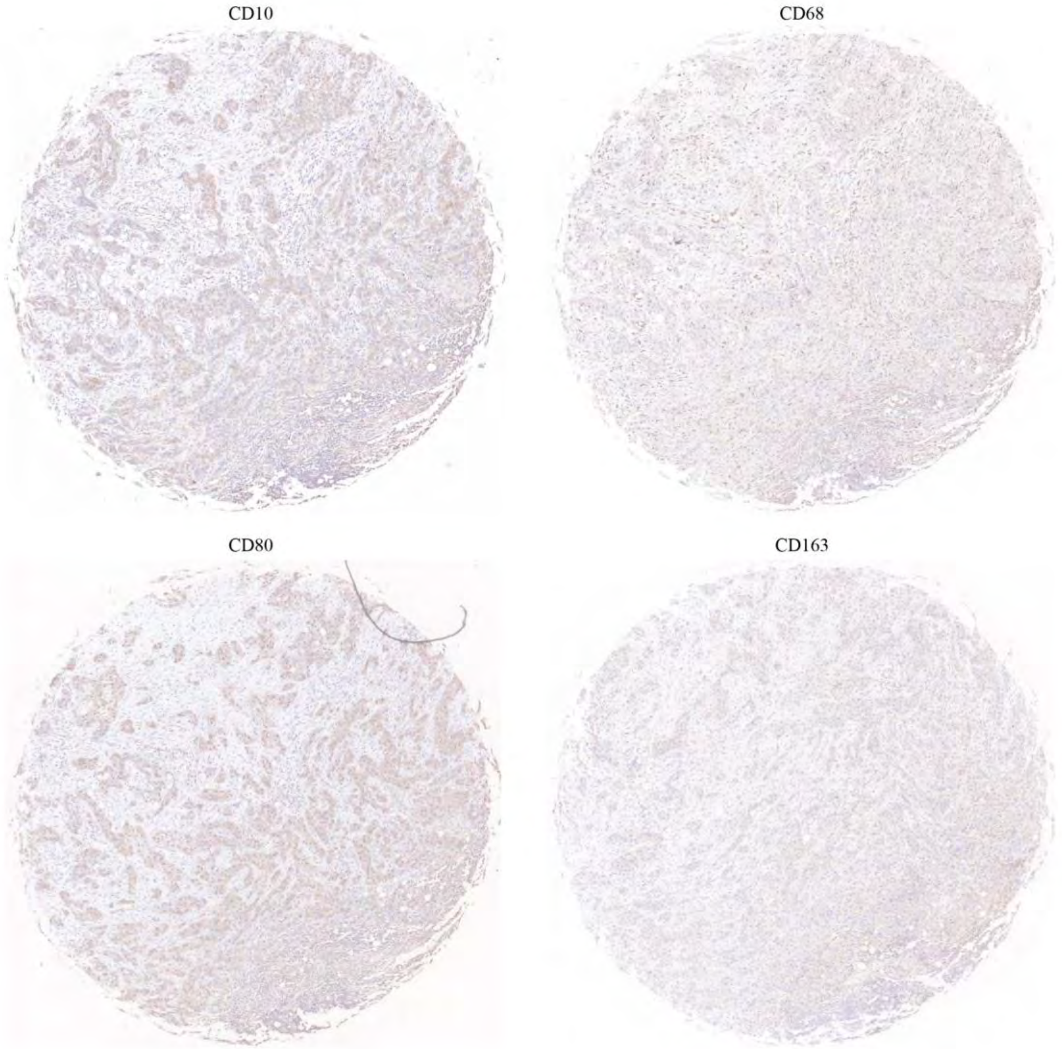

Case H07:

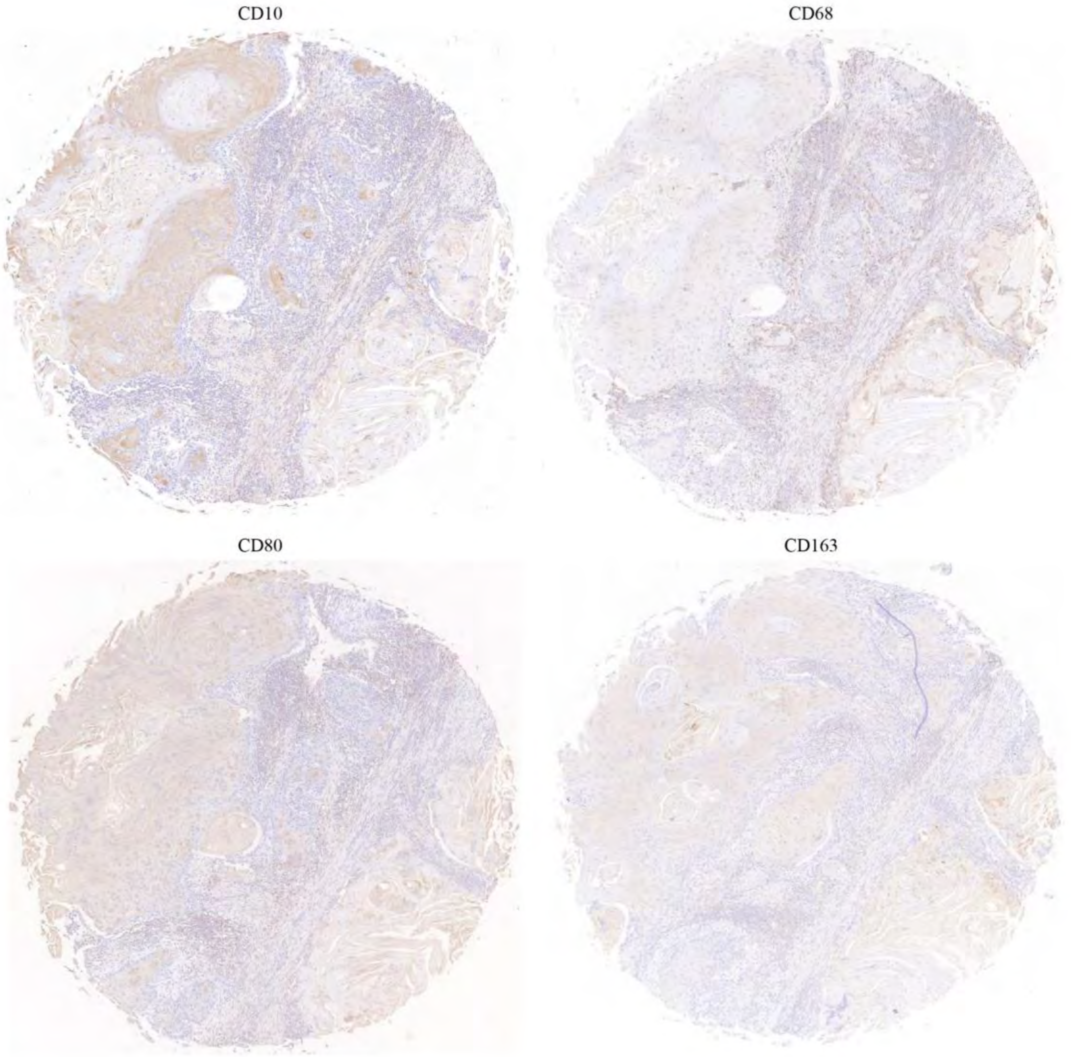

Case H09:

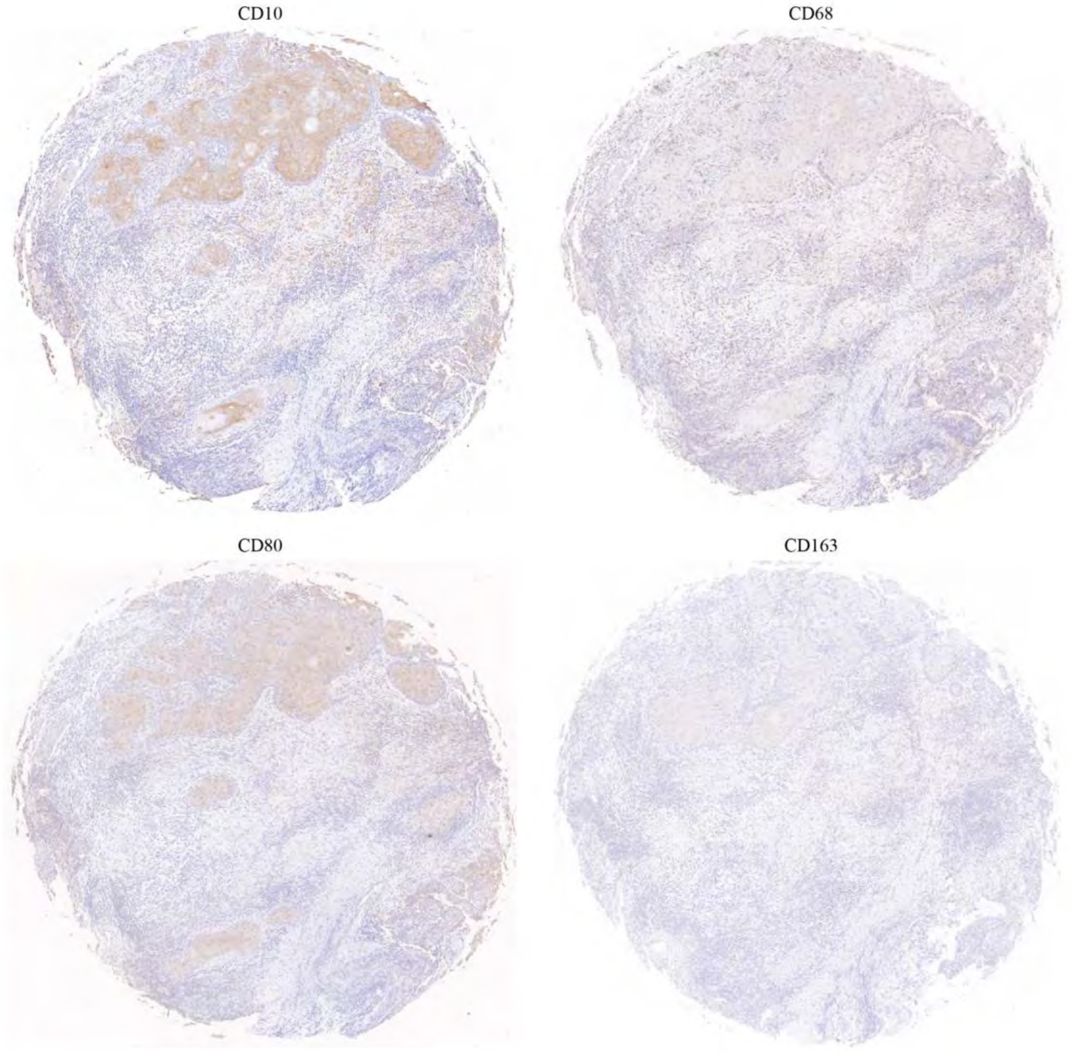

Case H11:

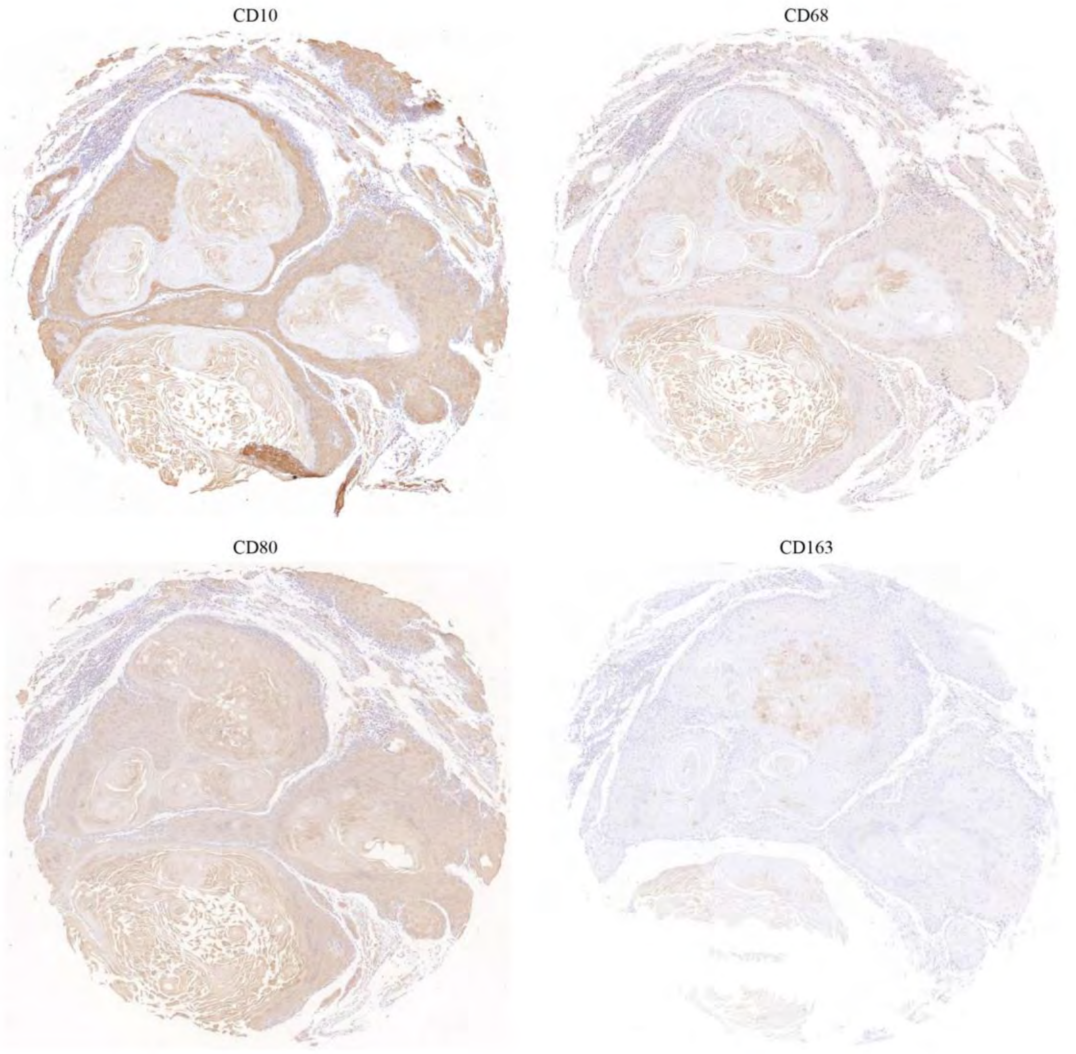

Supplement: Supplementary file 1 — Supplementary files. [file ijbsv21p1110s1.zip › supplementary/Supple File 3.pdf]
