# Supplementary material for: Tumor-associated macrophages drive heterogenetic CD10High cancer stem cells to implement tumor-associated neutrophils reprogramming in oral squamous cell carcinoma
Source: Int J Biol Sci. 2025 Jan 13;21(3):1110–26. doi: 10.7150/ijbs.100611 (PMC11781160; doi:10.7150/ijbs.100611)
Supplement: Supplementary file 1 — Supplementary files. [file ijbsv21p1110s1.zip › supplementary file legends.docx]

Supplementary File legends

Supplementary File 1 The expression distribution and the expression abundance of CD10 of in different cells based on OSCC single cell dataset (GSE103322).

Supplementary File 2 The mutation rate of CD10 in HNSCC.

Supplementary File 3 CD10, CD68, CD80, and CD63 IHC images of patients in the tissue microarray.

Supplementary File 4 (A) Expression of CD10 in OSCC cell lines was quantified by RT-qPCR; (B) Representative images of xenografts derived from HN6 and Cal27 cells, (C) HN6-CD10^High^ cells was sorted by FACS, (D) CD10 activity was detected in Cal27-CD10OE cells, ***p*<0.01; (E) Tumor volume of xenografts derived from HN6- CD10^High^ and HN6-CD10^Low^ cells at different seeding densities, ***p*<0.01; (F) Tumor weight of xenograft-derived Cal27-CD10^OE^ cells, ***p*<0.01.

Supplementary File 5 HN6-CD10^High^ cells sorted by MACS showed enhanced cancer stem cells (CSCs)-associated characteristics. HN6-CD10^High^ and HN6-CD10^Low^ cells were sorted by MACS and identified by western blotting (A), q-PCR (B), ****p*<0.001; The CSCs properties of CD10^High^ OSCC cells was detected by colony formation (C), soft agar colony formation(D), and microsphere formation (E) assays. Data were presented as the mean ± SD of three independent experiments; ***p*<0.01; (F) Representative images of xenografts derived from MACS-HN6-CD10^High^ cells and MACS-HN6-CD10^Low^ cells were shown. (F-G) Tumor weights (F) and volumes (G) were measured and were displayed on the right; ***p*<0.01.

Supplementary File 6 (A) CD10 activity was detected in HN6-shCD10 cells, ***p*<0.01; (B) CD10 expression was assessed after sacubitril treatment.

Supplementary File 7 Tumor weights of xenografts derived from HN6-shCD10 cells (A) and HN6 cells treated with sacubitril (B); ***p*<0.01.

Supplementary File 8 shCD10 attenuated the cancer stemness and malignant behaviors of OSCC cells. (A) Transfection efficiency of shCD10 in HN6 cells detected by Western blot; (B) CD10 activity was measured in HN6 cells transfected with shCD10, ***p*<0.01; (C-E) The CSCs properties of OSCC cells transfected with CD10 knockdown lentivirus was detected by colony formation (C), microsphere formation (D), and soft agar colony formation (E) assays. The data were presented as the means ± SDs of three independent experiments, ***p*<0.01.

Supplementary File 9 Percentage of CD10-positive in Cal27 and SCC25 cells treated with CM from TAMs.

Supplementary File 10 Concentration of IL6 in the medium of TAMs from the OSCC-educated and control groups; ***p*<0.01.

Supplementary File 11 WB analyses of STAT3 knockdown in SCC25 and Cal27 cells.

Supplementary File 12 The quantitative results for the IHC images of SCC25(A) and Cal27(B) xenografts.

Supplementary File 13 (A) Histological changes in HL60 cells after DMSO-induced differentiation; (B) Neutrophil marker expression validated after DMSO-induced differentiation; ***p*<0.01.

Supplementary File 14 Correlations between S100A8 and NOS2 (A), S100A8 and NOS2 (B), S100A9 and NOS2 (C), S100A9 and ARG1 (D).

Supplementary File 15 Percentage of PD1^+^ (A) and Tim3^+^ (B) CD8^+^ T cells.

Supplementary File 16 CD10 educated neutrophils could impair CD8^+^ T cells proliferation.
