# Supplementary figures and images for: Tumor-associated macrophages drive heterogenetic CD10High cancer stem cells to implement tumor-associated neutrophils reprogramming in oral squamous cell carcinoma
Source: Int J Biol Sci. 2025 Jan 13;21(3):1110–26. doi: 10.7150/ijbs.100611 (PMC11781160; doi:10.7150/ijbs.100611)

A

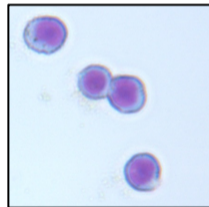

HL60

1.3% DMSO

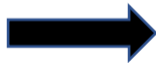

5 days

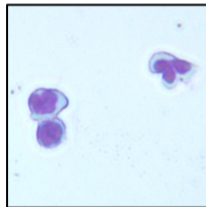

dHL60

B

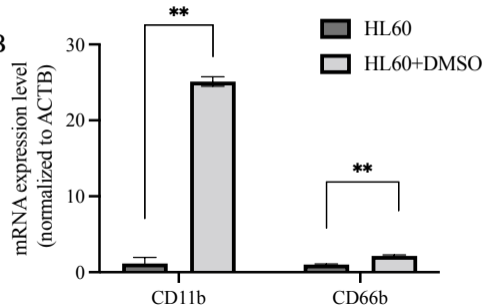

Supplement: Supplementary file 1 — Supplementary files. [file ijbsv21p1110s1.zip › supplementary/Supplementary file13.pdf]

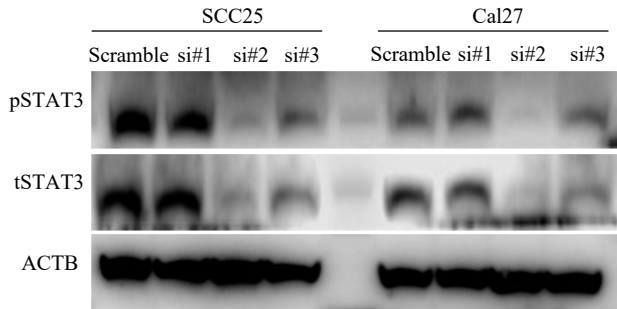

Supplement: Supplementary file 1 — Supplementary files. [file ijbsv21p1110s1.zip › supplementary/Supplementary file11.pdf]

A

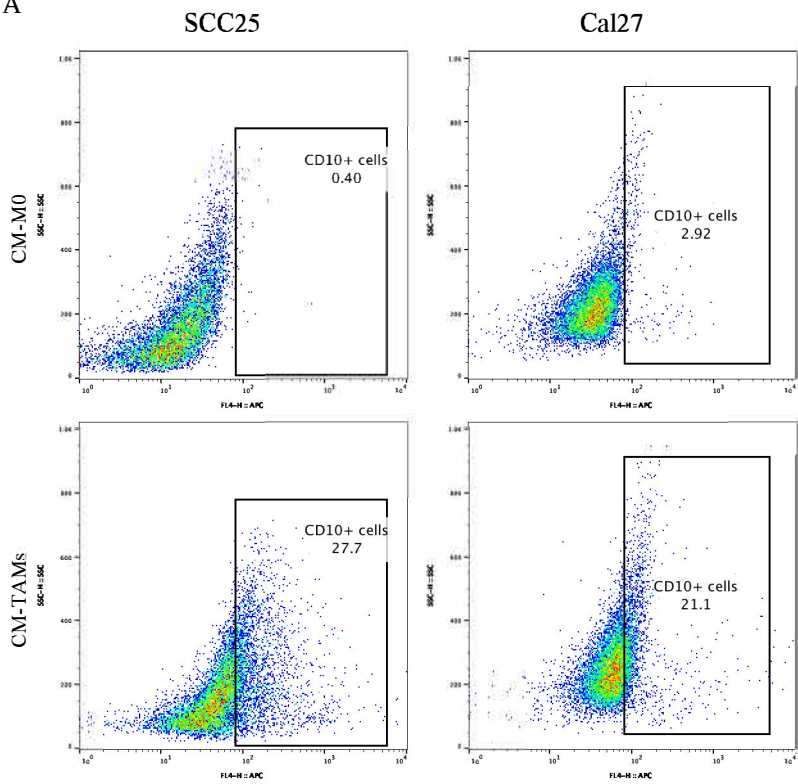

Supplement: Supplementary file 1 — Supplementary files. [file ijbsv21p1110s1.zip › supplementary/Supplementary file9.pdf]

A

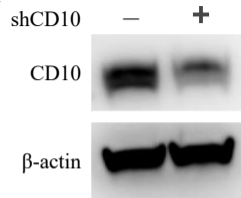

B

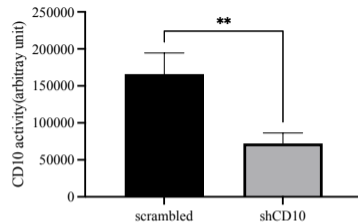

C

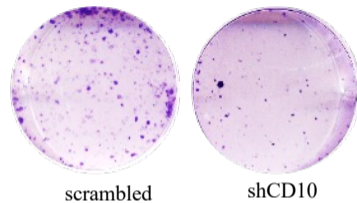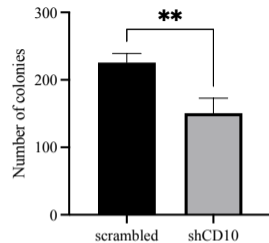

D

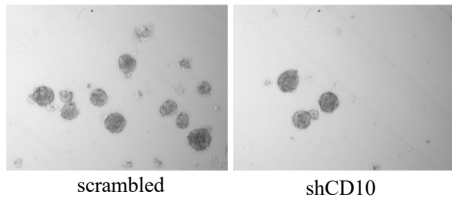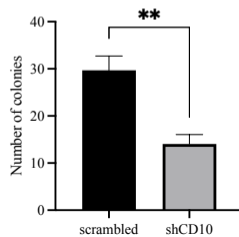

E

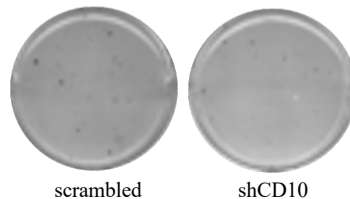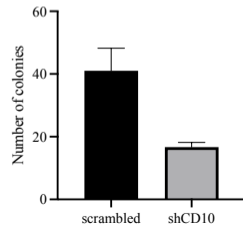

Supplement: Supplementary file 1 — Supplementary files. [file ijbsv21p1110s1.zip › supplementary/Supplementary file8.pdf]

A

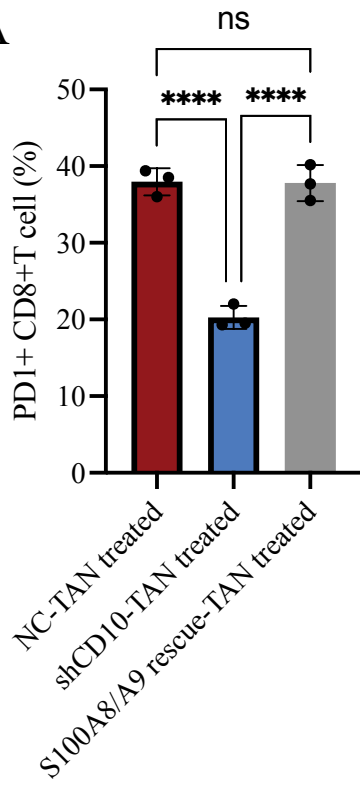

B

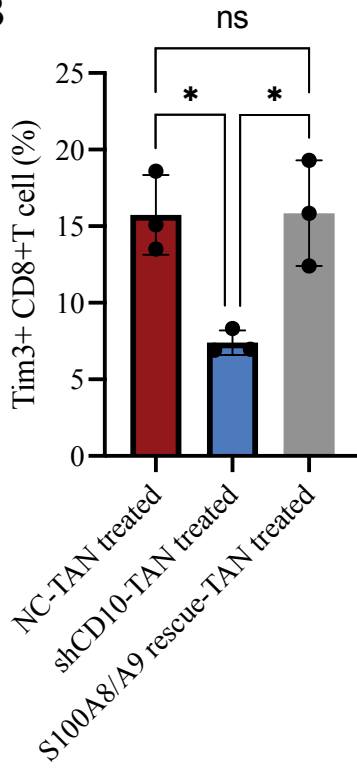

Supplement: Supplementary file 1 — Supplementary files. [file ijbsv21p1110s1.zip › supplementary/Supplementary File15.pdf]

A

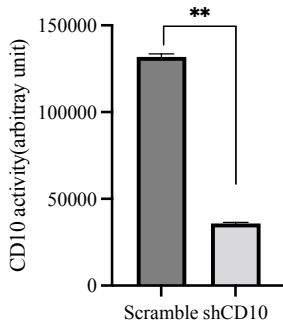

B

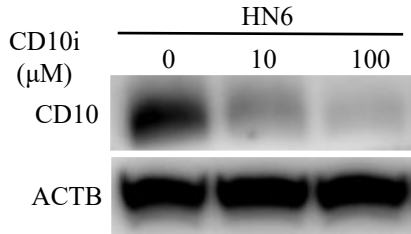

Supplement: Supplementary file 1 — Supplementary files. [file ijbsv21p1110s1.zip › supplementary/Supplementary file 6.pdf]

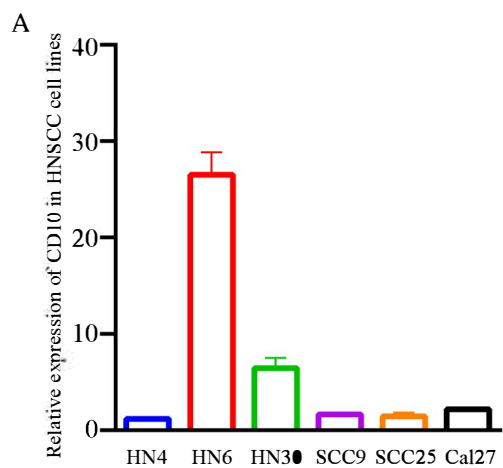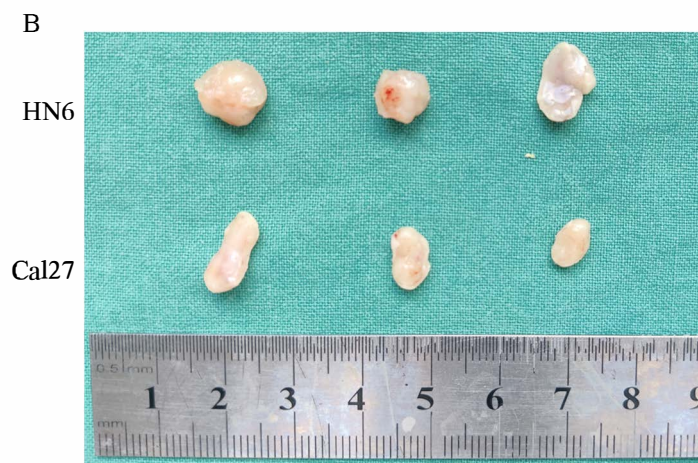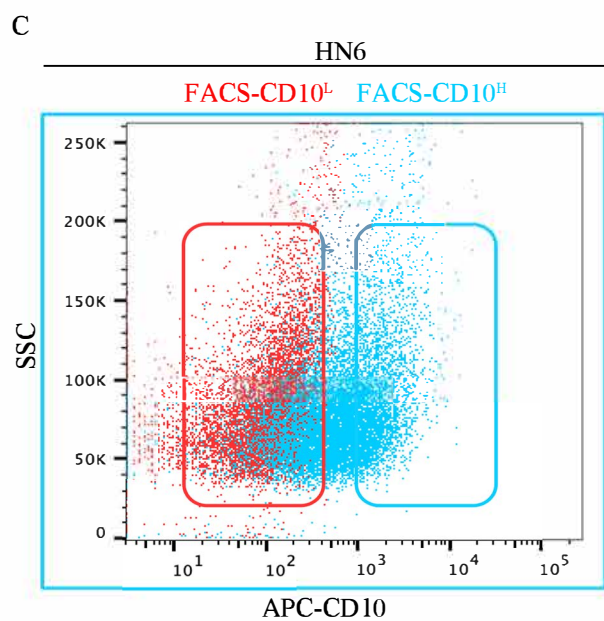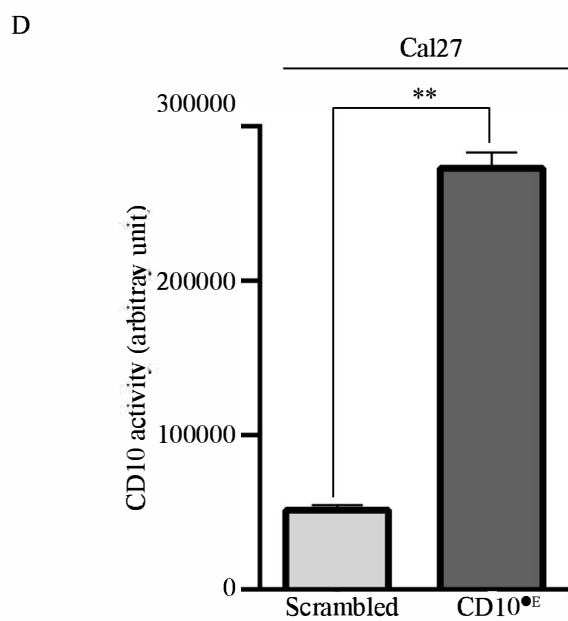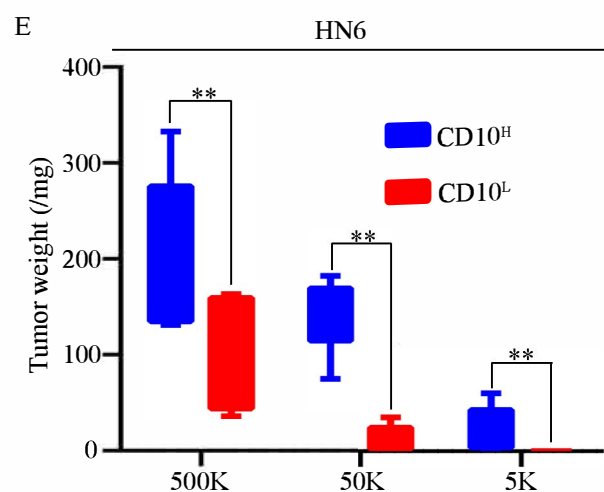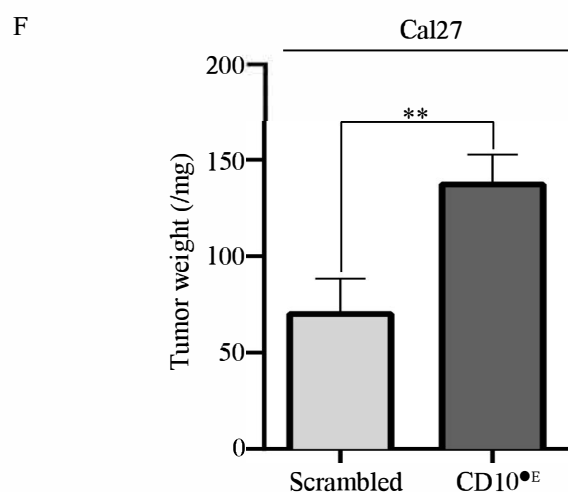

Supplement: Supplementary file 1 — Supplementary files. [file ijbsv21p1110s1.zip › supplementary/Supplementary file 4.pdf]

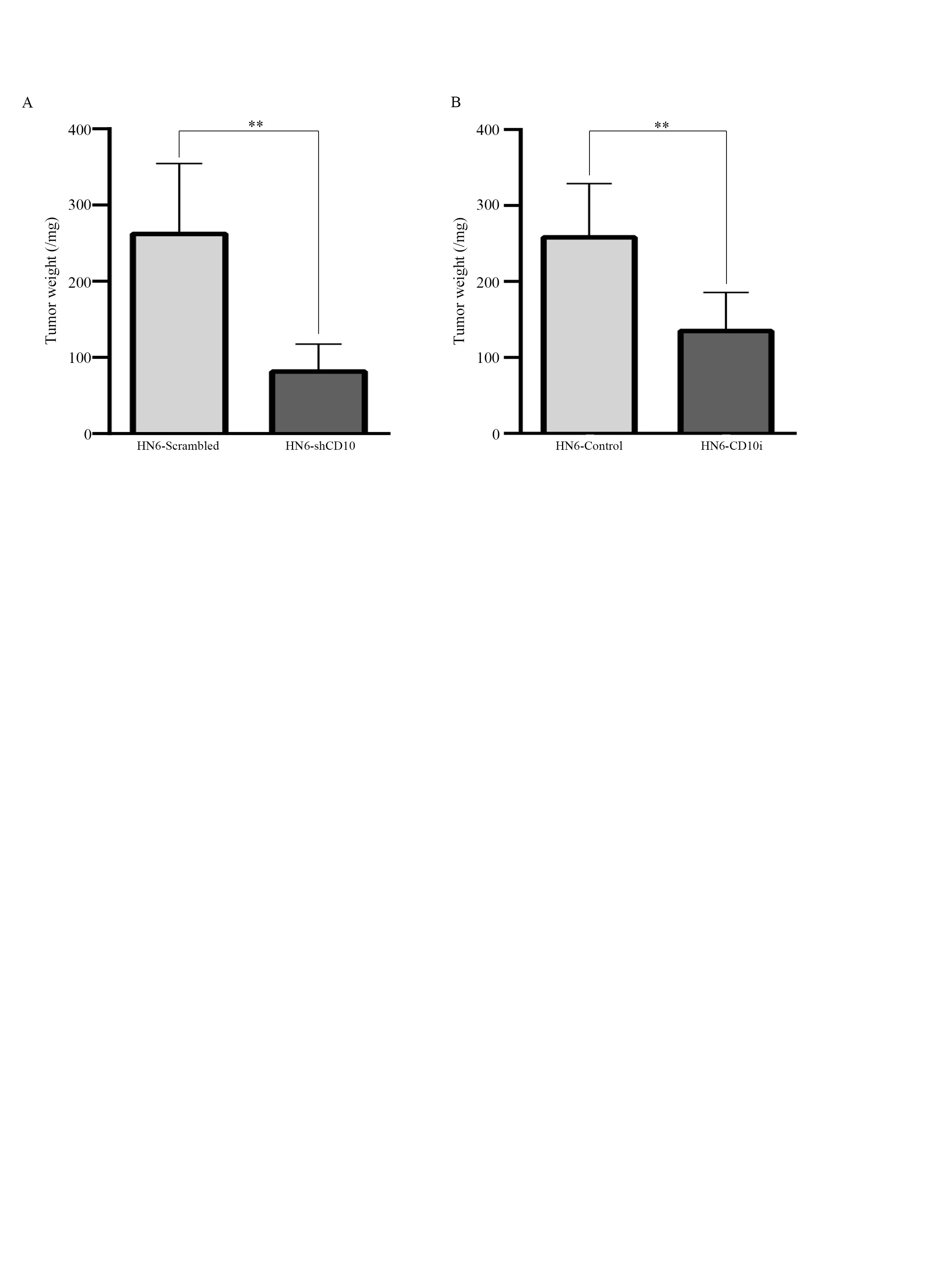

Supplement: Supplementary file 1 — Supplementary files. [file ijbsv21p1110s1.zip › supplementary/Supplementary file 7.jpg]

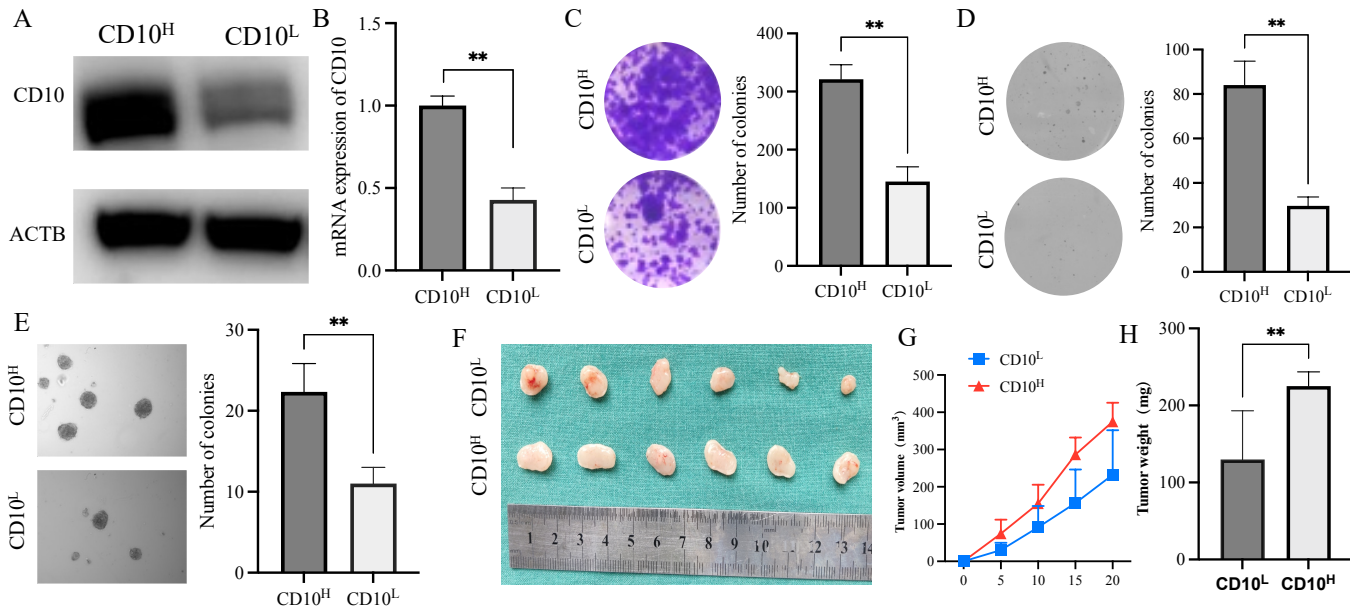

Supplement: Supplementary file 1 — Supplementary files. [file ijbsv21p1110s1.zip › supplementary/Supplementary file 5.pdf]

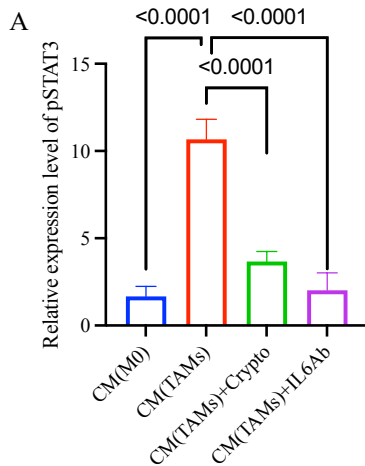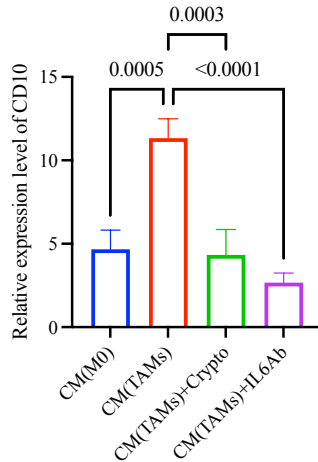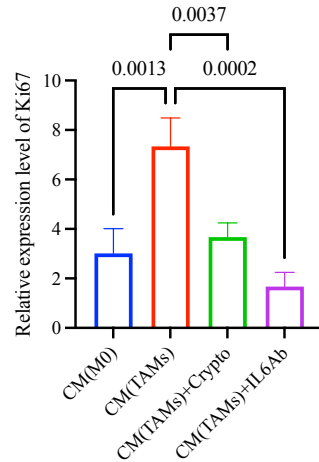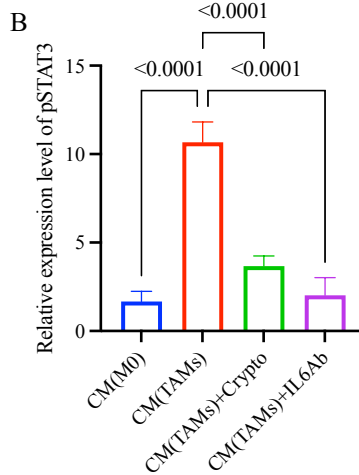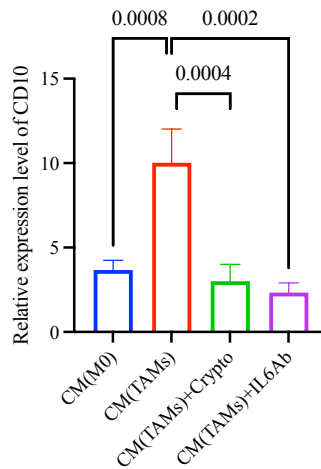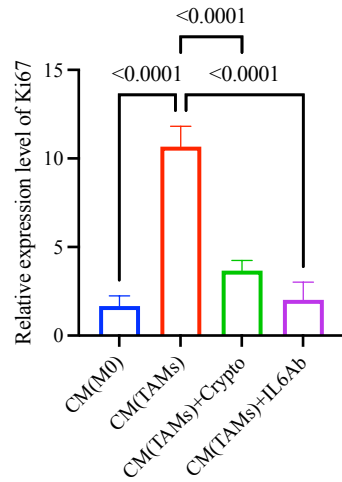

Supplement: Supplementary file 1 — Supplementary files. [file ijbsv21p1110s1.zip › supplementary/Supplementary file 12.pdf]

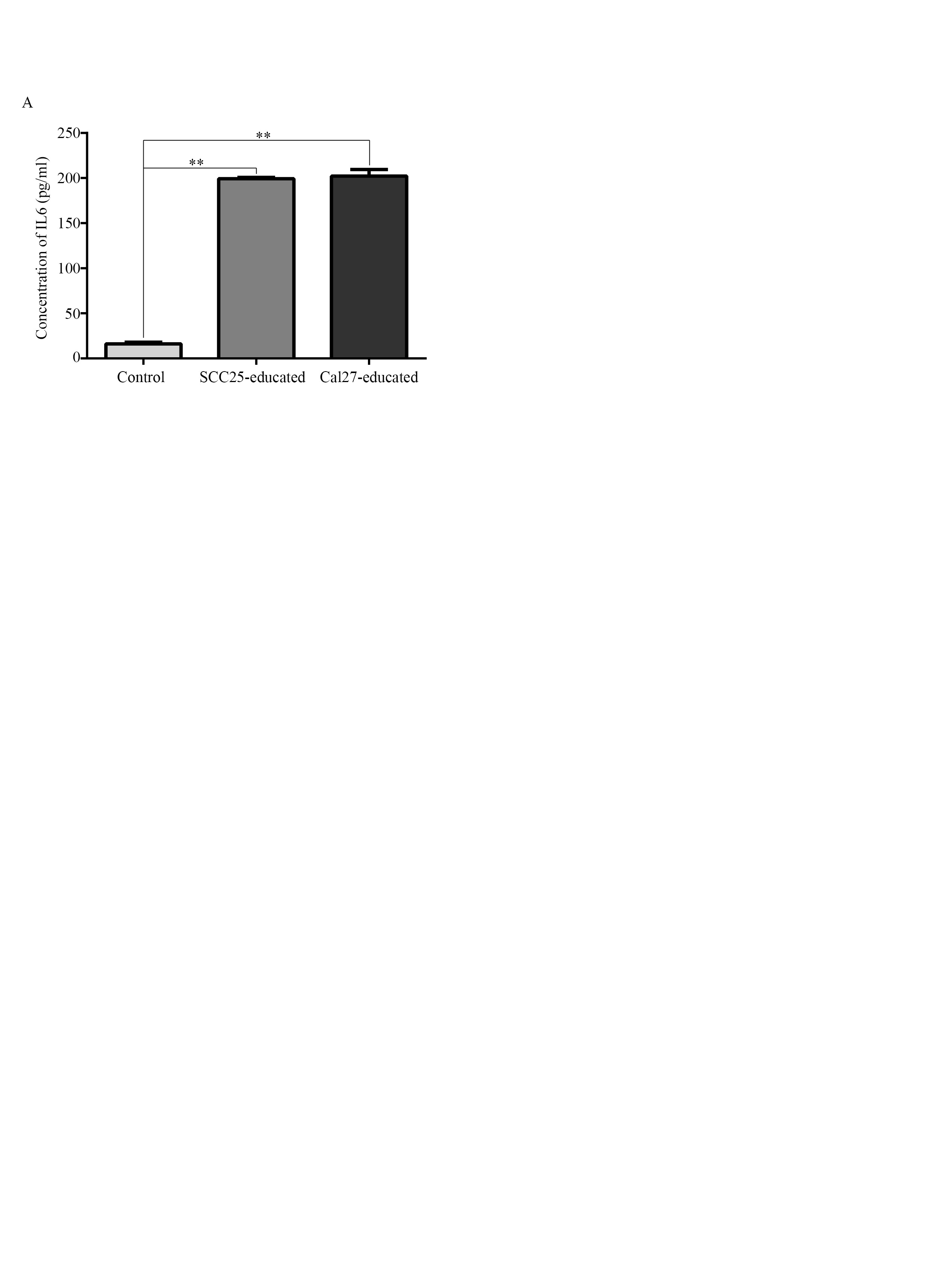

Supplement: Supplementary file 1 — Supplementary files. [file ijbsv21p1110s1.zip › supplementary/Supplementary file 10.jpg]

A

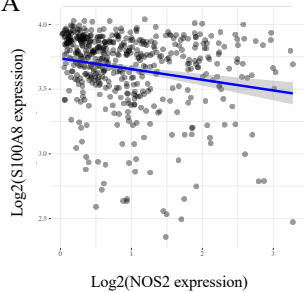

B

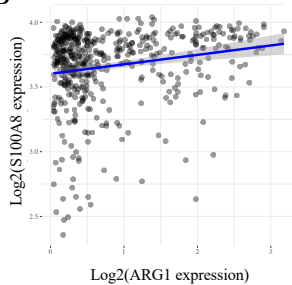

C

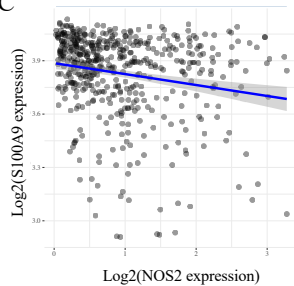

D

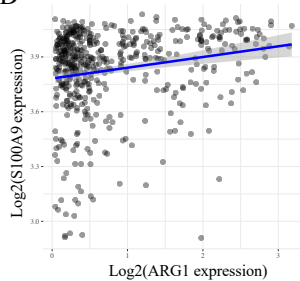

Supplement: Supplementary file 1 — Supplementary files. [file ijbsv21p1110s1.zip › supplementary/Supplementary file 14.pdf]

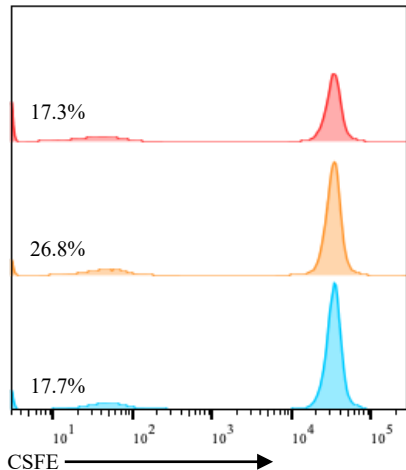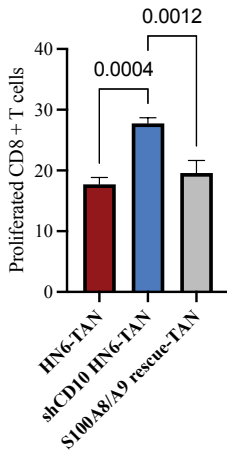

Supplement: Supplementary file 1 — Supplementary files. [file ijbsv21p1110s1.zip › supplementary/Supplementary file 16.pdf]

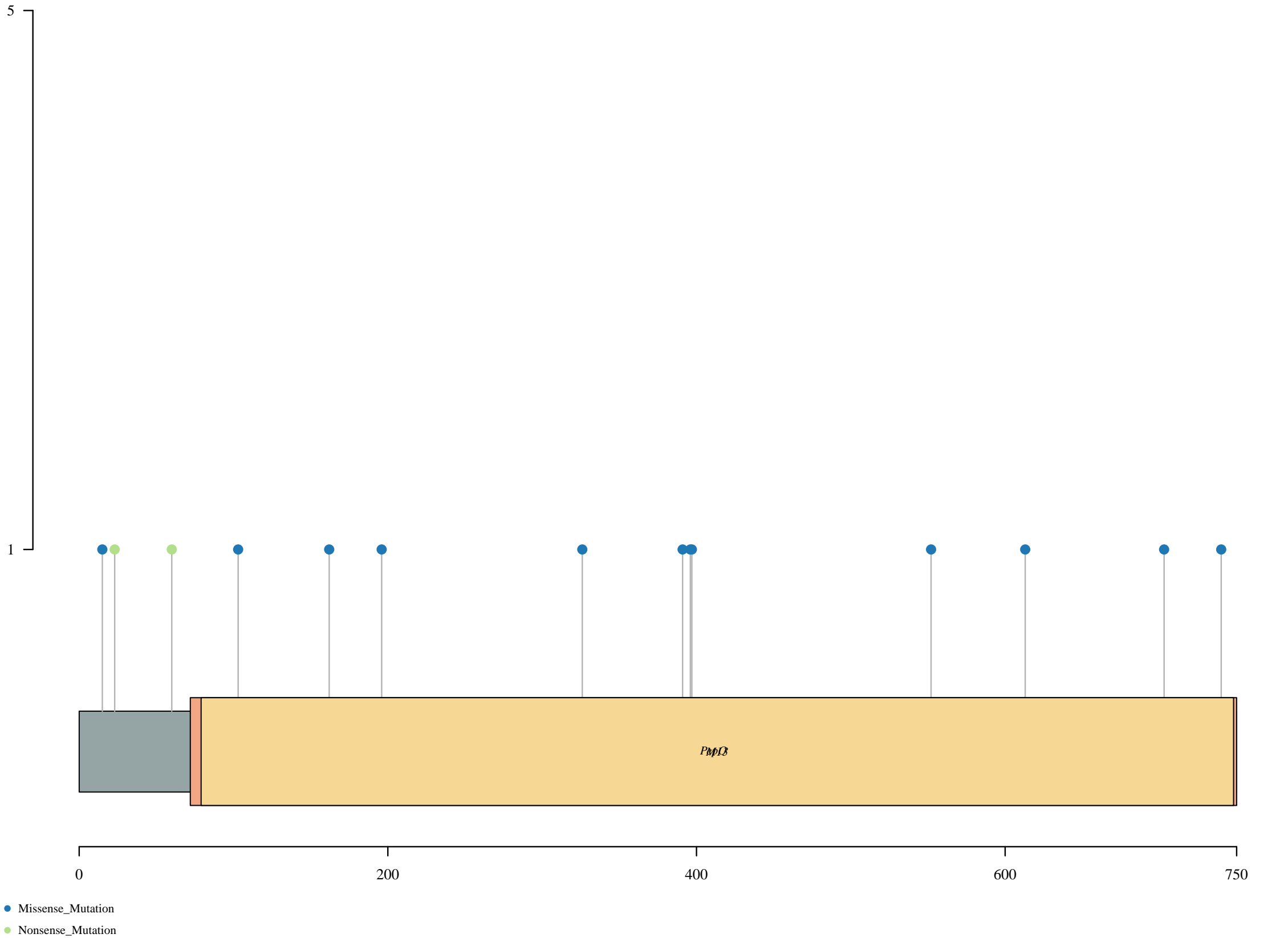

Supplement: Supplementary file 1 — Supplementary files. [file ijbsv21p1110s1.zip › supplementary/Supplementary file2.pdf]

Celltype major lineage

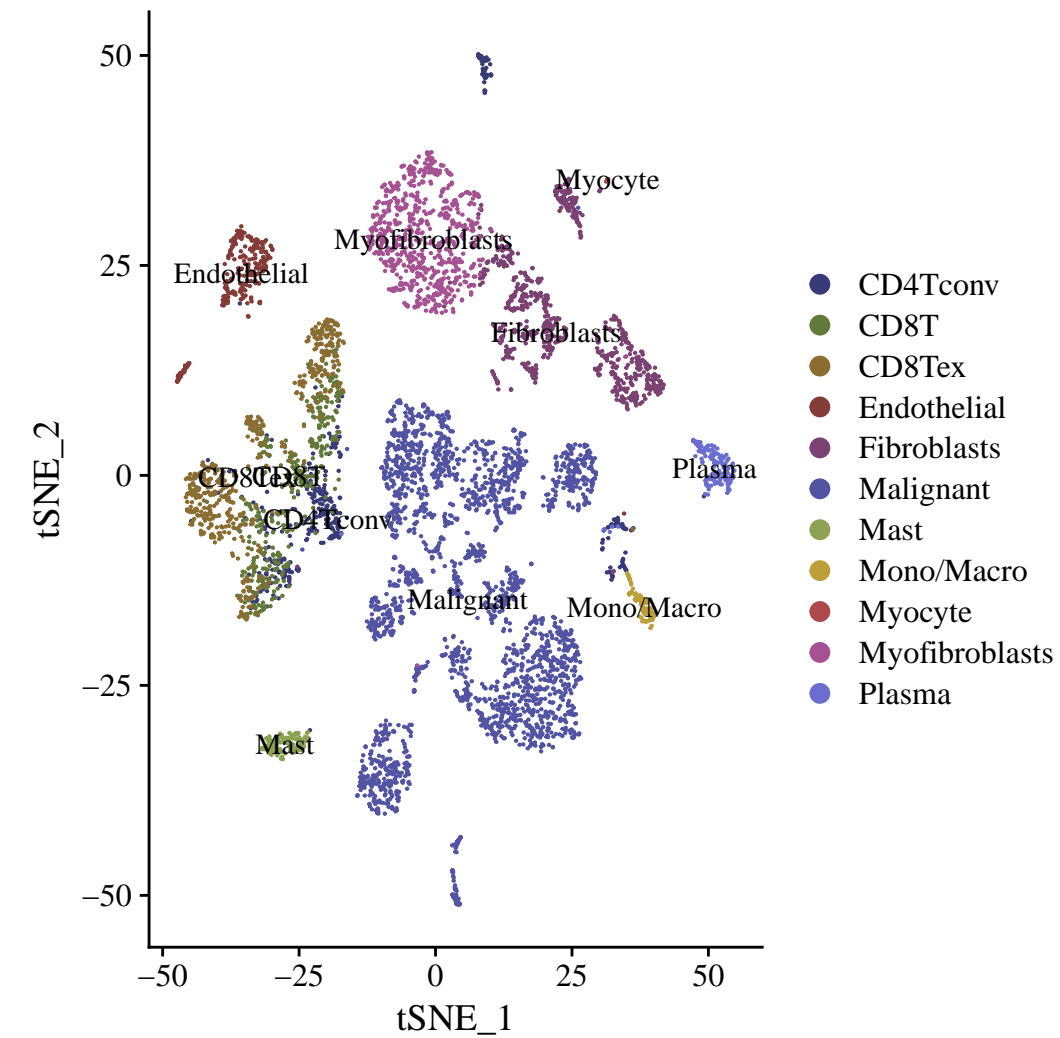

MME

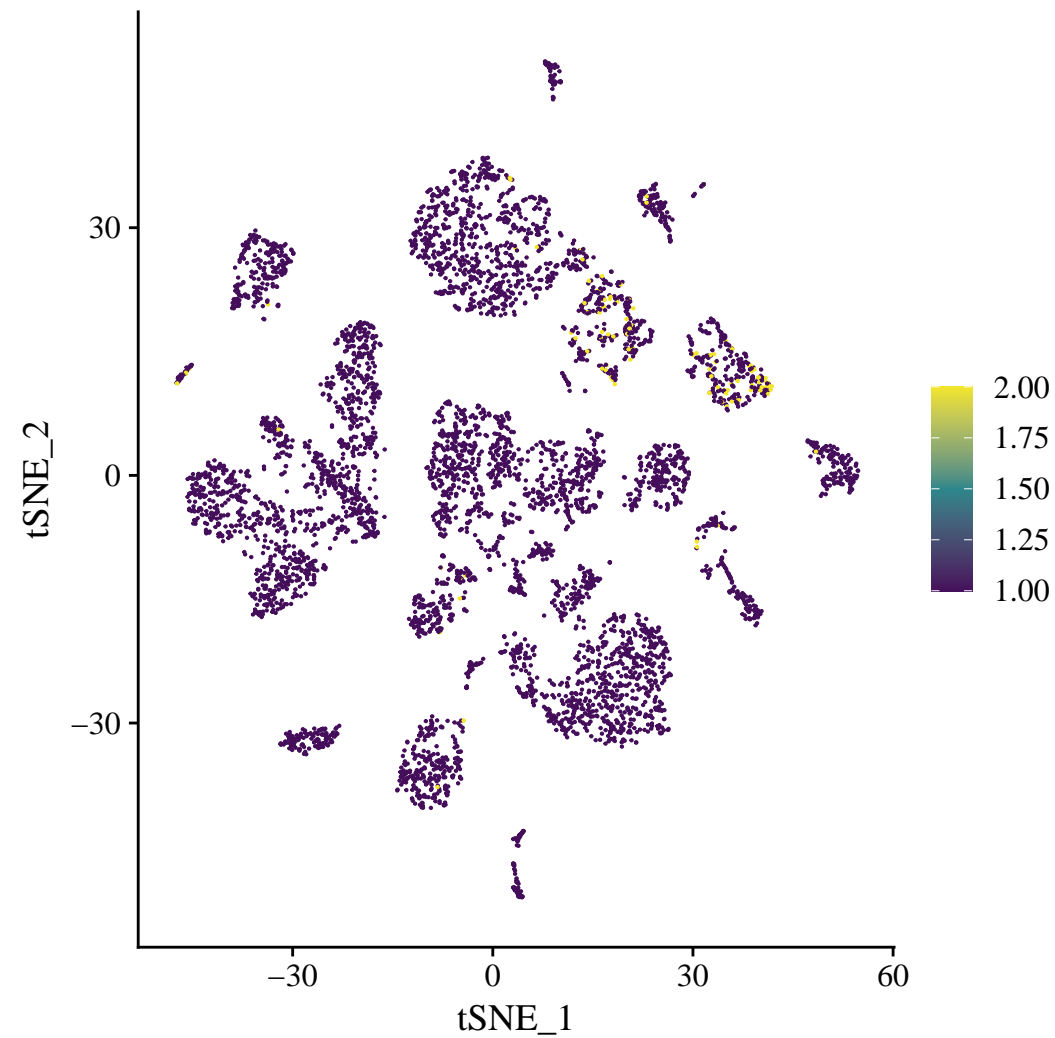

Mean Expression

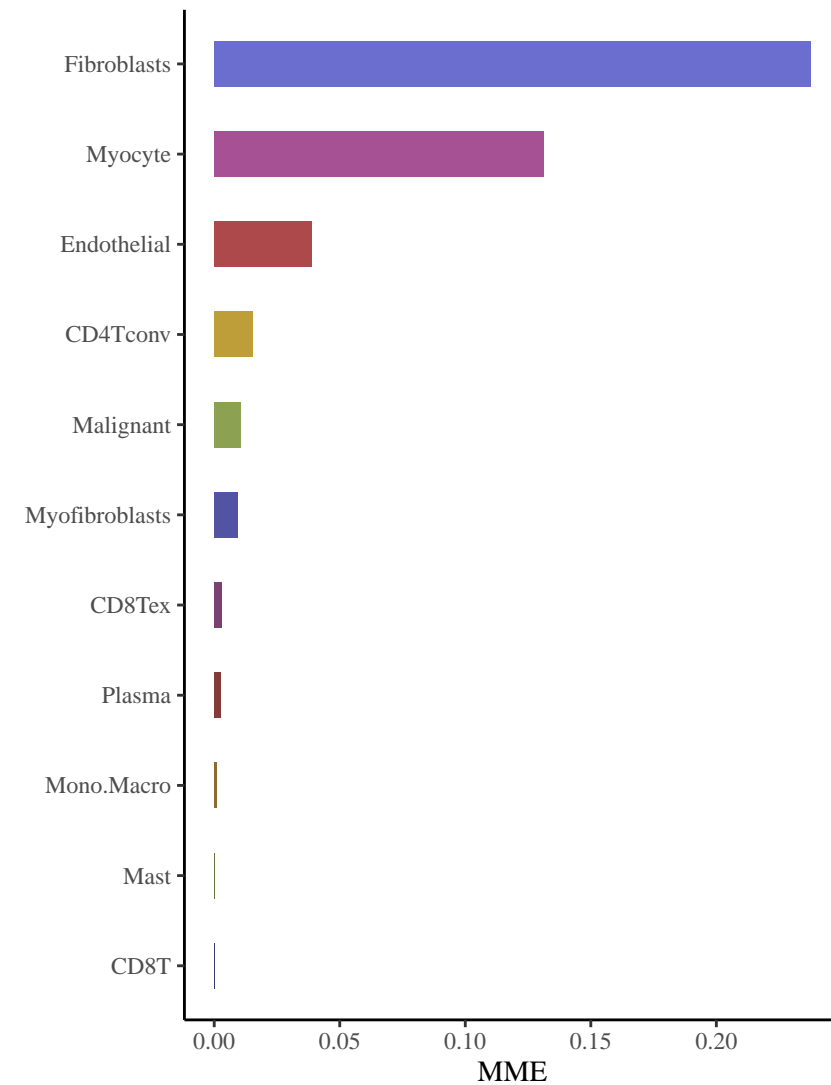

Supplement: Supplementary file 1 — Supplementary files. [file ijbsv21p1110s1.zip › supplementary/Supplementary file 1 revised.pdf]
